# Escape regimes of biased random walks on Galton–Watson trees

**DOI:** 10.1007/s00440-017-0768-y

**Published:** 2017-03-08

**Authors:** Adam Bowditch

**Affiliations:** University of Warwick, Coventry, UK

**Keywords:** Random walk in random environment, Galton–Watson tree, Infinite variance, Infinitely divisible distributions, Sub-ballistic, Primary 60K37, 60F05, Secondary 60E07, 60J80

## Abstract

We study biased random walk on subcritical and supercritical Galton–Watson trees conditioned to survive in the transient, sub-ballistic regime. By considering offspring laws with infinite variance, we extend previously known results for the walk on the supercritical tree and observe new trapping phenomena for the walk on the subcritical tree which, in this case, always yield sub-ballisticity. This is contrary to the walk on the supercritical tree which always has some ballistic phase.

## Introduction

In this paper, we investigate biased random walks on subcritical and supercritical Galton–Watson trees. These are a natural setting for studying trapping phenomena as dead-ends, caused by leaves in the trees, slow the walk. These models can be used to approach more difficult problems concerning biased random walks on percolation clusters (as studied in [11, 13, 24]) and random walk in random environment (see for example [18, 25]). For a recent review of trapping phenomena and random walk in random environment we direct the reader to [3] which covers recent developments in a range of models of directionally transient and reversible random walks on underlying graphs such as supercritical GW-trees and supercritical percolation clusters.

For supercritical GW-trees with leaves, it has been shown in [21] that, for a suitably large bias away from the root, the dead-ends in the environment create a sub-ballistic regime. In this case, it has further been observed in [4], that if the offspring distribution has finite variance then the walker follows a polynomial escape regime but cannot be rescaled properly due to a certain lattice effect. (In [5, 15] it is shown that, in a related model where the conductance along each is chosen randomly according to a distribution satisfying a certain non-lattice assumption, the tail of the trapping time obeys a pure power law and the rescaled walk converges in distribution.) Here we show that, when the offspring law has finite variance, the walk on the subcritical GW-tree conditioned to survive experiences similar trapping behaviour to the walk on the supercritical GW-tree shown in [4]. However, the main focus of the article concerns offspring laws belonging to the domain of attraction of some stable law with index $\alpha \in (1,2)$. In this setting, although the distribution of time spent in individual traps has polynomial tail decay in both cases, the exponent varies with $\alpha$ in the subcritical case and not in the supercritical case. This results in a polynomial escape of the walk which is always sub-ballistic in the subcritical case unlike the supercritical case which always has some ballistic phase.

We now describe the model of a biased random walk on a subcritical GW-tree conditioned to survive which will be the main focus of the article. Let $f(s):=\sum _{k=0}^\infty p_ks^k$ denote the probability generating function of the offspring law of a GW-process with mean $\mu >0$ and variance $\sigma ^2>0$ (possibly infinite) and let $Z_n$ denote the *n*th generation size of a process with this law started from a single individual, i.e. $Z_0=1$. Such a process gives rise to a random tree where individuals in the process are represented by vertices and undirected edges connect individuals with their offspring.

For a fixed tree *T* let $\rho$ denote its root, $Z_n^T$ the size of the *n*th generation, $\overleftarrow{x}$ the parent of $x \in T$, *c*(*x*) the set of children of *x*, $d_x:=|c(x)|$ the out-degree of *x*, *d*(*x*, *y*) the graph distance between vertices *x*, *y*, $|x|:=d(\rho ,x)$ the graph distance between *x* and the root and $T_x$ to be the descendent tree of *x*. A $\beta$-biased random walk on a fixed, rooted tree *T* is a random walk $(X_n)_{n \ge 0}$ on *T* which is $\beta$-times more likely to make a transition to a given child of the current vertex than the parent (which are the only options). That is, the random walk is the Markov chain started from $X_0=z$ defined by the transition probabilities

$$\begin{aligned} P ^T_z(X_{n+1}=y|X_n=x):={\left\{ \begin{array}{ll} \frac{1}{1+\beta d_x} &{} \text {if } y=\overleftarrow{x}, \\ \frac{\beta }{1+\beta d_x}, &{} \text {if } y \in c(x), \; x \ne \rho , \\ \frac{1}{ d_\rho }, &{} \text {if } y \in c(x), \; x =\rho , \\ 0, &{} \text {otherwise.} \\ \end{array}\right. } \end{aligned}$$

We use $\mathbb {P}_\rho (\cdot ):=\int P ^T_\rho (\cdot )\mathbf {P}(\text {d}T)$ for the annealed law obtained by averaging the quenched law $P ^T_\rho$ over a law $\mathbf {P}$ on random trees with a fixed root $\rho$. In general we will drop the superscript *T* and subscript $\rho$ when it is clear to which tree we are referring and we start the walk at the root.

We will mainly be interested in GW-trees $\mathcal {T}$ which survive, that is $\mathcal {H}(\mathcal {T}):=\sup \{n\ge 0: Z_n>0\}=\infty$. It is classical (e.g. [2]) that when $\mu >1$ there is some strictly positive probability $1-q$ that $\mathcal {H}(\mathcal {T})=\infty$ whereas when $\mu \le 1$ we have that $\mathcal {H}(\mathcal {T})$ is almost surely finite. However, it has been shown in [17] that there is some well defined probability measure $\mathbf {P}$ over *f*-GW trees conditioned to survive for infinitely many generations which arises as a limit of probability measures over *f*-GW trees conditioned to survive at least *n* generations. Henceforth, we assume $\mathbf {P}$ is this law and $X_n$ is a random walk on an *f*-GW-tree conditioned to survive.

The main object of interest is $|X_n|$, that is, how the distance from the root changes over time. Due to the typical size of finite branches in the tree being small and the walk not backtracking too far we shall see that $|X_n|$ has a strong inverse relationship with the first hitting times $\Delta _n:=\inf \{m\ge 0:X_m \in \mathcal {Y}, \; |X_m|=n\}$ of levels along the backbone $\mathcal {Y}:=\{x \in \mathcal {T}:\mathcal {H}(\mathcal {T}_x)=\infty \}$ so for much of the paper we will consider this instead. It will be convenient to consider the walk as a trapping model. To this end we define the underlying walk $(Y_k)_{k\ge 0}$ defined by $Y_k:=X_{\eta _k}$ where $\eta _0:=0$ and $\eta _k:=\inf \{m>\eta _{k-1}: \; X_m,X_{m-1} \in \mathcal {Y}\}$ for $k\ge 1$.

When $X_n$ is a walk on an *f*-GW tree conditioned to survive for *f* supercritical ($\mu >1$), it has been shown in [21] that $\nu (\beta ):=\lim _{n \rightarrow \infty }|X_n|/n$ exists $\mathbb {P}$-a.s. and is positive if and only if $\mu ^{-1}<\beta <f'(q)^{-1}$ in which case we call the walk ballistic. Furthermore, although no explicit expression for the speed $\nu$ is known, a description of the invariant distribution of the environment seen from the particle is used in [1] to give an expression of the speed in terms of the annealed expectation. This expression coincides with the speed of the walk on a certain regular tree where each vertex has some number of children $m_\beta$; in particular, it can be seen that $m_\beta \le \mu$ therefore the randomness of the tree slows the walk. If $\beta \le \mu ^{-1}$ then the walk is recurrent because the average drift of *Y* acts towards the root. When $\beta \ge f'(q)^{-1}$ the walker expects to spend an infinite amount of time in the finite trees which hang off $\mathcal {Y}$ (see Fig. 5 in Sect. 10) thus causing a slowing effect which results in the walk being sub-ballistic. In this case, the correct scaling for some non-trivial limit is $n^{\gamma }$ where $\gamma$ will be defined later in (1.1). In particular, it has been shown in [4] that, when $\sigma ^2<\infty$, the laws of $|X_n|n^{-\gamma }$ are tight and, although $|X_n|n^{-\gamma }$ doesn’t converge in distribution, we have that $\Delta _nn^{-1/\gamma }$ converges in distribution under $\mathbb {P}$ along certain subsequences to some infinitely divisible law. In Sect. 10 we extend this result by relaxing the condition that the offspring law has finite variance and instead requiring only that it belongs to the domain of attraction of some stable law of index $\alpha >1$.

Recall that the offspring law of the process is given by $\mathbf {P}(\xi =k):=p_k$, then we define the size-biased distribution by the probabilities $\mathbf {P}(\xi ^*=k):=kp_k\mu ^{-1}$. It can be seen (e.g. [16]) that the subcritical ($\mu <1$) GW-tree conditioned to survive coincides with the following construction: Starting with a single special vertex, at each generation let every normal vertex give birth onto normal vertices according to independent copies of the original offspring distribution and every special vertex give birth onto vertices according to independent copies of the size-biased distribution, one of which is chosen uniformly at random to be special. Unlike the supercritical tree which has infinitely many infinite paths, the backbone of the subcritical tree conditioned to survive consists of a unique semi-infinite path from the initial vertex $\rho$. We call the vertices not on $\mathcal {Y}$ which are children of vertices on $\mathcal {Y}$ buds and the finite trees rooted at the buds traps (see Fig. 2 in Sect. 3). In this paper we consider walks with positive bias therefore the walk is transient and only returns to the starting vertex $\rho$ finitely often. Moreover, we are interested in the case where the trapping times are heavy tailed and therefore since the traps are i.i.d. the walk closely resembles a one dimensional directed trap model as studied in [26].

Briefly, the phenomena that can occur in the subcritical case are as follows. When $\mathbf {E}[\xi \log ^+(\xi )]<\infty$ and $\mu <1$ there exists a limiting speed $\nu (\beta )$ such that $|X_n|/n$ converges almost surely to $\nu (\beta )$ under $\mathbb {P}$; moreover, the walk is ballistic ($\nu (\beta )>0$) if and only if $1<\beta <\mu ^{-1}$ and $\sigma ^2<\infty$. This essentially follows from the argument used in [21] (to show the corresponding result on the supercritical tree) with the fact that, by (2.1) and (5.2), the conditions given are precisely the assumptions needed so that the expected time spent in a branch is finite (see [8] or [9] for further detail). The sub-ballistic regime has four distinct phases. When $\beta \le 1$ the walk is recurrent and we are not concerned with this case here. When $1<\beta <\mu ^{-1}$ and $\sigma ^2=\infty$ the expected time spent in a trap is finite and the slowing of the walk is due to the large number of buds. When $\beta \mu >1$ and $\sigma ^2<\infty$, the expected time spent in a subcritical GW-tree forming a trap is infinite because the strong bias forces the walk deep into traps and long sequences of movements against the bias are required to escape. In the final case for the subcritical tree ($\beta \mu >1$, $\sigma ^2=\infty$) slowing effects are caused by both strong bias and the large number of buds.

**Fig. 1:**
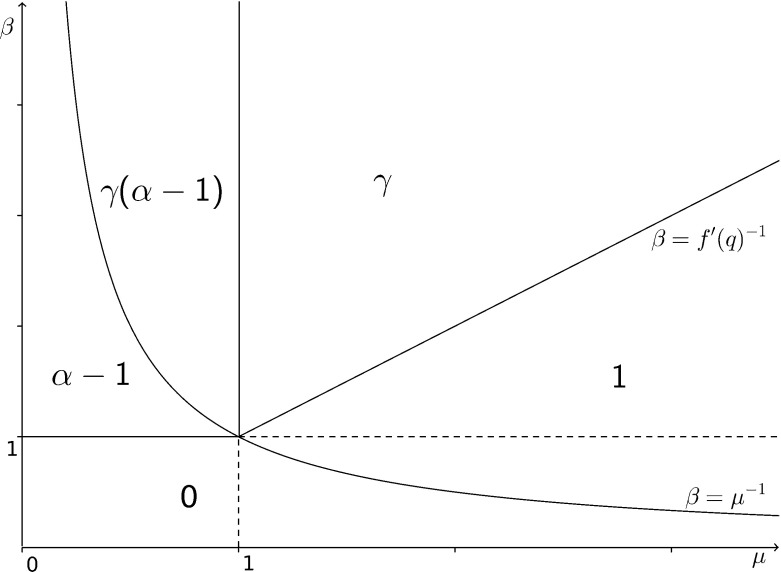
Phase diagram for the leading order polynomial exponent in the scaling of the walk relative to the mean of the offspring law and bias of the walk

Figure 1 is the phase diagram for the almost sure limit of $\log (|X_n|)/\log (n)$ (which is the leading order polynomial exponent in the scaling of $|X_n|$ relative to $\beta$ and $\mu$) where the offspring law has stability index $\alpha$ (which is 2 when $\sigma ^2<\infty$) and we define

1.1 $$\begin{aligned} \gamma :={\left\{ \begin{array}{ll} \frac{\log \left( f'(q)^{-1}\right) }{\log (\beta )}, &{} \mu >1,\\ \frac{\log \left( \mu ^{-1}\right) }{\log (\beta )}, &{} \mu < 1, \end{array}\right. } \end{aligned}$$

where we note that $f'(q)$ and $\mu$ are the mean number of offspring from vertices in traps of the supercritical and subcritical trees respectively. Strictly, $f'(q)$ isn’t a function of $\mu$ therefore the line $\beta =f'(q)^{-1}$ is not well defined; Fig. 1 shows the particular case when the offspring distribution belongs to the geometric family. It is always the case that $f'(q)<1$ therefore some such region always exists however the parametrisation depends on the family of distributions.

When the offspring law has finite variance, the limiting behaviour of $|X_n|$ on the supercritical and subcritical trees is very similar. Both have a regime with linear scaling (which is, in fact, almost sure convergence of $|X_n|/n$) and a regime with polynomial scaling caused by the same phenomenon of deep traps (which results in $|X_n|n^{-\gamma }$ not converging). When the offspring law has infinite variance, the bud distribution of the subcritical tree has infinite mean which causes an extra slowing effect which isn’t seen with the supercritical tree. This equates for the different exponents observed in the two models as shown in Fig. 1. The walk on the critical ($\mu =1$) tree experiences a similar trapping mechanism to the subcritical tree; however, the slowing is more extreme and belongs to a different universality class which had been shown in [10] to yield a logarithmic escape rate.

## Statement of main theorems and proof outline

In this section we introduce the three sub-ballistic regimes in the subcritical case and the one further regime for the infinite variance supercritical case that we consider here. We then state the main theorems of the paper.

The subcritical tree has bud distribution $\xi ^*-1$ where $\mathbf {P}(\xi ^*=k)=kp_k\mu ^{-1}$ which yields the following important property relating the size biased and offspring distributions

2.1 $$\begin{aligned} \mathbf {E}\left[ \varphi (\xi ^*)\right] =\sum _{k=1}^\infty \varphi (k)\frac{kp_k}{\mu }=\mathbf {E}\left[ \varphi (\xi )\xi \right] \mu ^{-1}. \end{aligned}$$

Choosing $\varphi$ to be the identity we have finite mean of the size-biased distribution if and only if the variance of the offspring distribution is finite. This causes a phase transition for the walk that isn’t seen in the supercritical tree. The reason for this is that in the corresponding decomposition for the supercritical tree we have subcritical GW-trees as leaves but the number of buds is exponentially tilted and therefore maintains moment properties.

If the offspring law belongs to the domain of attraction of some stable law of index $\alpha \in (1,2)$ then taking $\varphi (x)=x\mathbf {1}_{\{x\le t\}}$ shows that the size biased distribution belongs to the domain of attraction of some stable law with index $\alpha -1$ and allows us to attain properties of the scaling sequences (see for example [12, IX.8]).

The first case we consider is when $\beta \mu <1$ but $\sigma ^2=\infty$; we refer to this as the infinite variance, finite excursion case:

### Definition 1

(*IVFE*) The offspring distribution has mean $\mu$ satisfying $1<\beta <\mu ^{-1}$ and belongs to the domain of attraction of a stable law of index $\alpha \in (1,2)$.

Under this assumption we let *L* vary slowly at $\infty$ such that as $x\rightarrow \infty$

2.2 $$\begin{aligned} \mathbb {E}\left[ \xi ^2\mathbf {1}_{\left\{ \xi \le x\right\} }\right] \sim x^{2-\alpha }L(x) \end{aligned}$$

and choose $(a_n)_{n\ge 1}$ to be some scaling sequence for the size-biased law such that for any $x>0$, as $n \rightarrow \infty$ we have $\mathbf {P}(\xi ^*\ge xa_n) \sim x^{-(\alpha -1)}n^{-1}$. Moreover for some slowly varying function $\tilde{L}$ we have that $a_n=n^{\frac{1}{\alpha -1}}\tilde{L}(n)$.

In this case we have that the slowing is caused by the number of excursions in traps. Since $\beta$ is small (i.e. less than $\mu ^{-1}$) we have that the expected time spent in a trap is finite. The number of excursions the walk takes into a branch is of the same order as the number of buds; since the size-biased law has infinite mean there are a large number of buds and therefore a large number of excursions. The main result for IVFE is Theorem 1 which reflects that $\Delta _n$ scales similarly to the sum of independent copies of $\xi ^*$.

### Theorem 1

For IVFE, the laws of the process

$$\begin{aligned} \left( \frac{\Delta _{nt}}{a_n}\right) _{t\ge 0} \end{aligned}$$

converge weakly as $n \rightarrow \infty$ under $\mathbb {P}$ with respect to the Skorohod $J_1$ topology on $D([0,\infty ),\mathbb {R})$ to the law of an $\alpha -1$ stable subordinator $R_t$ with Laplace transform

$$\begin{aligned} \varphi _t(s):=\mathbb {E}\left[ e^{-sR_t}\right] =e^{-C_{\alpha ,\beta ,\mu }ts^{\alpha -1}} \end{aligned}$$

where $C_{\alpha ,\beta ,\mu }$ is a constant which we shall determine during the proof (see 9.1).

We refer to the second $(\sigma ^2<\infty , \;\beta \mu >1)$ and third $(\sigma ^2=\infty , \;\beta \mu >1)$ cases as the finite variance, infinite excursion and infinite variance, infinite excursion cases respectively.

### Definition 2

(*FVIE*) The offspring distribution has mean $\mu$ satisfying $1<\mu ^{-1}<\beta$ and variance $\sigma ^2<\infty$.

### Definition 3

(*IVIE*) The offspring distribution has mean $\mu$ satisfying $1<\mu ^{-1}<\beta$ and belongs to the domain of attraction of a stable law of index $\alpha \in (1,2)$.

As for IVFE, in IVIE we let *L* vary slowly at $\infty$ such that (2.2) holds and $(a_n)_{n\ge 1}$ be some scaling sequence for the size-biased law such that for any $x>0$, as $n \rightarrow \infty$ we have $\mathbf {P}(\xi ^*\ge xa_n) \sim x^{-(\alpha -1)}n^{-1}$. It then follows that $a_n=n^{\frac{1}{\alpha -1}}\tilde{L}(n)$ for some slowly varying function $\tilde{L}$. In FVIE, $a_n=n$ will suffice.

In FVIE and IVIE the slowing is caused by excursions in deep traps because the walk is required to make long sequences of movements against the bias in order to escape. We shall see that only the depth *H* (and not the foliage) is important to the scaling. By comparison with the model in which we strip all of the branch except the unique self-avoiding path to the deepest point; we see that, by transience, the walk reaches the deepest point with positive probability and then takes a geometric number of short excursions with escape probability of the order $\beta ^{-H}$. In particular, this means that the expected time spent in a branch of height *H* will cluster around $\beta ^{H}$.

Intuitively, the main reason we observe different scalings in these two cases is due to the way the number of buds affects the height of the branch. The height of a GW-tree is approximately geometric; in particular, the tallest of *n* independent trees will typically be close to $\log (n)/\log (\mu ^{-1})$. In FVIE the number of buds has finite mean therefore we see order *n* buds by level *n* hence tallest will have height close to $\log (n)/\log (\mu ^{-1})$. In IVIE the number of buds has infinite mean but belongs to the domain of attraction of some stable law. In particular, the number of buds seen by level *n* is equal in distribution to the sum of *n* independent copies of $\xi ^*-1$ (which scales with $a_n$). It therefore follows that, in IVIE, the tallest tree up to level *n* will have height close to $\log (a_n)/\log (\mu ^{-1})$. Since only the deepest trees are significant and the time spent in a large branch clusters around $\beta ^H$ we see that the natural scaling is $\beta ^{\log (n)/\log (\mu ^{-1})}=n^{1/\gamma }$ in FVIE and $\beta ^{\log (a_n)/\log (\mu ^{-1})}=a_n^{1/\gamma }$ in IVIE.

Since *H* is approximately geometric we have that $\beta ^H$ won’t belong to the domain of attraction of any stable law. For this reason, as in [4], we only see convergence along specific increasing subsequences $n_l(t):=\lfloor t \mu ^{-l}\rfloor$ for $t>0$ in FVIE and $n_l(t)$ such that $a_{n_l(t)} \sim t\mu ^{-l}$ for IVIE. Such a sequence exists for any $t>0$ since by choosing $n_l(t):=\sup \{m\ge 0:a_m< t\mu ^{-l}\}$ we have that $a_{n_l}< t\mu ^{-l} \le a_{n_l+1}$ and therefore

$$\begin{aligned} 1 \ge \frac{a_{n_l}}{t\mu ^{-l}} \ge \frac{a_{n_l}}{a_{n_l+1}} \rightarrow 1. \end{aligned}$$

Recalling (1.1), the main results for FVIE and IVIE are Theorems 2 and 3, which reflect slowing due to deep excursions.

### Theorem 2

In FVIE, for any $t>0$ we have that as $l\rightarrow \infty$

$$\begin{aligned} \frac{\Delta _{n_l(t)}}{n_l(t)^\frac{1}{\gamma }} \rightarrow R_t \end{aligned}$$

in distribution under $\mathbb {P}$, where $R_t$ is a random variable with an infinitely divisible law.

### Theorem 3

In IVIE, for any $t>0$ we have that as $l\rightarrow \infty$

$$\begin{aligned} \frac{\Delta _{n_l(t)}}{a_{n_l(t)}^\frac{1}{\gamma }} \rightarrow R_t \end{aligned}$$

in distribution under $\mathbb {P}$, where $R_t$ is a random variable with an infinitely divisible law.

We write $r_n$ to be $a_n$ in IVFE, $n^{1/\gamma }$ in FVIE and $a_n^{1/\gamma }$ in IVIE; then, letting $\overline{r}_n:=\max \{m\ge 0:r_m\le n\}$ we will also prove Theorem 4. This shows that, although the laws of $X_n/\overline{r}_n$ don’t converge in general (for FVIE and IVIE), the suitably scaled sequence is tight and we can determine the leading order polynomial exponent explicitly.

### Theorem 4

In IVFE, FVIE or IVIE we have that

1. The laws of $(\Delta _n/r_n)_{n\ge 0}$ under $\mathbb {P}$ are tight on $(0,\infty )$;
2. The laws of $(|X_n|/\overline{r}_n)_{n\ge 0}$ under $\mathbb {P}$ are tight on $(0,\infty )$.

Moreover, in IVFE, FVIE and IVIE respectively, we have that $\mathbb {P}$-a.s.

$$\begin{aligned} \lim _{n \rightarrow \infty }\frac{\log \left| X_n\right| }{\log (n)}= & {} \alpha -1; \\ \lim _{n \rightarrow \infty }\frac{\log \left| X_n\right| }{\log (n)}= & {} \gamma ; \\ \lim _{n \rightarrow \infty }\frac{\log \left| X_n\right| }{\log (n)}= & {} \gamma (\alpha -1). \end{aligned}$$

The final case we consider is an extension of a result of [4] for the walk on the supercritical tree. The argument used for the infinite variance case is generally the same as in the finite variance case but needs some technical input. This is provided by three lemmas which we put aside until Sect. 10. For the same reason as in FVIE, we only see convergence along specific subsequences $n_l(t):=\lfloor t f'(q)^{-l}\rfloor$ for $t>0$.

### Theorem 5

(Infinite variance supercritical case) Suppose the offspring law belongs to the domain of attraction of some stable law of index $\alpha \in (1,2)$, has mean $\mu >1$ and the bias satisfies the bound $\beta >f'(q)^{-1}$. Then,

$$\begin{aligned} \frac{\Delta _{n_l(t)}}{n_l(t)^\frac{1}{\gamma }} \rightarrow R_t \end{aligned}$$

in distribution as $l\rightarrow \infty$ under $\mathbb {P}$, where $R_t$ is a random variable with an infinitely divisible law whose parameters are given in [4]. Moreover, the laws of $(\Delta _nn^{-\frac{1}{\gamma }})_{n\ge 0}$ and $(|X_n|n^{-\gamma })_{n\ge 0}$ under $\mathbb {P}$ are tight on $(0,\infty )$ and $\mathbb {P}$-a.s.

$$\begin{aligned} \lim _{n \rightarrow \infty }\frac{\log \left| X_n\right| }{\log (n)} =\gamma . \end{aligned}$$

The proofs of Theorems 1, 2 and 3 follow a similar structure to the corresponding proof of [4] which, for the walk on the supercritical tree, only considers the case in which the variance of the offspring distribution is finite. However, for the latter reason, the proofs of Theorems 1 and 3 become more technical in some places, specifically with regards to the number of traps in a large branch. The proof can be broken down into a sequence of stages which investigate different aspects of the walk and the tree. This is ideal for extending the result onto the supercritical tree because many of these behavioural properties will be very similar for the walk on the subcritical tree due to the similarity of the traps.

In all cases it will be important to decompose large branches. In Sect. 3 we show a decomposition of the number of deep traps in any deep branch. This is only important for FVIE and IVIE since the depth of the branch plays a key role in decomposing the time spent in large branches. In Sect. 4 we determine conditions for labelling a branch as large in each of the regimes so that large branches are sufficiently far apart so that, with high probability, the underlying walk won’t backtrack from one large branch to the previous one. In Sect. 5 we justify the choice of label by showing that time spent outside these large branches is negligible. From this we then have that $\Delta _n$ can be approximated by a sum of i.i.d. random variables whose distribution depends on *n*. In Sect. 6 we only consider IVFE and show that, under a suitable scaling, these variables converge in distribution which allows us to show the convergence of their sum. Similarly, in Sect. 7 we show that the random variables, suitably scaled, converge in distribution for FVIE and IVIE. We then show convergence of their sum in Sect. 8. In Sect. 9 we prove Theorem 4 which is standard following Theorems 1, 2 and 3. Finally, in Sect. 10, we prove three short lemmas which extend the main result of [4] to prove Theorem 5. We require a lot of notation much of which is very similar; a glossary follows Sect. 10 which includes most of the notation used repeatedly throughout.

## Number of traps

In this section we show asymptotics for the probability that the height of a branch is large and use it to determine the distribution over the number of large traps in a large branch. Unless stated otherwise we assume $\mu <1$.

**Fig. 2:**
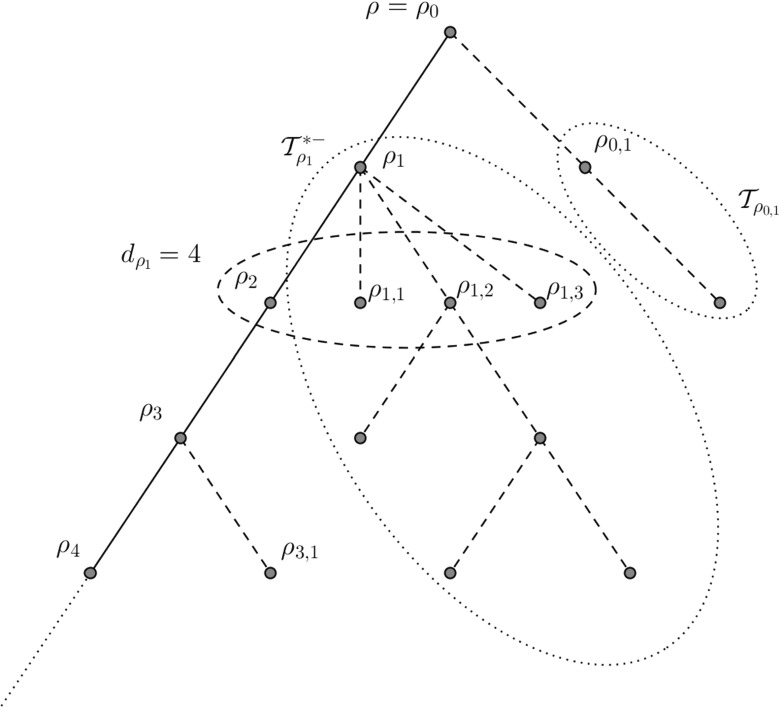
A sample section of a subcritical tree conditioned to survive with *solid lines* representing the backbone $\mathcal {Y}$ and *dashed lines* representing the dangling ends

In the construction of the subcritical GW-tree conditioned to survive $\mathcal {T}$ described in the introduction, the special vertices form the infinite backbone $\mathcal {Y}=\{\rho _0,\rho _1,\ldots \}$ consisting of all vertices with an infinite line of descent where $\rho _i$ is the vertex in generation *i*. Each vertex $\rho _i$ on the backbone is connected to buds $\rho _{i,j}$ for $j=1,\ldots ,d_{\rho _i}-1$ (which are the normal vertices that are offspring of special vertices in the construction). Each of these is then the root of an *f*-GW tree $\mathcal {T}_{\rho _{i,j}}$. We call each $\mathcal {T}_{\rho _{i,j}}$ a trap and the collection from a single backbone vertex (combined with the backbone vertex) $\mathcal {T}^{*-}_{\rho _i}$ a branch. Figure 2 shows an example of the first five generations of a tree $\mathcal {T}$. The solid line represents the backbone and the two dotted ellipses identify a sample branch and trap. The dashed ellipse indicates the children of $\rho _1$ which, since $\rho _1$ is on the backbone, have quantity distributed according to the size-biased law. It will be helpful throughout to work on a dummy branch which is equal in distribution to $\mathcal {T}^{*-}_{\rho _i}$ for any *i* thus we define the following random tree.

### Definition 4

(*Dummy branch*) Define $\mathcal {T}^{*-}$ to be a random tree rooted at $\rho$ with first generation vertices $\rho _1,\ldots ,\rho _{\xi ^*-1}$ which are roots of independent *f*-GW-trees $(\mathcal {T}_i^\circ )_{i= 1}^k$ where $\xi ^*$ is a size biased random variable independent of the rest of the tree. Define $\mathcal {T}^\circ$ to be a dummy *f*-GW-tree.

The structure of the large traps will have an important role in determining the convergence of the scaled process. In this section we determine the distribution over the number of deep traps rooted at backbone vertices with at least one deep trap. We will show that there is only a single deep trap at any backbone vertex when the offspring law has finite variance whereas, when the offspring law belongs to the domain of attraction of a stable law with index $\alpha <2$ we have that the number of deep traps converges in distribution to a certain heavy tailed law.

A fundamental result for branching processes (see, for example [20]), is that for $\mu <1$ and $Z_n$ an *f*-GW process, the sequence $\mathbf {P}(Z_n>0)/\mu ^n$ is decreasing; moreover, $\mathbf {E}[\xi \log (\xi )]<\infty$ if and only if the limit of $\mathbf {P}(Z_n>0)\mu ^{-n}$ as $n \rightarrow \infty$ exists and is strictly positive. This assumption holds under any of the hypotheses thus for this paper we will always make this assumption and let $c_\mu$ be the constant such that

3.1 $$\begin{aligned} \mathbf {P}(Z_n>0)\sim c_\mu \mu ^n. \end{aligned}$$

Recall that $\mathcal {H}(T)$ denotes the height of a tree *T* rooted at $\rho$. Denote

3.2 $$\begin{aligned} s_m:=\mathbf {P}\left( \mathcal {H}(\mathcal {T}^\circ )< m\right) =1-c_\mu \mu ^m(1+o(1)) \end{aligned}$$

to be the probability that a given trap is of height at most $m-1$ (although in general we shall write *s* for convenience). Write $N(m):=\sum _{j=1}^{\xi ^*-1}\mathbf {1}_{\{\mathcal {H}(\mathcal {T}_i^\circ )\ge m\}}$ to be the number of traps of height at least *m* in the dummy branch then we are interested in the limit as $m \rightarrow \infty$ of

3.3 $$\begin{aligned} \mathbf {P}\left( N(m)=l\big |N(m)\ge 1\right) =\frac{\mathbf {P}\left( N(m)=l\right) }{\mathbf {P}\left( N(m)\ge 1\right) } \end{aligned}$$

for $l \ge 1$. Recall that *f* is the p.g.f. of the offspring distribution, write $f^{(k)}$ for its $k{\text {th}}$ derivative then we have that

3.4 $$\begin{aligned} \mathbf {P}\left( N(m)=l\right)&= \sum _{k=1}^\infty \mathbf {P}\left( \xi ^*=k\right) \mathbf {P}\left( N(m)=l|\xi ^*=k\right) \nonumber \\&= \sum _{k=l+1}^\infty \frac{kp_k}{\mu }s^{k-1-l}(1-s)^l\left( {\begin{array}{c}k-1\\ l\end{array}}\right) \nonumber \\&= \frac{(1-s)^l}{l!\mu }f^{(l+1)}(s). \end{aligned}$$

In particular, we have that

3.5 $$\begin{aligned} \mathbf {P}\left( \mathcal {H}(\mathcal {T}^{*-})>m\right) =\mathbf {P}\left( N(m)\ge 1\right) = 1-f'(s)/\mu . \end{aligned}$$

Lemma 3.1 shows that, when $\sigma ^2<\infty$, with high probability there will only be a single deep trap in any deep branch.

### Lemma 3.1

When $$\sigma ^2<\infty $$

$$\begin{aligned} \lim _{m\rightarrow \infty } \mathbf {P}\left( N(m)=1\big |N(m)\ge 1\right) =1. \end{aligned}$$

### Proof

Using (3.3) and (3.4) we have that

3.6 $$\begin{aligned} \mathbf {P}\left( N(m)=1\big |N(m)\ge 1\right) \; = \; \frac{(1-s)f''(s)/\mu }{1-f'(s)/\mu } \; = \; \frac{\sum _{k=2}^\infty k(k-1)p_ks^{k-2}}{\sum _{k=2}^\infty kp_k\frac{1-s^{k-1}}{1-s}}. \end{aligned}$$

By monotonicity in *s* we have that

$$\begin{aligned} \lim _{s\rightarrow 1^\circ }\sum _{k=2}^\infty k(k-1)p_ks^{k-2} = \sum _{k=2}^\infty k(k-1)p_k \end{aligned}$$

which is finite since $\sigma ^2<\infty$. Each summand in the denominator is increasing in *s* for $s\in (0,1)$ and by L’Hopital’s rule $1-s^{k-1} \sim (k-1)(1-s)$ as $s\rightarrow 1^\circ$ therefore, by monotone convergence, the denominator in the final term of (3.6) converges to the same limit. $\square$

In order to determine the correct threshold for labelling a branch as large we will need to know the asymptotic form of $\mathbf {P}(N(m)\ge 1)$. Corollary 3.2 gives this for the finite variance case.

### Corollary 3.2

Suppose $$\sigma ^2<\infty $$ then

$$\begin{aligned} \mathbf {P}\left( N(m)\ge 1\right) \; \sim \; c_\mu \mathbf {E}\left[ \xi ^*-1\right] \mu ^m \; = \; c_\mu \left( \frac{\sigma ^2+\mu ^2}{\mu }-1\right) \mu ^m. \end{aligned}$$

### Proof

Let $f_*$ denote the p.g.f. of $\xi ^*$ then $\mathbf {P}(N(m)\ge 1) = 1-s^{-1}f_*(s)$. Since $\sigma ^2<\infty$ we have that $f_*'(s)$ exists and is continuous for $s\le 1$ thus as $s \rightarrow 1^\circ$ we have that $f_*(1)-f_*(s) \sim (1-s)f_*'(1) = (1-s)\mathbf {E}[\xi ^*]$. It therefore follows that

$$\begin{aligned} 1-s^{-1}f_*(s) \; = \; f_*(1)-f_*(s) -\frac{f_*(s)(1-s)}{s} \; \sim \; (1-s)\left( \mathbf {E}\left[ \xi ^*\right] -1\right) . \end{aligned}$$

The result then follows by the definitions of $c_\mu$ (3.1) and *s* (3.2). $\square$

We now consider the case when $\sigma ^2=\infty$ but $\xi$ belongs to the domain of attraction of a stable law of index $\alpha \in (1,2)$. The following lemma concerning the form of the probability generating function of the offspring distribution will be fundamental in determining the distribution over the number of large traps rooted at a given backbone vertex. The case $\mu =1$ appears in [7]; the proof of Lemma 3.3 is a simple extension of this hence the proof is omitted.

### Lemma 3.3

Suppose the offspring distribution belongs to the domain of attraction of a stable law with index $\alpha \in (1,2)$ and mean $\mathbf {E}[\xi ]=\mu$.

1. If $\mu \le 1$ then as $s\rightarrow 1^\circ$
  $$\begin{aligned} \mathbf {E}\left[ s^\xi \right] -s^\mu \sim \frac{\Gamma (3-\alpha )}{\alpha (\alpha -1)}(1-s)^\alpha L((1-s)^{-1}) \end{aligned}$$
  where $\Gamma (t)=\int _0^\infty x^{t-1}e^{-x}\mathrm {d}x$ is the usual gamma function.
2. If $$\mu >1$$ then
  $$\begin{aligned} 1-\mathbf {E}\left[ s^\xi \right] = \mu (1-s)+ \frac{\Gamma (3-\alpha )}{\alpha (\alpha -1)}(1-s)^\alpha \overline{L}\left( (1-s)^{-1}\right) \end{aligned}$$
  where $\overline{L}$ varies slowly at $\infty$.

When $\mu <1$ it follows that there exists a function $L_1$ (which varies slowly as $s\rightarrow 1^\circ$) such that $\mathbf {E}[s^\xi ]-s^\mu = (1-s)^\alpha L_1((1-s)^{-1})$ and

3.7 $$\begin{aligned} \lim _{s\rightarrow 1^\circ }\frac{L_1\left( (1-s)^{-1}\right) }{L\left( (1-s)^{-1}\right) }= \frac{\Gamma (3-\alpha )}{\alpha (\alpha -1)}. \end{aligned}$$

Write $g(x)=x^\alpha L_1(x^{-1})$ so that $f(s)=s^\mu +g(1-s)$ then it follows that

$$\begin{aligned} f^{(l)}(s)=s^{\mu -l}(\mu )_l+(-1)^lg^{(l)}(1-s) \end{aligned}$$

when this exists where $(\mu )_l:=\prod _{j=0}^{l-1}(\mu -j)$ is the Pochhammer symbol. Write $L_2(x):=L_1(x^{-1})$ which is slowly varying at 0. Using Theorem 2 of [19], we see that $xg'(x)\sim \alpha g(x)$ as $x \rightarrow 0$. Moreover, using an inductive argument in the proof of this result, it is straightforward to show that for all $l \in \mathbb {N}$ we have that $xg^{(l+1)}(x) \sim (\alpha -l)g^{(l)}(x)$ as $x\rightarrow 0$. Therefore, for any integer $l\ge 0$

3.8 $$\begin{aligned} \lim _{x \rightarrow 0^+}\frac{x^lg^{(l)}(x)}{g(x)} \; = \; (\alpha )_l . \end{aligned}$$

Proposition 3.4 is the main result of this section and determines the limiting distribution of the number of traps of height at least *m* in a branch of height greater than *m*.

### Proposition 3.4

In IVIE, for $l\ge 1$ as $m \rightarrow \infty$

$$\begin{aligned} \mathbf {P}\left( N(m)=l|N(m)\ge 1\right) \rightarrow \frac{1}{l!}\prod _{j=1}^l\left| \alpha -j\right| . \end{aligned}$$

### Proof

Recall that by (3.3) and (3.4) we want to determine the asymptotics of $1-f'(s)/\mu$ and $(1-s)^lf^{(l+1)}(s)/(l!\mu )$ as $s \rightarrow 1^\circ$. We have that $1-f'(s)/\mu =1-s^{\mu -1}+g'(1-s)/\mu$ and $g'(1-s) \sim \alpha (1-s)^{\alpha -1}L_2(1-s)$ as $s\rightarrow 1$. Since $\alpha <2$, we have that $\lim _{s \rightarrow 1^\circ }(1-s^{\mu -1})(1-s)^{1-\alpha } =0$ hence

3.9 $$\begin{aligned} 1-\frac{f'(s)}{\mu } \sim \frac{\alpha }{\mu }(1-s)^{\alpha -1}L_2(1-s). \end{aligned}$$

For derivatives $l \ge 1$ we have that

$$\begin{aligned} \frac{(1-s)^lf^{(l+1)}(s)}{l!\mu }= \frac{(1-s)^l}{l!\mu }\left( s^{\mu -(l+1)}(\mu )_l+(-1)^{l+1} g^{(l+1)}(1-s)\right) . \end{aligned}$$

By (3.8) we have that $(1-s)^lg^{(l+1)}(1-s)\sim (\alpha )_{l+1}(1-s)^{\alpha -1}L_2(1-s)$. For $l\ge 1$ we have that $l+1-\alpha >0$ hence

3.10 $$\begin{aligned} \frac{(1-s)^lf^{(l+1)}(s)}{l!\mu }&\sim \frac{\left| (\alpha )_{l+1}\right| }{l!\mu }(1-s)^{\alpha -1}L_2(1-s). \end{aligned}$$

Combining (3.3) with (3.9) and (3.10) gives the desired result. $\square$

Proposition 3.4 will be useful for determining the number of large traps in a large branch but equally important is the asymptotic relation (3.9) which gives the tail behaviour of the height of a branch $\mathcal {T}^{*-}$. By the assumption on $\xi$ that (2.2) holds we have that

3.11 $$\begin{aligned} \mathbf {P}\left( \xi ^*\ge t\right) \sim \frac{2-\alpha }{\mu (\alpha -1)}t^{-(\alpha -1)}L(t) \end{aligned}$$

as $t\rightarrow \infty$. Using (3.2), (3.5), (3.7), (3.9) and (3.11), we then have that

3.12 $$\begin{aligned} \mathbf {P}\left( \mathcal {H}(\mathcal {T}^{*-})>m\right) \; \sim \; \frac{\Gamma (3-\alpha )c_\mu ^{\alpha -1}}{\mu (\alpha -1)} \mu ^{m(\alpha -1)}L(\mu ^{-m}) \; \sim \; \Gamma (2-\alpha )c_\mu ^{\alpha -1}\mathbf {P}\left( \xi ^* \ge \mu ^{-m}\right) . \end{aligned}$$

## Large branches are far apart

In this section we introduce the conditions for a branch to be large. This will differ in each of the cases however, since many of the proofs will generalise to all three cases, we will use the same notation for some aspects.

In IVFE we will have that the slowing is caused by the large number of traps. In particular, we will be able to show that the time spent outside branches with a large number of buds is negligible.

### Definition 5

(*IVFE large branch*) For $\varepsilon \in (0,1)$ write

$$\begin{aligned} l_{n,\varepsilon } := a_{\lfloor n^{1-\varepsilon }\rfloor } \quad \text {and} \quad l_{n,\varepsilon }^+ := a_{\lfloor n^{1+\varepsilon }\rfloor } \end{aligned}$$

then we have that $\mathbf {P}(\xi ^*\ge l_{n,\varepsilon } ) \sim n^{-(1-\varepsilon )}$. We will call a branch large if the number of buds is at least $l_{n,\varepsilon }$ and write $\mathcal {D}^{(n)}:=\{x \in \mathcal {Y}:d_x>l_{n,\varepsilon }\}$ to be the collection of backbone vertices which are the roots of large branches.

In FVIE we will have that the slowing is caused by excursions into deep traps.

### Definition 6

(*FVIE large branch*) For $\varepsilon \in (0,1)$ write $C_\mathcal {D}:=c_\mu \mathbf {E}[\xi ^*-1]$,

$$\begin{aligned} h_{n,\varepsilon } :=\left\lceil \frac{(1-\varepsilon )\log (n)}{\log \left( \mu ^{-1}\right) }\right\rceil \quad \text {and} \quad h_{n,\varepsilon }^+ :=\left\lceil \frac{(1+\varepsilon )\log (n)}{\log \left( \mu ^{-1}\right) }\right\rceil \end{aligned}$$

then by Corollary 3.2 we have that

$$\begin{aligned} \mathbf {P}\left( \mathcal {H}\left( \mathcal {T}^{*-}\right) > h_{n,\varepsilon }\right) \; \sim \; C_\mathcal {D}\mu ^{h_{n,\varepsilon }} \; \approx \; C_\mathcal {D}n^{-(1-\varepsilon )}. \end{aligned}$$

We will call a branch large if there exists a trap within it of height at least $h_{n,\varepsilon }$ and write $\mathcal {D}^{(n)}:=\{x \in \mathcal {Y}:\mathcal {H}(\mathcal {T}_x^{*-})> h_{n,\varepsilon }\}$ to be the collection of backbone vertices which are the roots of large branches. By a large trap we mean any trap of height at least $h_{n,\varepsilon }$.

In IVIE we will have that the slowing is caused by a combination of the slowing effects of the other two cases. The height and number of buds in branches have a strong link which we show more precisely later; this allows us to label branches as large based on height which will be necessary when decomposing the time spent in large branches.

### Definition 7

(*IVIE large branch*) For $\varepsilon \in (0,1)$ write

$$\begin{aligned} h_{n,\varepsilon } :=\left\lceil \frac{\log \left( a_{n^{1-\varepsilon }}\right) }{\log \left( \mu ^{-1}\right) }\right\rceil \quad \text {and} \quad h_{n,\varepsilon }^+ :=\left\lceil \frac{\log \left( a_{n^{1+\varepsilon }}\right) }{\log \left( \mu ^{-1}\right) }\right\rceil \end{aligned}$$

then by (3.12), for $C_\mathcal {D}:=\Gamma (2-\alpha )c_\mu ^{\alpha -1}$, we have that

4.1 $$\begin{aligned} \mathbf {P}\left( \mathcal {H}\left( \mathcal {T}^{*-}\right) > h_{n,\varepsilon }\right) \; \sim \; C_\mathcal {D}\mathbf {P}\left( \xi ^*\ge \mu ^{-h_{n,\varepsilon }}\right) \; \approx \; C_\mathcal {D}n^{-(1-\varepsilon )}. \end{aligned}$$

We will call a branch large if there exists a trap of height at least $h_{n,\varepsilon }$ and write $\mathcal {D}^{(n)}:=\{x \in \mathcal {Y}:\mathcal {H}(\mathcal {T}_x^{*-})> h_{n,\varepsilon }\}$ to be the collection of backbone vertices which are the roots of large branches. By a large trap we mean any trap of height at least $h_{n,\varepsilon }$.

We want to show that, asymptotically, the large branches are sufficiently far apart to ignore any correlation and therefore approximate $\Delta _n$ by the sum of i.i.d. random variables representing the time spent in a large branch. Much of this is very similar to [4] so we only give brief details.

Write $\mathcal {D}_m^{(n)}:=\{x \in \mathcal {D}^{(n)}:|x|\le m\}$ to be the large roots before level *m* then let $q_n:=\mathbf {P}(\rho \in \mathcal {D}^{(n)})$ be the probability that a branch is large and write

4.2 $$\begin{aligned} A_1(n,T):=\left\{ \sup _{t \in [0,T]}\left| \left| \mathcal {D}_{\left\lfloor tn\right\rfloor }^{(n)}\right| -\left\lfloor tnq_n\right\rfloor \right| < n^{2\varepsilon /3}\right\} \end{aligned}$$

to be the event that the number of large branches by level *Tn* doesn’t differ too much from its expected value. Notice that in all three cases we have that $q_n$ is of the order $n^{-(1-\varepsilon )}$ thus we expect to see $nq_n \approx Cn^\varepsilon$ large branches by level *n*.

### Lemma 4.1

For any $$T>0$$

$$\begin{aligned} \lim _{n \rightarrow \infty }\mathbf {P}\left( A_1(n,T)^c\right) =0. \end{aligned}$$

### Proof

For each $n \in \mathbb {N}$ write

$$\begin{aligned} M_m^n:=\left| \mathcal {D}_m^{(n)}\right| -mq_n \mathop {=}\limits ^{{\mathrm{d}}}\sum _{k=1}^m \left( B_k-q_n\right) \end{aligned}$$

where $B_k$ are independent Bernoulli random variables with success probability $q_n$. Then $\mathbf {E}[M_m^n]=0$ and $Var_{\mathbf {P}}(M_m^n)=mq_n(1-q_n)$ therefore by Kolmogorov’s maximal inequality

$$\begin{aligned} \mathbf {P}\left( \max _{1\le m\le \left\lfloor nT\right\rfloor }\left| M_m^n\right| \ge n^{2\varepsilon /3}-1\right) \;\le \; \frac{cVar_{\mathbf {P}}\left( M_{\lfloor nT\rfloor }^n\right) }{n^{4\varepsilon /3}} \;\le \; \frac{cnTq_n}{n^{4\varepsilon /3}}\;\le \; CTn^{-\varepsilon /3}. \end{aligned}$$

Since $\left| \lfloor nt\rfloor q_n-\lfloor ntq_n\rfloor \right| \le 1$ we have that

$$\begin{aligned} \sup _{t \in [0,T]}\left| \left| \mathcal {D}_{\lfloor tn\rfloor }^{(n)}\right| -\lfloor tnq_n\rfloor \right|\le & {} \max _{1\le m\le \left\lfloor nT\right\rfloor }\left| M_m^n\right| +\sup _{0\le t\le T} \left| tnq_n-\lfloor nt\rfloor q_n\right| \\\le & {} \max _{1\le m\le \left\lfloor nT\right\rfloor }\left| M_m^n\right| +1 \end{aligned}$$

which proves the statement. $\square$

We want to show that all of the large branches are sufficiently far apart such that the walk doesn’t backtrack from one to another. For $t>0$ and $\kappa \in (0,1-2\varepsilon )$ write

$$\begin{aligned} \mathcal {D}(n,t):=\left\{ \min _{\begin{array}{c} \scriptscriptstyle {x,y \in \mathcal {D}_{\scriptscriptstyle {\lfloor nt\rfloor }}^{(n)}}: \; \scriptscriptstyle {x\ne y} \end{array}}d(x,y)>n^{\kappa }\right\} \cap \left\{ \rho \notin \mathcal {D}^{(n)}\right\} \end{aligned}$$

to be the event that all large branches up to level $\lfloor nt\rfloor$ are of distance at least $n^\kappa$ apart and the root of the tree is not the root of a large branch. A union bound shows that $\mathbf {P}(\mathcal {D}(n,t)^c)\rightarrow 0$ as $n \rightarrow \infty$ uniformly over *t* in compact sets.

We want to show that, with high probability, once the walk reaches a large branch it never backtracks to the previous one. For $t>0$ write

$$\begin{aligned} A^{(0)}_2(n,t):=\bigcap _{i=0}^{\lfloor nt\rfloor }\bigcap _{m \ge \Delta ^Y_{\rho _i}}\left\{ \left| Y_m\right| >i-\overline{C}\log (n)\right\} \end{aligned}$$

to be the event that the walk never backtracks distance $\overline{C}\log (n)$ (where $\Delta _n^Y:=\min \{m\ge 0:Y_m=\rho _n\}$). For $x \in \mathcal {T}$ write $\tau _x^+=\inf \{n>0:X_n=x\}$ to be the first return time of *x*. Comparison with a simple random walk on $\mathbb {Z}$ shows that for $k\ge 1$ we have that the escape probability is $P _{\rho _k}\left( \tau _{\rho _{k-1}}^+<\infty \right) =\beta ^{-1}$ hence, using the Strong Markov property,

$$\begin{aligned} P _{\rho _m}\left( \tau _{\rho _0}^+<\infty \right) =\beta ^{-m}. \end{aligned}$$

Using a union bound we see that

4.3 $$\begin{aligned} \mathbb {P}\left( A^{(0)}_2(n,t)^c\right) \le Cnt\beta ^{-\overline{C}\log (n)} \rightarrow 0 \end{aligned}$$

for $\overline{C}$ sufficiently large. Combining this with $\mathcal {D}(n,t)$ we have that with high probability the walk never backtracks from one large branch to a previous one.

## Time is spent in large branches

In this section we show that the time spent up to time $\Delta _n$ outside large branches is negligible. Combined with Sect. 4 this allows us to approximate $\Delta _n$ by the sum of i.i.d. random variables. We begin with some general results concerning the number of excursions into traps and the expected time spent in a trap of height at most *m*.

Recall that $\rho _{i,j}$ are the buds connected to the backbone vertex $\rho _i$, that $c(\rho _i):=\{\rho _{i,j}\}_j \cup \{\rho _{i+1}\}$ is the collection of all offspring of $\rho _i$ and $d_{\rho _i}=|c(\rho _i)|$ is the number of offspring. We write $W^{i,j}:=|\{m \ge 0: X_{m-1}=\rho _i, \; X_m=\rho _{i,j}\}|$ to be the number of excursions into the $j{\text {th}}$ trap of the $i{\text {th}}$ branch where we set $W^{i,j}:=0$ if $\rho _{i,j}$ doesn’t exist in the tree. Lemma 5.1 shows that, conditional on the number of buds, the number of excursions follows a geometric law.

### Lemma 5.1

For any $i,k \in \mathbb {N}$ and $A \subset \{1,\ldots ,d_{\rho _i}-1\}$, when $\beta >1$

$$\begin{aligned} \sum _{j\in A}W^{i,j} \sim Geo\left( \frac{\beta -1}{(\left| A\right| +1)\beta -1}\right) \end{aligned}$$

with respect to $P ^{\mathcal {T}}$. In particular for any $j \le k$ we have that $W^{i,j}\sim Geo(p)$ where $p=(\beta -1)/(2\beta -1)$.

Moreover, under this law, $(W^{i,j})_{j \in A}$ have a negative multinomial distribution with one failure until termination and probabilities

$$\begin{aligned} q_j:={\left\{ \begin{array}{ll} \frac{\beta -1}{(\left| A\right| +1)\beta -1} &{} j=0 \\ \frac{\beta }{(\left| A\right| +1)\beta -1} &{} j\in A \\ \end{array}\right. } \end{aligned}$$

that from $\rho _i$ the next excursion will be into the $j{\text {th}}$ trap (where $j=0$ denotes escaping).

### Proof

From $\rho _{i,j}$ the walk must return to $\rho _i$ before escaping therefore since $P _{\rho _{i,j}}(\tau ^+_{\rho _i}<\infty )=1$, any traps not in the set we consider can be ignored and it suffices to assume that $A = \{1,\ldots ,k\}$. By comparison with a biased random walk on $\mathbb {Z}$ we have that $P _{\rho _{i+1}}(\tau ^+_{\rho _i}=\infty )=1-\beta ^{-1}.$ If $d_{\rho _i}=k+1$ then $P _{\rho _i}(\tau ^+_x=\min _{y \in c(\rho _i)}\tau ^+_y)=(k+1)^{-1}$ for any $x \in c(\rho _i)$. The probability of never entering a trap in the branch $\mathcal {T}^{*-}_{\rho _i}$ is, therefore,

$$\begin{aligned} P _{\rho _i}\left( \bigcap _{j=1}^k\left\{ \tau ^+_{\rho _{i,j}}=\infty \right\} \right) \; = \; \sum _{l=0}^\infty \left( \frac{1}{k+1}\beta ^{-1}\right) ^l\left( \frac{1-\beta ^{-1}}{k+1}\right) \; = \; \frac{\beta -1}{(k+1)\beta -1}. \end{aligned}$$

Each excursion ends with the walker at $\rho _i$ thus the walk takes a geometric number of excursions into traps with escape probability $(\beta -1)/((k+1)\beta -1)$. The second statement then follows from the fact that the walker has equal probability of going into any of the traps. $\square$

For a fixed tree *T* with $n{\text {th}}$ generation size $Z_n^T$ where $Z_1^T>0$ it is classical (e.g. [22]) that

5.1 $$\begin{aligned} E ^T_\rho \left[ \tau ^+_\rho \right] =2\sum _{n\ge 1}\frac{Z_n^T\beta ^{n-1}}{Z_1^T}. \end{aligned}$$

Let $\mathcal {T}^\leftarrow$ be an *f*-GW tree rooted at $\overleftarrow{\rho }$ conditioned to have a single first generation vertex which we label $\rho$. Notice that this has the same distribution as an *f*-GW-tree $\mathcal {T}^\circ$ to which we append a single ancestor of the root. From (5.1) it follows that

$$\begin{aligned} E ^{\mathcal {T}^\leftarrow }_{\rho }\left[ \tau ^+_{\overleftarrow{\rho }}\right] \; = \; E ^{\mathcal {T}^\leftarrow }_{\overleftarrow{\rho }}\left[ \tau ^+_{\overleftarrow{\rho }}\right] -1 \; = \; 2\sum _{n \ge 0} Z_n^{\mathcal {T}^\circ }\beta ^n-1. \end{aligned}$$

For any $m\ge 1$ we have that $\mathbf {P}(\mathcal {H}(\mathcal {T}^\leftarrow )\le m)\ge p_0$ therefore, for some constant *C*,

5.2 $$\begin{aligned} \mathbf {E}\left[ E ^{\mathcal {T}^\leftarrow }_{\rho }\left[ \tau ^+_{\overleftarrow{\rho }}\right] \big |\mathcal {H}(\mathcal {T}^\leftarrow )\le m \right] \le \frac{\mathbf {E}\left[ 2\sum _{n =0}^{m-1} Z_n^{\mathcal {T}^\circ }\beta ^n-1\right] }{\mathbf {P}\left( \mathcal {H}(\mathcal {T}^\leftarrow )\le m\right) } \; \le \; {\left\{ \begin{array}{ll} C(\mu \beta )^m &{} \beta \mu >1 \\ Cm &{} \beta \mu =1 \\ C &{} \beta \mu <1. \end{array}\right. } \end{aligned}$$

Recall that $\Delta ^Y_n$ is the first hitting time of $\rho _n$ for the underlying walk *Y* and write

$$\begin{aligned} A_3(n):= \left\{ \Delta ^Y_n\le C_1n\right\} \end{aligned}$$

to be the event that level *n* is reached by time $C_1n$ by the walk on the backbone. Standard large deviation estimates yield that $\lim _{n \rightarrow \infty }\mathbb {P}(A_3(n)^c)=0$ for $C_1>(\beta +1)/(\beta -1)$.

For the remainder of this section we mainly consider the case in which $\xi$ belongs to the domain of attraction of a stable law of index $\alpha \in (1,2)$. The case in which the offspring law has finite variance will proceed similarly however since the corresponding estimates are much simpler in this case we omit the proofs.

In IVIE and IVFE, for $t>0$, let the event that there are at most $\log (n)a_n$ buds by level $\lfloor nt\rfloor$ be

$$\begin{aligned} A_4(n,t):=\left\{ \sum _{k=0}^{\lfloor nt\rfloor }(d_{\rho _k}-1) \le \log (n)a_n \right\} . \end{aligned}$$

The variables $d_{\rho _k}$ are i.i.d. with the law of $\xi ^*$ therefore the laws of $a_{\lfloor nt\rfloor }^{-1}\sum _{k=1}^{\lfloor nt\rfloor }(\xi ^*_k-1)$ converge to some stable law $G^*$ where $\lim _{n \rightarrow \infty }\overline{G}^*(Ct^{\alpha -1}\log (n))=0$ uniformly over $t\le T$ therefore we have that $\lim _{n \rightarrow \infty }\mathbf {P}(A_4(n,t)^c)=0$.

In FVIE write

$$\begin{aligned} A_4(n,t):=\left\{ \sum _{k=0}^{\lfloor nt\rfloor }(d_{\rho _k}-1) \le \log (n)n \right\} \end{aligned}$$

then Markov’s inequality gives that $\lim _{n \rightarrow \infty }\mathbf {P}(A_4(n,t)^c)=0$.

Write

5.3 $$\begin{aligned} A_5(n):=\left\{ \max _{i,j}\left| \left\{ k \le \Delta _{\lfloor nt\rfloor }:X_{k-1}=\rho _i, \; X_k =\rho _{i,j}\right\} \right| \le C_2\log (n)\right\} \end{aligned}$$

to be the event that any trap is entered at most $C_2\log (n)$ times. By Lemma 5.1 the number of entrances into $\rho _{i,j}$ has the law of a geometric random variable of parameter $p=(\beta -1)/(2\beta -1)$ hence using a union bound we have that

$$\begin{aligned} \mathbb {P}\left( A_5(n,t)^c \cap A_4(n,t)\right) \le \log (n)a_n\mathbb {P}\left( Geo(p)>C_2\log (n)\right) \le \overline{L}(n)n^{\frac{1}{\alpha -1}+C_2\log \left( 1-p\right) } \end{aligned}$$

where $\overline{L}$ varies slowly hence the final term converges to 0 for $C_2$ large and $\lim _{n \rightarrow \infty }\mathbb {P}(A_5(n,t)^c)=0$.

Propositions 5.2, 5.3 and 5.4 show that any time spent outside large traps is negligible. In FVIE and IVIE we only consider the large traps in large branches. Recall that $\mathcal {D}^{(n)}$ is the set of roots of large branches and write

$$\begin{aligned} K(n):=\bigcup _{x \in \mathcal {D}^{(n)}}\left\{ \mathcal {T}_y:y \in c(x){\setminus } \left\{ \rho _{|x|+1}\right\} ,\;\mathcal {H}(\mathcal {T}_y)\ge h_{n,\varepsilon }\right\} \end{aligned}$$

to be the vertices in large traps. In IVFE we require the entire large branch and write

$$\begin{aligned} K(n):=\bigcup _{x \in \mathcal {D}^{(n)}}\{y \in \mathcal {T}^{*-}_x\} \end{aligned}$$

to be the vertices in large branches. In either case we write $\chi _{t,n}:=|\{1\le i\le \Delta _{\lfloor nt\rfloor }: \;X_{i-1},X_i \in K(n)\}|$ to be the time spent up to $\Delta _{\lfloor nt \rfloor }$ in large traps.

### Proposition 5.2

In IVIE, for any $t,\epsilon >0$ we have that as $n \rightarrow \infty$

$$\begin{aligned} \mathbb {P}\left( \left| \frac{\Delta _{\lfloor nt\rfloor }-\chi _{t,n}}{a_n^{1/\gamma }}\right| \ge \epsilon \right) \rightarrow 0. \end{aligned}$$

### Proof

On $A_4(n,t)$ there are at most $a_n\log (n)$ traps by level $\lfloor nt\rfloor$. We can order these traps so write $T^{(l,k)}$ to be the duration of the $k{\text {th}}$ excursion into the $l{\text {th}}$ trap and $\rho (l)$ to be the root of this trap (that is, the unique bud in the trap). Here we consider an excursion to start from the bud and end at the last hitting time of the bud before returning to the backbone. Recall that on $A_3(n)$ the walk *Y* reaches level *n* by time $C_1n$ and on $A_5(n)$ no trap up to level *n* is entered more than $C_2\log (n)$ times. Using the estimates on $A_3,A_4$ and $A_5$ we have that

$$\begin{aligned}&\mathbb {P}\left( \left| \frac{\Delta _{\lfloor nt\rfloor }-\chi _{t,n}}{a_n^{1/\gamma }}\right| \ge \epsilon \right) \le o(1)\\&\quad +\mathbb {P}\left( C_1nt+\sum _{l=0}^{a_n\log (n)} \sum _{k=1}^{C_2\log (nt)}\left( 2+T^{(l,k)}\mathbf {1}_{\left\{ \mathcal {H}(\mathcal {T}_{\rho (l)})< h_{n,\varepsilon }\right\} }\right) \ge \epsilon a_n^{\frac{1}{\gamma }}\right) . \end{aligned}$$

Since $a_n^{\frac{1}{\gamma }}\gg a_n\log (n)^2 \gg n$, for *n* sufficiently large we have that, using Markov’s inequality and (5.2) with $m= h_{n,\varepsilon }$, the second term can be bounded above by

$$\begin{aligned} 2\epsilon ^{-1}a_n^{-\frac{1}{\gamma }}\mathbb {E}\left[ \sum _{l=0}^{a_n\log (n)}\sum _{k=1}^{C_2\log (nt)} T^{(l,k)}\mathbf {1}_{\left\{ \mathcal {H}\left( \mathcal {T}_{\rho (l)}\right) < h_{n,\varepsilon }\right\} }\right] \; \le \; C_{t,\epsilon }\log (n)^2a_n^{1-\frac{1}{\gamma }} a_{n^{1-\varepsilon }}^{\frac{1}{\gamma }-1}. \end{aligned}$$

Combining constants and slowly varying functions into a single function $L_{t,\epsilon }$ such that for any $\tilde{\varepsilon }>0$ we have that $L_{t,\epsilon }(n)\le n^{\tilde{\varepsilon }}$ for *n* sufficiently large we then have that

$$\begin{aligned} \mathbb {P}\left( \left| \frac{\Delta _{\lfloor nt\rfloor }-\chi _{t,n}}{a_n^{1/\gamma }}\right| \ge \epsilon \right) \le o(1) + L_{t,\epsilon }(n)n^{-\varepsilon \frac{\frac{1}{\gamma }-1}{\alpha -1}} \end{aligned}$$

which converges to 0 since $\alpha ,\frac{1}{\gamma }>1$. $\square$

Using $A_3,A_5$ and the form of $A_4$ for FVIE, the technique used to prove Proposition 5.2 extends straightforwardly to prove Proposition 5.3 therefore we omit the proof.

### Proposition 5.3

In FVIE, for any $t, \epsilon >0$ we have that as $n \rightarrow \infty$

$$\begin{aligned} \mathbb {P}\left( \left| \frac{\Delta _{\lfloor nt\rfloor }-\chi _{t,n}}{n^{1/\gamma }}\right| \ge \epsilon \right) \rightarrow 0. \end{aligned}$$

Similarly, we can show a corresponding result for IVFE.

### Proposition 5.4

In IVFE, for any $t, \epsilon >0$, as $n \rightarrow \infty$

$$\begin{aligned} \mathbb {P}\left( \left| \frac{\Delta _{\lfloor nt\rfloor }-\chi _{t,n}}{a_n}\right| \ge \epsilon \right) \rightarrow 0. \end{aligned}$$

### Proof

We begin by bounding the total number of traps in small branches. Recall from Definition 5 that $l_{n,\varepsilon }\le a_{n^{1-\varepsilon }}$. Let $c \in (0,2-\alpha )$ then, by Markov’s inequality and the truncated first moment asymptotic:

5.4 $$\begin{aligned} \mathbf {E}\left[ \xi ^*\mathbf {1}_{\{\xi ^*\le x\}}\right] \sim C x^{2-\alpha }L(x) \end{aligned}$$

as $x\rightarrow \infty$ for some constant *C* (see for example [12], IX.8), for *n* large

$$\begin{aligned} \mathbb {P}\left( \sum _{k=0}^{\lfloor nt \rfloor }(d_{\rho _k}-1)\mathbf {1}_{\left\{ d_{\rho _k}-1\le l_{n,\varepsilon }\right\} } \ge n^{\frac{1-c\varepsilon }{\alpha -1}}\right)&\le \; \frac{\mathbf {E}\left[ \sum _{k=0}^{\lfloor nt \rfloor }(d_{\rho _k}-1)\mathbf {1}_{\left\{ d_{\rho _k}-1\le l_{n,\varepsilon }\right\} } \right] }{n^{\frac{1-c\varepsilon }{\alpha -1}}}\\&\le \; n^{-\frac{\varepsilon (2-\alpha +c)}{\alpha -1}}L_t(n) \end{aligned}$$

where $L_t(n)$ varies slowly at $\infty$. This converges to 0 as $n \rightarrow \infty$. We can order the traps in small branches and write $T^{(l,k)}$ to be the duration of the $k{\text {th}}$ excursion in the $l{\text {th}}$ trap not in a large branch where we consider an excursion to start and end at the backbone. Using $A_3$ and $A_5$ to bound the time taken by *Y* to reach level *nt* and the number of entrances into traps up to level *nt* we have that for *n* suitably large

$$\begin{aligned} \mathbb {P}\left( \left| \frac{\Delta _{\lfloor nt\rfloor }-\chi _{t,n}}{a_n}\right| \ge \epsilon \right)&\le o(1) + \mathbb {P}\left( \sum _{l=0}^{n^{\frac{1-c\varepsilon }{\alpha -1}}}\sum _{k=0}^{C_2\log (nt)}T^{(l,k)} \ge \frac{\epsilon }{2}a_n\right) . \end{aligned}$$

Using Markov’s inequality on the final term yields

$$\begin{aligned} \mathbb {P}\left( \sum _{l=0}^{n^{\frac{1-c\varepsilon }{\alpha -1}}}\sum _{k=0}^{C_2\log (nt)}T^{(l,k)} \ge \frac{\epsilon }{2}a_n\right)&\le 2\epsilon ^{-1}a_n^{-1}\mathbb {E}\left[ \sum _{k=0}^{n^{\frac{1-c\varepsilon }{\alpha -1}}} \sum _{j=0}^{C_2\log (nt)}T^{(l,k)}\right] \\&\le n^{\frac{-c\varepsilon }{\alpha -1}} L_{t,\epsilon }(n) \end{aligned}$$

for some $L_{t,\epsilon }$ varying slowly at $\infty$. This converges to 0 as $n \rightarrow \infty$ hence the result holds. $\square$

Recall that we write $r_n$ to be $a_n$ in IVFE, $n^{1/\gamma }$ in FVIE and $a_n^{1/\gamma }$ in IVIE. Since $\Delta _{\lfloor nt \rfloor }-\chi _{t,n}$ is non-negative and non-decreasing in *t* we have that $\sup _{0\le t\le T}|\Delta _{\lfloor nt \rfloor }-\chi _{t,n}|=|\Delta _{\lfloor nT \rfloor }-\chi _{T,n}|$ therefore Corollary 5.5 follows from Propositions 5.2, 5.3 and 5.4.

### Corollary 5.5

In each of IVFE, FVIE and IVIE, for any $T>0$

$$\begin{aligned} \sup _{0\le t\le T} \frac{\left| \Delta _{\lfloor nt \rfloor }-\chi _{t,n}\right| }{r_n} \end{aligned}$$

converges in $\mathbb {P}$-probability to 0.

Let $\Lambda$ be the set of strictly increasing continuous functions mapping [0, *T*] onto itself and *I* the identity map on [0, *T*] then we consider the Skorohod $J_1$ metric

$$\begin{aligned} d_{J_1}(f,g):=\inf _{\lambda \in \Lambda }\sup _{t \in [0,T]}\left( \left| f(t)-g(\lambda (t))\right| +\left| t-\lambda (t)\right| \right) . \end{aligned}$$

Write $\chi ^i_n$ to be the total time spent in large traps of the $i{\text {th}}$ large branch; that is

$$\begin{aligned} \chi ^i_n:=\left| \left\{ m\ge 0:X_{m-1},X_m \in \left( \mathcal {T}^{*-}_{\rho _i^+}\cap K(n)\right) \right\} \right| \end{aligned}$$

where $\rho _i^+$ is the element of $\mathcal {D}^{(n)}$ which is $i{\text {th}}$ closest to $\rho$. Notice that, whereas $\chi _{n,t}$ only accumulates time up to reaching $\rho _{\lfloor nt\rfloor }$, each $\chi _n^{i}$ may have contributions at arbitrarily large times. Recall that $A_2^{(0)}(n,t)$ is the event that the walk never backtracks distance $\overline{C}\log (n)$ along the backbone from a backbone vertex up to level $\lfloor nt\rfloor$. On $A_2^{(0)}(n,T)$ we therefore have that for all $t\le T$

$$\begin{aligned} \sum _{i=1}^{\left| \mathcal {D}_{\lfloor nt-\overline{C}\log (n)\rfloor }^{(n)}\right| }\chi ^i_n \; \le \; \chi _{n,t} \; \le \; \sum _{i=1}^{\left| \mathcal {D}_{\lfloor nt\rfloor }^{(n)}\right| }\chi ^i_n \end{aligned}$$

where, on $\mathcal {D}(n,t)$, the $J_1$ distance between the two sums in the above expression can be bounded above by $\overline{C}\log (n)$. In particular, using that $A_2^{(0)}(n,T)$ and $\mathcal {D}(n,t)$ occur with high probability with the tightness result we prove in Sect. 9, in order to prove Theorems 1, 2 and 3 it will suffice to consider the time spent in large traps up to level $\lfloor nt\rfloor$ under the appropriate scaling.

Let $(X_n^{(i)})_{i\ge 1}$ be independent walks on $\mathcal {T}$ with the law of $X_n$ and $(Y_n^{(i)})_{i\ge 1}$ the corresponding backbone walks. For $i\ge 1$ let $\tilde{\chi }_n^i$ be the time spent in the $i{\text {th}}$ large trap by $X_n^{(i)}$ and

$$\begin{aligned} \tilde{\chi }_{t,n}:=\sum _{i=1}^{\lfloor nt q_n\rfloor }\tilde{\chi }^i_n. \end{aligned}$$

The random variables $\tilde{\chi }_n^i$ are independent copies (under $\mathbb {P}$) of times spent in large branches; moreover, on $\mathcal {D}(n,t)$, $\rho \notin \mathcal {D}^{(n)}$ therefore they are identically distributed. Let $\mathbb {E}$ extend to the enlarged space.

### Lemma 5.6

In each of IVFE, FVIE and IVIE,

1. as $$n \rightarrow \infty $$
  $$\begin{aligned} d_{J_1}\left( \left( \sum _{i=1}^{\left| \mathcal {D}_{\lfloor nt\rfloor }^{(n)}\right| }\frac{\chi ^i_n}{r_n}\right) _{t \in [0,T]},\left( \sum _{i=1}^{\lfloor tnq_n \rfloor }\frac{\chi ^i_n}{r_n}\right) _{t \in [0,T]}\right) \end{aligned}$$
  converges to 0 in probability;
2. for any bounded $H:D([0,T],\mathbb {R})\rightarrow \mathbb {R}$ continuous with respect to the Skorohod $J_1$ topology, as $n \rightarrow \infty$
  $$\begin{aligned} \left| \mathbb {E}\left[ H\left( \left( \sum _{i=1}^{\lfloor tnq_n \rfloor }\frac{\chi ^i_n}{r_n}\right) _{t \in [0,T]}\right) \right] -\mathbb {E}\left[ H\left( \left( \sum _{i=1}^{\lfloor tnq_n \rfloor }\frac{\tilde{\chi }^i_n}{r_n}\right) _{t \in [0,T]}\right) \right] \right| \rightarrow 0. \end{aligned}$$

### Proof

By definition of $d_{J_1}$, the distance in statement 1 is equal to

$$\begin{aligned} \inf _{\lambda \in \Lambda }\sup _{t \in [0,T]} \left( \left| \sum _{i=1}^{\left| \mathcal {D}_{\lfloor nt\rfloor }^{(n)}\right| }\frac{\chi ^i_n}{r_n}-\sum _{i=1}^{\left\lfloor \lambda (t)nq_n \right\rfloor }\frac{\chi ^i_n}{r_n}\right| +\left| \lambda (t)-t\right| \right) . \end{aligned}$$

For $m \in \mathbb {N}$ let $\lambda _n(m/n):=|\mathcal {D}_{m}^{(n)}|(nq_n)^{-1}$ then define $\lambda _n(t)$ by the usual linear interpolation. It follows that $|\mathcal {D}_{\lfloor nt\rfloor }^{(n)}|=\lfloor \lambda _n(t)nq_n \rfloor$ and the above expression can be bounded above by

$$\begin{aligned} \sup _{t \in [0,T]}\left| t-\frac{\left| \mathcal {D}_{\lfloor nt\rfloor }^{(n)}\right| }{nq_n}\right| \end{aligned}$$

which converges to 0 by Lemma 4.1 since $n^{2\varepsilon /3}(nq_n)^{-1} \rightarrow 0$.

For $$i\ge 1$$ let

$$\begin{aligned} A_2^{(i)}(n,t):=\bigcap _{j=0}^{\lfloor nt\rfloor }\bigcap _{m \ge \Delta ^{Y^{(i)}}_{\rho _j}}\left\{ \left| Y_m^{(i)}\right| >j -\overline{C}\log (n)\right\} \end{aligned}$$

be the analogue of $A^{(0)}_2(n,t)$ for the $i{\text {th}}$ copy and

$$\begin{aligned} \tilde{A}_2(n,t):=\mathcal {D}(n,t)\cap \bigcap _{i=0}^{\lfloor ntq_n\rfloor }A_2^{(i)}(n,t) \end{aligned}$$

be the event that $\rho$ is not the root of a large branch, on each of the first $\lceil ntq_n\rceil$ copies the walk never backtracks distance $\overline{C}\log (n)$ and that large branches are of distance at least $n^\kappa$ apart.

$$\begin{aligned} \mathbb {E}\left[ H\left( \left( \sum _{i=1}^{\lfloor tnq_n \rfloor }\frac{\chi ^i_n}{r_n}\right) _{t \in [0,T]}\right) \mathbf {1}_{\tilde{A}_2(n,T)}\right] =\mathbb {E}\left[ H\left( \left( \sum _{i=1}^{\lfloor tnq_n \rfloor }\frac{\tilde{\chi }^i_n}{r_n}\right) _{t \in [0,T]}\right) \mathbf {1}_{\tilde{A}_2(n,T)}\right] \end{aligned}$$

therefore

$$\begin{aligned}&\left| \mathbb {E}\left[ H\left( \left( \sum _{i=1}^{\lfloor tnq_n \rfloor }\frac{\chi ^i_n}{r_n}\right) _{t \in [0,T]}\right) \right] -\mathbb {E}\left[ H\left( \left( \sum _{i=1}^{\lfloor tnq_n \rfloor }\frac{\tilde{\chi }^i_n}{r_n}\right) _{t \in [0,T]}\right) \right] \right| \\&\quad \le \left| \left| H\right| \right| _\infty \left( \lceil nTq_n\rceil \mathbb {P}\left( A^{(0)}_2(n,T)^c\right) +\mathbf {P}\left( \mathcal {D}(n,T)^c\right) \right) \end{aligned}$$

which converges to 0 as $n \rightarrow \infty$ for $\overline{C}$ large by the same argument as (4.3) and that $\mathbf {P}(\mathcal {D}(n,T)^c)\rightarrow 0$. $\square$

Using Corollary 5.5 and Lemma 5.6, in order to show the convergence of $\Delta _{\lfloor nt\rfloor }/r_n$, it suffices to show the convergence of the scaled sum of independent random variables $\tilde{\chi }_{t,n}/r_n$.

## Excursion times in dense branches

In this section we only consider IVFE. The main tool will be Theorem 6, which is Theorem 10.2 in [4], and is itself a consequence of Theorem IV.6 in [23].

### Theorem 6

Let $n(t):[0,\infty )\rightarrow \mathbb {N}$ and for each *t* let $\{R_k(t)\}_{k=1}^{n(t)}$ be a sequence of i.i.d. random variables. Assume that for every $\epsilon >0$ it is true that

$$\begin{aligned} \lim _{t\rightarrow \infty }\mathbb {P}(R_1(t)>\epsilon )=0. \end{aligned}$$

Now let $\mathcal {L}(x):\mathbb {R}{\setminus }\{0\}\rightarrow \mathbb {R}$ be a real, non-decreasing function satisfying $\lim _{x\rightarrow \infty }\mathcal {L}(x)=0$ and $\int _0^ax^2\mathrm {d}\mathcal {L}(x)<\infty$ for all $a>0$. Suppose $d \in \mathbb {R}$ and $\sigma \ge 0$, then the following statements are equivalent:

1. As $$t\rightarrow \infty $$
  $$\begin{aligned} \sum _{k=1}^{n(t)}R_k(t) \mathop {\rightarrow }\limits ^{{\mathrm{d}}}R_{d,\sigma ,\mathcal {L}} \end{aligned}$$
  where $R_{d,\sigma ,\mathcal {L}}$ has the law $\mathcal {I}(d,\sigma ,\mathcal {L})$, that is,
  6.1 $$\begin{aligned} \mathbb {E}\left[ e^{itR_{d,\sigma ,\mathcal {L}}}\right] :=\exp \left( idt+\int _0^\infty \left( e^{itx}-1-\frac{itx}{1+x^2}\right) \mathrm {d}\mathcal {L}(x)\right) . \end{aligned}$$
2. For $\tau >0$ let $\overline{R}_\tau (t):=R_1(t)\mathbf {1}_{\{|R_1(t)| \le \tau \}}$ then for every continuity point *x* of $\mathcal {L}$
  $$\begin{aligned} d&= \lim _{t\rightarrow \infty }n(t) \mathbb {E}[\overline{R}_\tau (t)]+\int _{|x|>\tau } \frac{x}{1+x^2}\mathrm {d}\mathcal {L}(x)-\int _{0<|x|\le \tau }\frac{x^3}{1+x^2}\mathrm {d}\mathcal {L}(x), \\ \sigma ^2&= \lim _{\tau \rightarrow 0}\limsup _{t\rightarrow \infty }n(t)Var(\overline{R}_\tau (t)), \\ \mathcal {L}(x)&= {\left\{ \begin{array}{ll} \lim _{t\rightarrow \infty }n(t)\mathbb {P}(R_1(t)\le x) &{} x<0 \\ -\lim _{t\rightarrow \infty }n(t)\mathbb {P}(R_1(t)>x) &{} x>0 \end{array}\right. } \end{aligned}$$

In our case, *n*(*t*) will be the number of large branches up to level $\lfloor nt \rfloor$ and $\{R_k\}_{k=1}^{n(t)}$ independent copies of the time spent in a large branch.

**Fig. 3:**
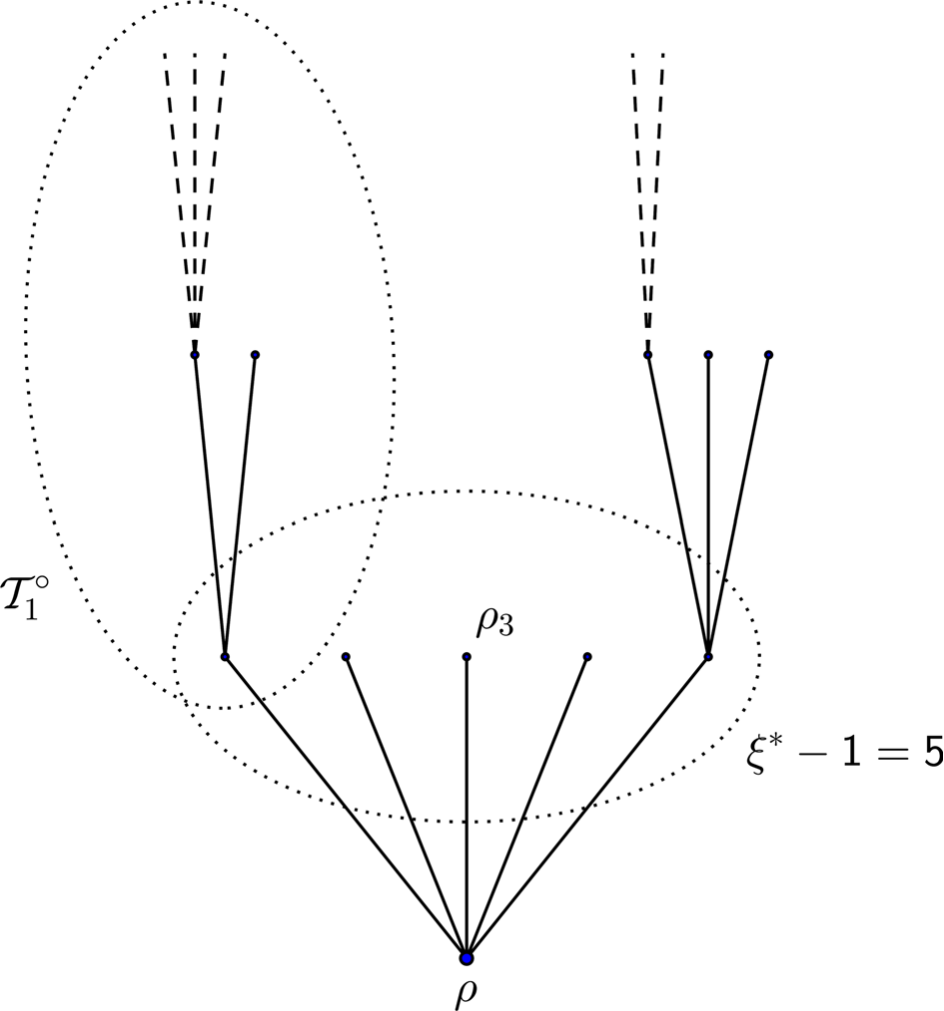
A dummy tree $\mathcal {T}^{*-}$ with five buds, each of which is the root of an independent, unconditioned subcritical GW-tree

Since we are now working with i.i.d. random variables we will simplify notation by considering the dummy branch $\mathcal {T}^{*-}$ defined in Definition 4 which has root $\rho$ and first generation vertices $\rho _1,\ldots ,\rho _{\xi ^*-1}$ which are roots of *f*-GW-trees $(\mathcal {T}_j^\circ )_{j=1}^{\xi ^*-1}$ (Fig. 3). We then let $(W^j)_{j=1}^{\xi ^*-1}$ have the multinomial distribution determined in Lemma 5.1; that is, $W^j$ represents the number of excursions into the $j{\text {th}}$ trap of $\mathcal {T}^{*-}$. For the biased random walk $X_n$ on $\mathcal {T}^{*-}$ started from $\rho$, let $T^{j,k}$ denote the duration of the $k{\text {th}}$ excursion in the $j{\text {th}}$ trap where we recall that in IVFE the excursion starts and ends at the root $\rho$. We then have that

6.2 $$\begin{aligned} \tilde{\chi }_n:= \sum _{j=1}^{\xi ^*-1}\sum _{k=1}^{W^j}T^{j,k}. \end{aligned}$$

is equal in distribution under $\mathbb {P}(\cdot |\xi ^*>l_{n,\varepsilon })$ to $\tilde{\chi }^i_n$ under $\mathbb {P}$ for any *i*.

For $K \ge l_{n,\varepsilon }-l_{n,0}$ write $\overline{L}_{\scriptscriptstyle {K}}:=l_{n,0}+K$ then denote $\mathbb {P}^{\scriptscriptstyle {K}}(\cdot ):=\mathbb {P}\left( \cdot |\xi ^*-1=\overline{L}_{\scriptscriptstyle {K}}\right)$ and $\mathbf {P}^{\scriptscriptstyle {K}}(\cdot ) :=\mathbf {P}\left( \cdot |\xi ^*-1=\overline{L}_{\scriptscriptstyle {K}}\right)$. We now proceed to show that under $\mathbb {P}^{\scriptscriptstyle {K}}$

6.3 $$\begin{aligned} \zeta ^{(n)}:=\frac{1}{\xi ^*-1}\sum _{j=1}^{\xi ^*-1}\sum _{k=1}^{W^j}T^{j,k} \end{aligned}$$

converges in distribution to some random variable $Z_\infty$ whose distribution doesn’t depend on *K*.

We start by showing that the excursion times $T^{j,k}$ don’t differ greatly from $E ^{\mathcal {T}^{*-}}[T^{j,k}]$. In order to do this we require moment bounds on $T^{j,k}$ however since $\mathbf {E}[\xi ^2]=\infty$ we don’t have finite variance of the excursion times and thus we require a more subtle treatment. Recall that for a tree *T* we denote $Z_n^T$ to be the size of the $n{\text {th}}$ generation. Excursion times are first return times $\tau _\rho ^+$ conditioned on the first step therefore pruning buds and using (5.1) we have that the expected excursion time in a trap $\mathcal {T}_j^\circ$ is

6.4 $$\begin{aligned} E ^{\mathcal {T}^{*-}}_\rho \left[ T^{j,k}\right] = E ^{\mathcal {T}^{*-}}_{\rho }\left[ \tau ^+_{\rho }\big |X_1=\rho _j\right] =\sum _{n=0}^\infty Z_n^{\mathcal {T}_j^\circ }\beta ^n \le \mathcal {H}\left( \mathcal {T}_j^\circ \right) \sup _nZ_n^{\mathcal {T}_j^\circ }\beta ^n. \end{aligned}$$

Using that $\mathbf {P}(Z_n^{\mathcal {T}^\circ }>0)\sim c_\mu \mu ^n$ (from (3.1)) we see that for *n* large there are no traps of height greater than $C\log (n)$ for some constant *C* thus for our purposes it will suffice to study $\sup _nZ_n^{\mathcal {T}^\circ }\beta ^n$.

### Lemma 6.1

Let $Z_n$ be a subcritical Galton–Watson process with mean $\mu$ and offspring $\xi$ satisfying $\mathbf {E}[\xi ^{1+\tilde{\varepsilon }}]<\infty$ for some $\tilde{\varepsilon }>0$. Suppose $1<\beta <\mu ^{-1}$, then there exists $\kappa >0$ such that for all $\epsilon \in (0,\kappa )$ we have that $(Z_n\beta ^n)^{1+\epsilon }$ is a supermartingale.

### Proof

Let $\mathcal {F}_n:=\sigma (Z_k; \; k \le n)$ denote the natural filtration of $Z_n$ and $(\xi _k)_{k\ge 1}$ be independent copies of $\xi$.

$$\begin{aligned} \mathbf {E}\left[ \left( Z_n\beta ^n\right) ^{1+\epsilon }\big |\mathcal {F}_{n-1}\right]&= \left( Z_{n-1}\beta ^{n-1}\right) ^{1+\epsilon }\beta ^{1+\epsilon }\mathbf {E}\left[ \left( \sum _{k=1}^{Z_{n-1}}\frac{\xi _k}{Z_{n-1}}\right) ^{1+\epsilon }\Big |Z_{n-1}\right] \\&\le \left( Z_{n-1}\beta ^{n-1}\right) ^{1+\epsilon }\beta ^{1+\epsilon }\mathbf {E}\left[ \sum _{k=1}^{Z_{n-1}}\frac{\xi _k^{1+\epsilon }}{Z_{n-1}}\Big |Z_{n-1}\right] \\&= \left( Z_{n-1}\beta ^{n-1}\right) ^{1+\epsilon }\beta ^{1+\epsilon }\mathbf {E}\left[ \xi ^{1+\epsilon }\right] \end{aligned}$$

where the inequality follows by convexity of $f(x)=x^{1+\epsilon }$. From this it follows that for $\epsilon \in (0,\alpha -1)$

$$\begin{aligned} \mathbf {E}\left[ (Z_n\beta )^{1+\epsilon }\right] \; \le \; \mathbf {E}\left[ (Z_{n-1}\beta )^{1+\epsilon }\right] \mathbf {E}\left[ (\xi \beta )^{1+\epsilon }\right] \; \le \; \mathbf {E}\left[ (\xi \beta )^{1+\epsilon }\right] ^n \; < \; \infty . \end{aligned}$$

Fix $\lambda =(\mu /\beta )^{1/2}$ then $\mu <\lambda$ and for $\epsilon >0$ sufficiently small $\lambda \beta ^{1+\epsilon }<1$. By dominated convergence $\mathbf {E}[\xi ^{1+\epsilon }]<\lambda$ for all $\epsilon$ small. In particular, $\beta ^{1+\epsilon }\mathbf {E}[\xi ^{1+\epsilon }]<1$ for $\epsilon$ suitably small and therefore $(Z_n\beta ^n)^{1+\epsilon }$ is a supermartingale. $\square$

### Lemma 6.2

In IVFE, we can choose $\varepsilon >0$ such that for any $t>0$ there exists a constant $C_t$ such that

$$\begin{aligned} \sup _{K\ge -(a_n-l_{n,\varepsilon })}\mathbb {P}^{\scriptscriptstyle {K}}\left( \left| \frac{1}{\overline{L}_{\scriptscriptstyle {K}}}\sum _{j=1}^{\overline{L}_{\scriptscriptstyle {K}}}\sum _{k=1}^{W^j}(T^{j,k}- E ^{\mathcal {T}^{*-}}\left[ T^{j,1}\right] )\right| >t\right) \le C_tn^{-2\varepsilon }. \end{aligned}$$

### Proof

Write $E_m:=\bigcap _{j=1}^m \left\{ \mathcal {H}(\mathcal {T}_j^\circ ) \le C\log (m)\right\}$ to be the event that none of the first *m* trees have height greater than $C\log (m)$. From (3.1) $\mathbf {P}(\mathcal {H}(\mathcal {T}_j^\circ )\ge m)\sim c_\mu \mu ^m$ hence we can choose $\tilde{C}>c_\mu$ such that

$$\begin{aligned} \mathbf {P}(E_m^c) \; \le \; m\mathbf {P}\left( \mathcal {H}(\mathcal {T}_j^\circ )> C\log (m)\right) \; \le \; \tilde{C}m\mu ^{C\log (m)}. \end{aligned}$$

Thus choosing $C>1/\log (\mu ^{-1})$ and $c=C\log (\mu ^{-1})-1>0$ we have that $\mathbf {P}(E^c_m)\le \tilde{C}m^{-c}$ for *m* sufficiently large. By Lemma 6.1 we have that $(Z_k\beta ^k)^{1+\epsilon }$ is a supermartingale for $\epsilon >0$ sufficiently small where $Z_n$ is the process associated to $\mathcal {T}^\circ$ therefore by Doob’s supermartingale inequality

$$\begin{aligned} \mathbf {P}\left( \sup _{k \le m}Z_k\beta ^k \ge x\right) = \mathbf {P}\left( \sup _{k \le m}(Z_k\beta ^k)^{1+\epsilon } \ge x^{1+\epsilon }\right) \le \mathbf {E}\left[ Z_0^{1+\epsilon }\right] x^{-(1+\epsilon )}. \end{aligned}$$

Using the expression (6.4) for the expected excursion time it follows that

6.5 $$\begin{aligned} \mathbf {P}\left( E ^{\mathcal {T}_j^\circ }\left[ T^{j,1}\right] > x\big |\mathcal {H}\left( \mathcal {T}_j^\circ \right) \le C\log (m)\right) \le C\log (m)^{1+\epsilon }x^{-(1+\epsilon )}. \end{aligned}$$

In particular, for some slowly varying function $\overline{L}$

6.6 $$\begin{aligned} \mathbf {E}\left[ E ^{\mathcal {T}_j^\circ }\left[ T^{j,1}\right] ^2\mathbf {1}_{\left\{ E ^{\mathcal {T}_j^\circ }\left[ T^{j,1}\right] \le m\right\} }\big |\mathcal {H}\left( \mathcal {T}_j^\circ \right) \le C\log (m)\right] \le C\overline{L}(m)m^{1-\epsilon }. \end{aligned}$$

Let $\kappa =\epsilon /(2(1+\epsilon ))$ then write $\overline{E}_m:=E_m \cap \bigcap _{j=1}^m\{ E ^{\mathcal {T}_j^\circ }[T^{j,1}]\le m^{1-\kappa }\}$ to be the event that no trap is of height greater than $C\log (m)$ and the expected excursion time in any trap is at most $m^{1-\kappa }$. For *m* sufficiently large by (6.5) we have that

$$\begin{aligned} \mathbf {P}\left( \overline{E}_m^c\right)&\le \mathbf {P}\left( \bigcup _{j=1}^m\left\{ E ^{\mathcal {T}_j^\circ }\left[ T^{j,1}\right] > m^{1-\kappa }\right\} \Big |\mathcal {H}(\mathcal {T}_j^\circ )\le C\log (m) \; \forall j\le m\right) +\mathbf {P}\left( E_m^c\right) \\&\le mC\log (m)^{1+\epsilon }m^{-(1-\kappa )(1+\epsilon )}+\tilde{C}m^{-c}. \end{aligned}$$

Write $\overline{\overline{E}}_m:=\overline{E}_m\cap \bigcap _{j=1}^m\{W^j\le C'\log (m)\}$ for $C'>(2\beta -1)/(\beta -1)$ to be the event that no trap is of height greater than $C\log (m)$, entered more than $C'\log (m)$ times or has expected excursion time greater than $m^{1-\kappa }$. Then, by a union bound and the geometric distribution of $W^j$ from Lemma 5.1

6.7 $$\begin{aligned} \mathbb {P}\left( \overline{\overline{E}}_m^c\right)&\le \mathbf {P}(\overline{E}_m^c)+m\mathbb {P}(W^1> C'\log (m)) \nonumber \\&\le \tilde{C}\left( \log (m)^{1+\epsilon }m^{1-(1-\kappa )(1+\epsilon )}+m^{-c}+ m^{1-C'\frac{\beta -1}{2\beta -1}}\right) \end{aligned}$$

for *m* large. Since $(1\,-\,\kappa )(1\,+\,\epsilon )>1$ we can choose $\varepsilon <\frac{1}{2}\min \left\{ (1-\kappa )(1+\epsilon )-1, \; c, C'\frac{\beta -1}{2\beta -1}-1 \right\}$ then we have that $\mathbb {P}\left( \overline{\overline{E}}_m^c\right) \le \tilde{C}m^{-2\varepsilon }$ and

$$\begin{aligned}&\mathbb {P}\left( \left| \frac{1}{m}\sum _{j=1}^m\sum _{k=1}^{W^j}(T^{j,k}- E ^{\mathcal {T}_j^\circ }[T_{j,k}])\right| >t\right) \\&\quad \le \mathbb {E}\left[ \frac{\sum _{j=1}^mC\log (m)Var_{ P ^{\mathcal {T}_j^\circ }}\left( \left( T^{j,1}- E ^{\mathcal {T}_j^\circ }\left[ T^{j,1}\right] \right) \mathbf {1}_{\overline{\overline{E}}_m}\right) }{(mt)^2}\right] +\mathbb {P}\left( \overline{\overline{E}}_m^c\right) \\&\quad \le \frac{C\log (m)}{mt^2}m^{(1-\epsilon )}\overline{L}(m)+\tilde{C}m^{-2\varepsilon } \end{aligned}$$

for some slowly varying function $\overline{L}$. Here the first inequality comes from Chebyshev and the second holds due to (6.6). Since $\epsilon >0$ we can choose $\varepsilon \in (0,\epsilon /2)$ then

$$\begin{aligned} \mathbb {P}\left( \left| \frac{1}{m}\sum _{j=1}^m\sum _{k=1}^{W^j}\left( T_{j,k}- E ^{\mathcal {T}_j^\circ }\left[ T_{j,k}\right] \right) \right| >t\right) \le C_tm^{-2\varepsilon }. \end{aligned}$$

In particular, this holds for $m=\overline{L}_{\scriptscriptstyle {K}}\ge a_{n^{1-\varepsilon }}$ thus since $\alpha <2$

$$\begin{aligned} \sup _{K\ge -(a_n-l_{n,\varepsilon })}\mathbb {P}^{\scriptscriptstyle {K}}\left( \left| \frac{1}{\overline{L}_{\scriptscriptstyle {K}}}\sum _{j=1}^{\overline{L}_{\scriptscriptstyle {K}}}\sum _{k=1}^{W^j}(T^{j,k}- E ^{\mathcal {T}_j^\circ }[T^{j,1}])\right| >t\right)&\le C_t \sup _{K\ge -(a_n-l_{n,\varepsilon })}\overline{L}_{\scriptscriptstyle {K}}^{-2\varepsilon } \\&\le C_ta_{n^{1-\varepsilon }}^{-2\varepsilon } \\&= C_tn^{-2\varepsilon }\left( n^{\frac{2-\alpha }{\alpha -1}-\varepsilon }\tilde{L}(n^{1-\varepsilon })\right) ^{-2\varepsilon } \end{aligned}$$

which is bounded above by $C_tn^{-2\varepsilon }$ for *n* large whenever $\varepsilon <(2-\alpha )/(\alpha -1)$. $\square$

Using this we can now show that the average time spent in a trap indeed converges to its expectation.

### Lemma 6.3

In IVFE, we can find $\varepsilon >0$ such that for sufficiently large *n* we have that

$$\begin{aligned} \sup _{K\ge -\left( a_n-l_{n,\varepsilon }\right) } \mathbb {P}^{\scriptscriptstyle {K}}\left( \left| \frac{1}{\overline{L}_{\scriptscriptstyle {K}}} \sum _{j=1}^{\overline{L}_{\scriptscriptstyle {K}}}W^j\left( E ^{\mathcal {T}_j^\circ }\left[ T^{j,1}\right] -\mathbb {E}\left[ T^{1,1}\right] \right) \!\right| >t\right) \le r(n)\left( n^{-\varepsilon }+\frac{C}{t}\right) \end{aligned}$$

uniformly over $t\ge 0$ where $r(n)=o(1)$.

### Proof

We continue using the notation defined in Lemma 6.2 and also define the event

$$\begin{aligned} E_m^j:=\left\{ \mathcal {H}\left( \mathcal {T}_j^\circ \right) \le \tilde{C}\log (m)\right\} \cap \left\{ W^j\le C\log (m)\right\} \cap \left\{ E ^{\mathcal {T}_j^\circ }\left[ T^{j,1}\right] \le m^{1-\kappa }\right\} \end{aligned}$$

that the $j{\text {th}}$ trap isn’t tall, entered many times and that the expected excursion time in it isn’t large.

$$\begin{aligned}&\mathbb {P}\left( \left| \frac{1}{m}\sum _{j=1}^mW^j\left( E ^{\mathcal {T}_j^\circ }\left[ T^{j,1}\right] -\mathbb {E}\left[ T^{1,1}\right] \right) \right|>t\right) \\&\quad \le \mathbb {E}\left[ \mathbb {P}\left( \left| \frac{1}{m}\sum _{j=1}^mW^j\left( E ^{\mathcal {T}_j^\circ }\left[ T^{j,1}\right] \mathbf {1}_{E_m^j}-\mathbb {E}\left[ T^{1,1}\right] \mathbf {1}_{E_m^j}\right) \right| >t\Big | (W^j)_{j=1}^m\right) \right] +o(m^{-\varepsilon }). \end{aligned}$$

Since $\mathbb {E}[ E ^{\mathcal {T}_j^\circ }[T^{j,1}\mathbf {1}_{E_m^j}]]=\mathbb {E}[T^{j,1}\mathbf {1}_{E_m^j}] \ne \mathbb {E}[\mathbb {E}[T^{1,1}]\mathbf {1}_{E_m^j}]$ we have that the summand in the right hand side doesn’t have zero mean thus we perform the splitting:

$$\begin{aligned}&\mathbb {E}\left[ \mathbb {P}\left( \left| \frac{1}{m}\sum _{j=1}^mW^j( E ^{\mathcal {T}_j^\circ }[T^{j,1}]\mathbf {1}_{E_m^j}-\mathbb {E}[T^{j,1}]\mathbf {1}_{E_m^j})\right|>t\Big | (W^j)_{j=1}^m\right) \right] \\&\quad \le \mathbb {E}\left[ \mathbb {P}\left( \left| \frac{1}{m}\sum _{j=1}^mW^j( E ^{\mathcal {T}_j^\circ }[T^{j,1}]\mathbf {1}_{E_m^j}-\mathbb {E}[T^{j,1}\mathbf {1}_{E_m^j}])\right|>t/3\Big | (W^j)_{j=1}^m\right) \right] \\&\qquad + \mathbb {E}\left[ \mathbb {P}\left( \left| \frac{1}{m}\sum _{j=1}^mW^j(\mathbb {E}[T^{j,1}\mathbf {1}_{E_m^j}]-\mathbb {E}[T^{j,1}\mathbf {1}_{E_m^j}]\mathbf {1}_{E_m^j})\right|>t/3\Big | (W^j)_{j=1}^m\right) \right] \\&\qquad + \mathbb {E}\left[ \mathbb {P}\left( \left| \frac{1}{m}\sum _{j=1}^mW^j(\mathbb {E}[T^{j,1}]\mathbf {1}_{E_m^j}-\mathbb {E}[T^{j,1}\mathbf {1}_{E_m^j}]\mathbf {1}_{E_m^j})\right| >t/3\Big | (W^j)_{j=1}^m\right) \right] . \end{aligned}$$

By Chebyshev’s inequality and the tail bound $\mathbb {E}[ E ^{\mathcal {T}_j^\circ }[T^{j,1}]^2\mathbf {1}_{\{E_m^j\}}]\le Cm^{1-\epsilon }L(m)$ from (6.6) we have that the first term is bounded above by

$$\begin{aligned} \mathbb {E}\left[ \frac{C\log (m)^2}{(mt/3)^2}\sum _{j=1}^mVar\left( E ^{\mathcal {T}_j^\circ }\left[ T^{j,1}\right] \mathbf {1}_{E_m^j}\right) \right] \le C_tm^{-\epsilon }\overline{L}(m) \end{aligned}$$

for some slowly varying function $\overline{L}$. The second term is equal to

$$\begin{aligned}&\mathbb {E}\left[ \mathbb {P}\left( \left| \frac{1}{m}\sum _{j=1}^mW^j\mathbb {E}[T^{1,1}\mathbf {1}_{E_m^j}]\mathbf {1}_{(E_m^j)^c}\right| >t/3\Big | (W^j)_{j=1}^m\right) \right] \\&\quad \le \mathbf {P}\left( \bigcup _{j=1}^m(E_m^j)^c\right) = o(m^{-\varepsilon }) \end{aligned}$$

by (6.7). The final term can be written as

$$\begin{aligned} \mathbb {P}\left( \frac{1}{m}\sum _{j=1}^mW^j\mathbb {E}[T^{j,1}\mathbf {1}_{(E_m^j)^c}]\mathbf {1}_{E_m^j}>t/3\right)&\le \frac{3}{mt}\sum _{j=1}^m\mathbb {E}[W^j]\mathbb {E}[T^{j,1}\mathbf {1}_{(E_m^j)^c}] \\&=\frac{C}{t}\mathbb {E}[T^{1,1}\mathbf {1}_{(E_m^1)^c}] \end{aligned}$$

which converges to 0 as $m \rightarrow \infty$ by dominated convergence since, by (5.2), $\mathbb {E}[T^{1,1}]<\infty$. We therefore have that the statement holds by setting $m=\overline{L}_{\scriptscriptstyle {K}}$. $\square$

Recall from (6.3) that, under $\mathbb {P}^{\scriptscriptstyle {K}}$, $\zeta ^{(n)}$ is the average time spent in a trap of a branch with $\xi ^*-1=\overline{L}_{\scriptscriptstyle {K}}$ buds. From Lemmas 6.2 and 6.3 we have that as $n\rightarrow \infty$

$$\begin{aligned} \sup _{K\ge -(a_n-l_{n,\varepsilon })}\mathbb {P}^{\scriptscriptstyle {K}}\left( \left| \zeta ^{(n)}-\mathbb {E}[T^{1,1}]\sum _{j=1}^{\overline{L}_{\scriptscriptstyle {K}}}\frac{W^j}{\overline{L}_{\scriptscriptstyle {K}}}\right| >t\right) \rightarrow 0. \end{aligned}$$

Using (5.1) we have that $\mathbb {E}[T^{1,1}]=2/(1-\beta \mu )$. Write $\theta =(\beta -1)(1-\beta \mu )/(2\beta )$ and let $Z^\infty \sim \exp (\theta )$.

### Corollary 6.4

In IVFE, we can find $\varepsilon >0$ such that for sufficiently large *n* we have that

$$\begin{aligned} \sup _{K\ge -(a_n-l_{n,\varepsilon })}\left| \mathbb {P}^{\scriptscriptstyle {K}}\left( \zeta ^{(n)}>t\right) -\mathbb {P}\left( Z^\infty >t\right) \right| \le \tilde{r}(n)\left( n^{-\varepsilon }+\frac{C}{t} \right) \end{aligned}$$

uniformly over $t\ge 0$ where $\tilde{r}(n)=o(1)$.

### Proof

By Lemma 5.1 the sum of $W^j$ have a geometric law. In particular,

$$\begin{aligned}&\left| \mathbb {P}(Z^\infty>t)-\mathbb {P}^{\scriptscriptstyle {K}}\left( \mathbb {E}[T^{1,1}]\sum _{j=1}^{\overline{L}_{\scriptscriptstyle {K}}}\frac{W^j}{\overline{L}_{\scriptscriptstyle {K}}}>t\right) \right| \\&\quad =\left| e^{-\theta t}-\mathbb {P}^{\scriptscriptstyle {K}}\left( Geo\left( \frac{\beta -1}{(\overline{L}_{\scriptscriptstyle {K}}+1)\beta -1}\right) >\frac{\overline{L}_{\scriptscriptstyle {K}}t}{\mathbb {E}[T^{1,1}]}\right) \right| \\&\quad = \left| e^{-\theta t}-\left( 1-\frac{\beta -1}{(\overline{L}_{\scriptscriptstyle {K}}+1)\beta -1}\right) ^{\left\lceil \frac{\overline{L}_{\scriptscriptstyle {K}}t}{\mathbb {E}[T^{1,1}]}\right\rceil } \right| \\&\quad = \left| e^{-\theta t}-e^{-\theta t \frac{\overline{L}_{\scriptscriptstyle {K}} \beta }{\overline{L}_{\scriptscriptstyle {K}}\beta +\beta -1}} \right| +o\left( \overline{L}_{\scriptscriptstyle {K}}^{-1}\right) \\&\quad \le Ce^{-\theta t}\overline{L}_{\scriptscriptstyle {K}}^{-1} +o\left( \overline{L}_{\scriptscriptstyle {K}}^{-1}\right) \end{aligned}$$

for some constant *C* independent of *K*. It therefore follows that the laws of $\zeta ^{(n)}$ converge under $\mathbb {P}^{\scriptscriptstyle {K}}$ to an exponential law. In particular, using Lemmas 6.2 and 6.3 with the bound

$$\begin{aligned}&\left| \mathbb {P}^{\scriptscriptstyle {K}}\left( \zeta ^{(n)}>t\right) -\mathbb {P}\left( \mathbb {E}[T^{1,1}]\sum _{j=1}^{\overline{L}_{\scriptscriptstyle {K}}}\frac{W^j}{\overline{L}_{\scriptscriptstyle {K}}}>t\right) \right| \\&\quad \le \mathbb {P}^{\scriptscriptstyle {K}}\left( \left| \frac{1}{\overline{L}_{\scriptscriptstyle {K}}}\sum _{j=1}^{\overline{L}_{\scriptscriptstyle {K}}}W^j\left( E ^{\mathcal {T}_j^\circ }\left[ T^{j,1}\right] -\mathbb {E}\left[ T^{1,1}\right] \right) \right| >\epsilon \right) \\&\qquad + \mathbb {P}\left( Z^\infty \in \left[ t-\epsilon ,t+\epsilon \right] \right) +O\left( \overline{L}_{\scriptscriptstyle {K}}^{-1}\right) \end{aligned}$$

with $\epsilon =r(n)^{1/2}t$, we have the result since $\overline{L}_{\scriptscriptstyle {K}}\ge l_{n,\varepsilon } \gg n^\varepsilon$. $\square$

### Corollary 6.5

In IVFE, for any $\tau >0$ fixed

$$\begin{aligned} \lim _{n \rightarrow \infty }\sup _{C\ge 0}\sup _{K\ge -(a_n-l_{n,\varepsilon })}(C\vee 1)\left| \mathbb {E}\left[ Z^\infty \mathbf {1}_{\{CZ^\infty \le \tau \}}\right] -\mathbb {E}^{\scriptscriptstyle {K}}\left[ \zeta ^{(n)}\mathbf {1}_{\{C\zeta ^{(n)}\le \tau \}}\right] \right| =0. \end{aligned}$$

Lemma 6.6 shows that the product of an exponential random variable with a heavy tailed random variable has a similar tail to the heavy tailed variable.

### Lemma 6.6

Let $X \sim exp(\theta )$ and $\xi$ be an independent variable which belongs to the domain of attraction of a stable law of index $\alpha \in (0,2)$. Then $\mathbf {P}(X\xi>x) \sim \theta ^{-\alpha }\Gamma (\alpha +1)\mathbf {P}(\xi >x)$ as $x \rightarrow \infty$.

### Proof

For some slowly varying function *L* we have that $\mathbf {P}(\xi \ge t) \sim x^{-\alpha }L(x)$ as $x \rightarrow \infty$.

Fix $0<u<1<v<\infty$ then $\forall y \le u$ we have that $x/y>x$ thus $\mathbf {P}(\xi \ge x/y)\le \mathbf {P}(\xi \ge x)$ it therefore follows that

$$\begin{aligned} 0 \le \int _0^u \theta e^{-\theta y}\frac{\mathbf {P}(\xi \ge x/y)}{\mathbf {P}(\xi \ge x)}\mathrm {d}y \le \int _0^u \theta e^{-\theta y}\mathrm {d}y = 1-e^{\theta u}. \end{aligned}$$

For $y \in [u,v]$ we have that $\mathbf {P}(\xi \ge x/y)/\mathbf {P}(\xi \ge x)\rightarrow y^\alpha$ uniformly over *y* therefore

$$\begin{aligned} \lim _{x\rightarrow \infty }\int _u^v \theta e^{-\theta y}\frac{\mathbf {P}(\xi \ge x/y)}{\mathbf {P}(\xi \ge x)}\mathrm {d}y = \int _u^v\theta e^{-\theta y}y^\alpha \mathrm {d}y. \end{aligned}$$

Moreover, since this holds for all $u \ge 0$ and $1-e^{\theta u} \rightarrow 0$ as $u \rightarrow 0$ we have that

6.8 $$\begin{aligned} \lim _{x \rightarrow \infty }\int _0^v \theta e^{-\theta y}\frac{\mathbf {P}(\xi \ge x/y)}{\mathbf {P}(\xi \ge x)}\mathrm {d}y = \int _0^v\theta e^{-\theta y}y^\alpha \mathrm {d}y. \end{aligned}$$

Since $0<\mathbf {P}(\xi \ge x)\le 1$ for all $x<\infty$ we have that *L* is bounded away from $0,\infty$ on any compact interval thus satisfies the requirements of Potter’s theorem (see for example [6], 1.5.4) that if *L* is slowly varying and bounded away from $0,\infty$ on any compact subset of $[0,\infty )$ then for any $\epsilon >0$ there exists $A_\epsilon >1$ such that for $x,y>0$

$$\begin{aligned} \frac{L(z)}{L(x)}\le A_\epsilon \max \left\{ \left( \frac{z}{x}\right) ^\epsilon , \left( \frac{x}{z}\right) ^\epsilon \right\} . \end{aligned}$$

Moreover, $\exists c_1,c_2>0$ such that $c_1t^{-\alpha }L(t) \le \mathbf {P}(\xi \ge t) \le c_2t^{-\alpha }L(t)$ hence we have that for all $y >v$ $\mathbf {P}(\xi \ge x/y)/\mathbf {P}(\xi \ge x) \le Cy^{\alpha +\epsilon }$. By dominated convergence we therefore have that

$$\begin{aligned} \lim _{x \rightarrow \infty }\int _v^\infty \theta e^{-\theta y}\frac{\mathbf {P}(\xi \ge x/y)}{\mathbf {P}(\xi \ge x)}\mathrm {d}y =\int _v^\infty \theta e^{-\theta y}y^{\alpha }\mathrm {d}y. \end{aligned}$$

Combining this with (6.8) we have that

$$\begin{aligned} \lim _{x \rightarrow \infty }\frac{\mathbf {P}(X\xi \ge x)}{\mathbf {P}(\xi \ge x)}&=\lim _{x \rightarrow \infty }\int _0^\infty \theta e^{-\theta y}\frac{\mathbf {P}(\xi \ge x/y)}{\mathbf {P}(\xi \ge x)}\mathrm {d}y \\&=\int _0^\infty \theta e^{-\theta y}y^{\alpha }\mathrm {d}y = \theta ^{-\alpha }\Gamma (\alpha +1). \end{aligned}$$

$$\square $$

We write $\mathbb {P}^>(\cdot ):=\mathbb {P}(\cdot | \xi ^*>l_{n,\varepsilon })$ and $\mathbf {P}^>(\cdot ):=\mathbf {P}(\cdot | \xi ^*>l_{n,\varepsilon })$ to be the laws conditioned on the branch $\mathcal {T}^{*-}$ being large. From (6.2) we have that (under $\mathbb {P}$) $\tilde{\chi }^i_n$ are independent copies of the time spent in a branch $\tilde{\chi }_n$ with respect to $\mathbb {P}^>$. Define $\tilde{\chi }^\infty _n:= (\xi ^*-1)Z^\infty$ where $Z^\infty$ is the exponential random variable used in Corollaries 6.4 and 6.5. Recall that $R_{d,\sigma ,\mathcal {L}}$ has the infinitely divisible given by (6.1). Fix the sequence $(\lambda _n)_{n\ge 1}$ converging to some $\lambda >0$ and denote $M_n^\lambda :=\lfloor \lambda _nn^\varepsilon \rfloor$.

### Proposition 6.7

In IVFE, for any $\lambda >0$, as $n \rightarrow \infty$

$$\begin{aligned} \sum _{i=1}^{M_n^\lambda }\frac{\tilde{\chi }^i_n}{a_n} \mathop {\rightarrow }\limits ^{{\mathrm{d}}}R_{d_\lambda ,0,\mathcal {L}_\lambda } \end{aligned}$$

where

$$\begin{aligned} d_\lambda&= \int _0^\infty \frac{x}{1+x^2}\mathrm {d}\mathcal {L}_\lambda (x) \\ \mathcal {L}_\lambda (x)&= {\left\{ \begin{array}{ll}0 &{} x <0 \\ -\lambda x^{-(\alpha -1)}\theta ^{-(\alpha -1)}\Gamma (\alpha ) &{} x>0. \end{array}\right. } \end{aligned}$$

### Proof

Let $\epsilon >0$ then by Markov’s inequality

$$\begin{aligned} \mathbb {P}^>\left( \frac{\tilde{\chi }_n}{a_n}>\epsilon \right)&\le \mathbb {P}^>(\xi ^*-1\ge a_{n^{1-\varepsilon /2}})+\mathbb {P}\left( \sum _{j=1}^{a_{n^{1-\varepsilon /2}}}\sum _{k=1}^{W^j}T^{j,k}\ge \epsilon a_n\right) \\&\le \frac{\mathbb {P}(\xi ^*-1\ge a_{n^{1-\varepsilon /2}})}{\mathbb {P}(\xi ^*-1\ge a_{n^{1-\varepsilon }})} + \frac{a_{n^{1-\varepsilon /2}}}{\epsilon a_n}\mathbb {E}[W^1]\mathbb {E}[T^{1,1}], \end{aligned}$$

which converges to 0 as $n \rightarrow \infty$. Thus, by Theorem 6, it suffices to show that

1. $$\begin{aligned} \lim _{\tau \rightarrow 0^+}\limsup _{n \rightarrow \infty } M_n^\lambda Var_{\mathbb {P}^>}\left( \frac{\tilde{\chi }_n}{a_n} \mathbf {1}_{\{\frac{\tilde{\chi }_n}{a_n} \le \tau \}}\right) =0, \end{aligned}$$
2. $$\begin{aligned} \mathcal {L}_\lambda (x)={\left\{ \begin{array}{ll} \lim _{n \rightarrow \infty } M_n^\lambda \mathbb {P}^>\left( \frac{\tilde{\chi }_n}{a_n} \le x\right) &{} x<0, \\ -\lim _{n \rightarrow \infty } M_n^\lambda \mathbb {P}^>\left( \frac{\tilde{\chi }_n}{a_n}>x\right) &{} x>0, \\ \end{array}\right. } \end{aligned}$$
3. $$\begin{aligned} d_\lambda= & {} \lim _{n \rightarrow \infty } M_n^\lambda \mathbb {E}^>\left[ \frac{\tilde{\chi }_n}{a_n} \mathbf {1}_{\{\frac{\tilde{\chi }_n}{a_n} \le \tau \}}\right] +\int _{|x|>\tau }\frac{x}{1+x^2} d\mathcal {L}_\lambda (x)\\&-\int _{0<|x|\le \tau }\frac{x^3}{1+x^2}d\mathcal {L}_\lambda (x) \end{aligned}$$

where $d_\lambda$ and $\mathcal {L}_\lambda$ are as stated above.

We start with the first condition and since $\lambda _n \rightarrow \lambda$ there exists a constant *C* such that

6.9 $$\begin{aligned} M_n^\lambda Var_{\mathbb {P}^>}\left( \frac{\tilde{\chi }_n}{a_n} \mathbf {1}_{\{\frac{\tilde{\chi }_n}{a_n} \le \tau \}}\right)&\le Cn^\varepsilon \mathbb {E}^>\left[ \left( \frac{\tilde{\chi }_n}{a_n}\right) ^2\mathbf {1}_{\left\{ \frac{\tilde{\chi }_n}{a_n} \le \tau \right\} }\right] \nonumber \\&\le Cn^\varepsilon \left( \tau ^2\mathbf {P}^>(\xi ^*-1\ge a_n) +\tau \mathbb {E}^>\left[ \frac{\tilde{\chi }_n}{a_n} \mathbf {1}_{\left\{ \xi ^*-1<a_n\right\} }\right] \right) . \end{aligned}$$

By the definition of $a_n$ we have that

6.10 $$\begin{aligned} \mathbf {P}^>(\xi ^*-1\ge a_n) =\frac{\mathbf {P}(\xi ^*\ge a_n)}{\mathbf {P}(\xi ^*\ge a_{n^{1-\varepsilon }})} \sim n^{-\varepsilon }. \end{aligned}$$

Conditional on the number of buds $\xi ^*$ we have that the number of excursions $W^j$ into the $j{\text {th}}$ trap are independent from the excursion times $T^{j,k}$ and both the number of excursions and the excursion times have finite mean hence

$$\begin{aligned} \mathbb {E}^>\left[ \frac{\tilde{\chi }_n}{a_n} \mathbf {1}_{\{\xi ^*-1<a_n\}}\right]&=\sum _{r=a_{n^{1-\varepsilon }}}^{a_n-1}\frac{\mathbf {P}(\xi ^*-1=r)}{\mathbf {P}(\xi ^*-1\ge a_{n^{1-\varepsilon }})}\mathbb {E}\left[ \sum _{j=1}^r\sum _{k=1}^{W^j}\frac{T^{j,k}}{a_n}\Big | \xi ^*-1=r\right] \\&\le \frac{\mathbb {E}[W^1]\mathbb {E}[T^{1,1}]}{\mathbf {P}(\xi ^*-1\ge a_{n^{1-\varepsilon }})} \mathbf {E}\left[ \frac{\xi ^*-1}{a_n}\mathbf {1}_{\{\xi ^*-1\le a_n\}}\right] \\&\sim C n^{-\varepsilon } \end{aligned}$$

where the asymptotic holds as $n \rightarrow \infty$ by (5.4). In particular, by combining this with (6.10) in (6.9) we have that $M_n^\lambda Var_{\mathbb {P}^>}(\frac{\tilde{\chi }_n}{a_n} \mathbf {1}_{\{\frac{\tilde{\chi }_n}{a_n} \le \tau \}}) \le C(\tau ^2+\tau )$ for some constant *C* depending on $\lambda$ hence, as $\tau \rightarrow 0^+$, we indeed have convergence to 0 and therefore the first condition holds.

We now move on to the Lévy spectral function $\mathcal {L}_\lambda$. Clearly for $x<0$ we have that $\mathcal {L}_\lambda (x)=0$ since $\tilde{\chi }_n$ is a positive random variable. It therefore suffices to consider $x>0$. By Corollary 6.4 we have that the scaled time spent in a large trap $\zeta ^{(n)}$ [from (6.3)] converges in distribution to an exponential random variable $Z^\infty$ with parameter $\theta$ (which is independent of *K*) therefore, since $M_n^\lambda \sim \lambda n^\varepsilon$ and $\tilde{\chi }^\infty _n=(\xi ^*-1)Z^\infty$ we have that

$$\begin{aligned} M_n^\lambda \mathbb {P}^>\left( \frac{\tilde{\chi }^\infty _n}{a_n}>x\right)&\sim \lambda n^\varepsilon \mathbb {P}^>\left( (\xi ^*-1)Z^\infty>xa_n\right) \nonumber \\&\sim \frac{\lambda }{\mathbf {P}\left( \xi ^*-1\ge a_n\right) } \sum _{K\ge l_{n,\varepsilon }-l_{n,0}}\mathbf {P}\left( \xi ^*-1 =\overline{L}_{\scriptscriptstyle {K}}\right) \mathbb {P}\left( \overline{L}_{\scriptscriptstyle {K}}Z^\infty>xa_n\right) \nonumber \\&= \lambda \frac{\mathbb {P}\left( (\xi ^*-1)Z^\infty \ge xa_n\right) }{\mathbf {P}\left( \xi ^*-1\ge a_n\right) }-\sum _{j=0}^{l_{n,\varepsilon }-1} \frac{\lambda \mathbf {P}\left( \xi ^*-1=j\right) \mathbb {P}\left( jZ^\infty >xa_n\right) }{\mathbf {P}\left( \xi ^*-1\ge a_n\right) } \nonumber \\&\sim \lambda \theta ^{-(\alpha -1)}\Gamma (\alpha ) x^{-(\alpha -1)}. \end{aligned}$$

Where the final asymptotic holds by Lemma 6.6 and because

$$\begin{aligned} \sum _{j=0}^{l_{n,\varepsilon }-1}\frac{\lambda \mathbf {P}\left( \xi ^*-1=j\right) \mathbb {P}\left( jZ^\infty>xa_n\right) }{\mathbf {P}\left( \xi ^*-1\ge a_n\right) } \le \lambda \frac{\mathbb {P}\left( Z^\infty >xa_n/a_{n^{1-\varepsilon }}\right) }{\mathbf {P}\left( \xi ^*-1\ge a_n\right) } = \lambda \frac{e^{-\theta x \frac{a_n}{l_{n,\varepsilon }}}}{\mathbf {P}\left( \xi ^*-1\ge a_n\right) } \end{aligned}$$

which converges to 0 as $n \rightarrow \infty$ since $l_{n,\varepsilon }=a_{\lfloor n^{1-\varepsilon }\rfloor }$ (and therefore $a_n/l_{n,\varepsilon }>>n^\varepsilon$). It now suffices to show that $n^\varepsilon \left( \mathbb {P}^>\left( \frac{\tilde{\chi }^\infty _n}{a_n}>x\right) -\mathbb {P}^>\left( \frac{\tilde{\chi }_n}{a_n}>x\right) \right)$ converges to 0 as $n \rightarrow \infty$. To do this we condition on the number of buds:

$$\begin{aligned}&\mathbb {P}^>\left( \frac{\tilde{\chi }^\infty _n}{a_n}>x\right) -\mathbb {P}^>\left( \frac{\tilde{\chi }_n}{a_n}>x\right) \\&\quad =\sum _{K\ge l_{n,\varepsilon }-l_{n,0}} \mathbf {P}^>\left( \xi ^*-1=\overline{L}_{\scriptscriptstyle {K}}\right) \left( \mathbb {P}\left( \frac{\overline{L}_{\scriptscriptstyle {K}} Z^\infty }{a_n}>x\right) -\mathbb {P}^{\scriptscriptstyle {K}} \left( \frac{\overline{L}_{\scriptscriptstyle {K}} \zeta ^{(n)}}{a_n}>x\right) \right) . \end{aligned}$$

We consider positive and negative *K* separately. For $K \ge 0$ we have that

6.11 $$\begin{aligned}&\sum _{K=0}^\infty n^\varepsilon \mathbf {P}^>\left( \xi ^*-1=\overline{L}_{\scriptscriptstyle {K}}\right) \left| \mathbb {P}\left( \frac{\overline{L}_{\scriptscriptstyle {K}}Z^\infty }{a_n}>x\right) -\mathbb {P}^{\scriptscriptstyle {K}} \left( \frac{\overline{L}_{\scriptscriptstyle {K}}\zeta ^{(n)}}{a_n}>x\right) \right| \nonumber \\&\quad \le n^\varepsilon \mathbf {P}^>\left( \xi ^*-1\ge a_n\right) \sup _{\begin{array}{c} \scriptscriptstyle {c\le 1} \\ \scriptscriptstyle {K\ge 0} \end{array}} \left| \mathbb {P}^{\scriptscriptstyle {K}}(Z^\infty>cx) -\mathbb {P}^{\scriptscriptstyle {K}}(\zeta ^{(n)}>cx)\right| . \end{aligned}$$

By (6.10) $n^\varepsilon \mathbf {P}^>(\xi ^*-1\ge a_n)$ converges as $n \rightarrow \infty$ hence, using Corollary 6.4, (6.11) converges to 0. For $K\le 0$, by Corollary 6.4 we have that

$$\begin{aligned}&\sum _{K=-\infty }^0 \mathbf {1}_{\left\{ K \ge l_{n,\varepsilon }-l_{n,0}\right\} }n^\varepsilon \mathbf {P}^>\left( \xi ^*-1=\overline{L}_{\scriptscriptstyle {K}}\right) \left| \mathbb {P}\left( \frac{\overline{L}_{\scriptscriptstyle {K}} Z^\infty }{a_n}>x\right) -\mathbb {P}^{\scriptscriptstyle {K}}\left( \frac{\overline{L}_{\scriptscriptstyle {K}} \zeta ^{(n)}}{a_n}>x\right) \right| \\&\quad \le n^\varepsilon \sum _{K=-\infty }^0\mathbf {1}_{\left\{ K \ge l_{n,\varepsilon }-l_{n,0}\right\} }\mathbf {P}^>\left( \xi ^*-1= \overline{L}_{\scriptscriptstyle {K}}\right) \tilde{r}(n) \left( n^{-\varepsilon }+\frac{C_x \overline{L}_{\scriptscriptstyle {K}}}{a_n}\right) \\&\quad \le o(1)+\frac{C_x\tilde{r}(n)n^\varepsilon }{a_n}\sum _{K=-\infty }^0\mathbf {1}_{\left\{ K \ge l_{n,\varepsilon }-l_{n,0}\right\} }\frac{\mathbf {P}\left( \xi ^*-1 =\overline{L}_{\scriptscriptstyle {K}}\right) }{\mathbf {P}\left( \xi ^*-1\ge l_{n,\varepsilon }\right) }\overline{L}_{\scriptscriptstyle {K}}. \end{aligned}$$

For some constant *C* we have that $\mathbf {P}(\xi ^*-1\ge l_{n,\varepsilon })\sim Cn^{-(1-\varepsilon )}$ thus by (5.4)

$$\begin{aligned}&\frac{C_x\tilde{r}(n)n^\varepsilon }{a_n}\sum _{K=-\infty }^0\mathbf {1}_{\{K \ge l_{n,\varepsilon }-l_{n,0}\}}\frac{\mathbf {P}\left( \xi ^*-1=\overline{L}_{\scriptscriptstyle {K}}\right) }{\mathbf {P}\left( \xi ^*-1\ge l_{n,\varepsilon }\right) }\overline{L}_{\scriptscriptstyle {K}} \\&\quad \le C_x\tilde{r}(n)n \mathbb {E}\left[ \frac{\xi ^*-1}{a_n}\mathbf {1}_{\{\xi ^*-1\le a_n\}}\right] \sim C_x\tilde{r}(n). \end{aligned}$$

In particular, since $\tilde{r}(n)=o(1)$, we indeed have that this converges to zero and thus we have the required convergence for $\mathcal {L}_\lambda$.

Finally, we consider the drift term $d_\lambda$. Since $\int _{0<x\le \tau }x\mathrm {d}\mathcal {L}_\lambda (x)<\infty$ we have that

$$\begin{aligned} d_\lambda&= \lim _{n \rightarrow \infty } M_n^\lambda \mathbb {E}^>\left[ \frac{\tilde{\chi }_n}{a_n} \mathbf {1}_{\left\{ \frac{\tilde{\chi }_n}{a_n} \le \tau \right\} }\right] +\int _0^\infty \frac{x}{1+x^2} d\mathcal {L}_\lambda (x)-\int _0^\tau x\mathrm {d}\mathcal {L}_\lambda (x). \end{aligned}$$

We want to show that $d_\lambda =\int _0^\infty \frac{x}{1+x^2}\mathrm {d}\mathcal {L}_\lambda (x)$ thus we need to show that the other terms cancel. By definition of $\mathbb {P}^>$ we have that

$$\begin{aligned} \mathbb {E}^>\left[ \frac{\tilde{\chi }^\infty _n}{a_n}\mathbf {1}_{\left\{ \frac{\tilde{\chi }^\infty _n}{a_n}\le \tau \right\} }\right] = \frac{1}{\mathbf {P}\left( \xi ^*-1\ge l_{n,\varepsilon }\right) }\mathbb {E}\left[ \frac{(\xi ^*-1)Z^\infty }{a_n}\mathbf {1}_{\left\{ \frac{(\xi ^*-1)Z^\infty }{a_n}\le \tau \right\} \cap \left\{ \xi ^*>l_{n,\varepsilon }\right\} }\right] . \end{aligned}$$

By Lemma 6.6, $(\xi ^*-1)Z^\infty$ belongs to the domain of attraction of a stable law of index $\alpha -1$ and satisfies the scaling properties of $\xi ^*$ (up to a constant factor). Therefore, using that $a_n \gg l_{n,\varepsilon }$, we have that

$$\begin{aligned} M_n^\lambda \mathbb {E}^>\left[ \frac{\tilde{\chi }^\infty _n}{a_n} \mathbf {1}_{\left\{ \frac{\tilde{\chi }^\infty _n}{a_n}\le \tau \right\} }\right]&\sim \frac{\lambda n^\varepsilon }{\mathbf {P}\left( \xi ^*-1\ge l_{n,\varepsilon }\right) }\mathbb {E}\left[ \frac{(\xi ^*-1)Z^\infty }{a_n}\mathbf {1}_{\left\{ \frac{(\xi ^*-1)Z^\infty }{a_n}\le \tau \right\} }\right] \\&\sim \frac{\alpha -1}{2-\alpha }\tau ^{2-\alpha }\lambda \theta ^{-(\alpha -1)}\Gamma (\alpha ). \end{aligned}$$

Using the form of the Lévy spectral function we have that

$$\begin{aligned} \int _0^\tau x\mathrm {d}\mathcal {L}_\lambda (x) \; = \; \lambda \theta ^{-(\alpha -1)}\Gamma (\alpha ) \int _{\tau ^{-(\alpha -1)}}^\infty x^{-\frac{1}{\alpha -1}}\mathrm {d}x \; = \; \frac{\alpha -1}{2-\alpha }\tau ^{2-\alpha }\lambda \theta ^{-(\alpha -1)}\Gamma (\alpha ) \end{aligned}$$

thus it remains to show that

$$\begin{aligned} n^\varepsilon \left( \mathbb {E}^>\left[ \frac{\tilde{\chi }^\infty _n}{a_n}\mathbf {1}_{\left\{ \frac{\tilde{\chi }^\infty _n}{a_n}\le \tau \right\} }\right] -\mathbb {E}^>\left[ \frac{\tilde{\chi }_n}{a_n}\mathbf {1}_{\left\{ \frac{\tilde{\chi }_n}{a_n}\le \tau \right\} }\right] \right) \rightarrow 0. \end{aligned}$$

Similarly to the previous parts we condition on $\xi ^*-1=\overline{L}_{\scriptscriptstyle {K}}$ and consider the sums over *K* positive and negative separately. For $K \le 0$

$$\begin{aligned}&n^\varepsilon \sum _{K\le 0}\mathbf {P}^>(\xi ^*-1=\overline{L}_{\scriptscriptstyle {K}}) \left| \mathbb {E}\left[ \frac{\overline{L}_{\scriptscriptstyle {K}} Z^\infty }{a_n} \mathbf {1}_{\left\{ \frac{\overline{L}_{\scriptscriptstyle {K}}Z^\infty }{a_n}\le \tau \right\} }\right] -\mathbb {E}^{\scriptscriptstyle {K}} \left[ \frac{\overline{L}_{\scriptscriptstyle {K}} \zeta ^{(n)}}{a_n}\mathbf {1}_{\left\{ \frac{\overline{L}_{\scriptscriptstyle {K}} \zeta ^{(n)}}{a_n}\le \tau \right\} }\right] \right| \\&\quad \le \frac{n^\varepsilon }{\mathbf {P}(\xi ^*\ge l_{n,\varepsilon })}\mathbb {E}\left[ \frac{\xi ^*-1}{a_n}\mathbf {1}_{\left\{ \xi ^*\le a_n\right\} }\right] \; \sup _{K\le 0} \left| \mathbb {E}\left[ Z^\infty \mathbf {1}_{\left\{ Z^\infty \le \tau \frac{a_n}{\overline{L}_{\scriptscriptstyle {K}}} \right\} }\right] -\mathbb {E}^{\scriptscriptstyle {K}} \left[ \zeta ^{(n)}\mathbf {1}_{\left\{ \zeta ^{(n)}\le \tau \frac{a_n}{\overline{L}_{\scriptscriptstyle {K}}} \right\} }\right] \right| . \end{aligned}$$

By definition of $l_{n,\varepsilon }$ and properties of stable laws $n^\varepsilon \mathbb {E}\left[ (\xi ^*-1)/a_n\mathbf {1}_{\{\xi ^*\le a_n\}}\right] /\mathbf {P}(\xi ^*\ge l_{n,\varepsilon })$ converges to some constant as $n \rightarrow \infty$. By Corollary 6.5 we therefore have that this converges to 0. Similarly for $K\ge 0$ we have that

$$\begin{aligned}&n^\varepsilon \sum _{K\ge 0}\mathbf {P}^>(\xi ^*-1=\overline{L}_{\scriptscriptstyle {K}})\left| \mathbb {E}\left[ \frac{\overline{L}_{\scriptscriptstyle {K}}Z^\infty }{a_n}\mathbf {1}_{\left\{ \frac{\overline{L}_{\scriptscriptstyle {K}}Z^\infty }{a_n}\le \tau \right\} }\right] -\mathbb {E}^{\scriptscriptstyle {K}}\left[ \frac{\overline{L}_{\scriptscriptstyle {K}}\zeta ^{(n)}}{a_n}\mathbf {1}_{\left\{ \frac{\overline{L}_{\scriptscriptstyle {K}}\zeta ^{(n)}}{a_n}\le \tau \right\} }\right] \right| \\&\quad \le \frac{n^\varepsilon \mathbf {P}(\xi ^*\ge l_{n,0})}{\mathbf {P}(\xi ^*\ge l_{n,\varepsilon })} \; \sup _{K\ge 0}\frac{\overline{L}_{\scriptscriptstyle {K}}}{a_n}\left| \mathbb {E}\left[ Z^\infty \mathbf {1}_{\left\{ Z^\infty \le \tau \frac{a_n}{\overline{L}_{\scriptscriptstyle {K}}} \right\} }\right] -\mathbb {E}^{\scriptscriptstyle {K}}\left[ \zeta ^{(n)}\mathbf {1}_{\left\{ \zeta ^{(n)}\le \tau \frac{a_n}{\overline{L}_{\scriptscriptstyle {K}}} \right\} }\right] \right| . \end{aligned}$$

We have that $n^\varepsilon \mathbf {P}(\xi ^*\ge l_{n,0})/\mathbf {P}(\xi ^*\ge l_{n,\varepsilon })$ converges to some constant as $n \rightarrow \infty$. The result then follows by Corollary 6.5. $\square$

This shows the convergence result of Theorem 1 in the sense of finite dimensional distributions. In Sect. 9 we prove a tightness result which concludes the proof.

## Excursion times in deep branches

In this section we decompose the time spent in large branches. In FVIE this will be very similar to the decomposition used in [4] and we won’t consider the argument in great detail. However, the decomposition required in IVIE requires greater delicacy.

In Lemmas 7.1, 7.2 and Proposition 7.3 we consider a construction of a GW-tree conditioned on its height from [14] to show that the time spent in deep traps essentially consists of some geometric number of excursions from the deepest point in the trap to itself. That is, as in [4], excursions which don’t reach the deepest point are negligible as is the time taken for the walk to reach the deepest point from the root of the trap and the time taken to return to the root from the deepest point when this happens before returning to the deepest point.

In the remainder of the section we show that, conditional on the exact height of the branch $\overline{H}$, the time spent in the branch scaled by $\beta ^{\overline{H}}$ converges in distribution along the given subsequences. In Lemma 7.5 we determine an important asymptotic relation for the distribution over the number of buds conditional on the height of the branch. In Lemmas 7.6–7.9 we provide various bounds which allow us, in Proposition 7.10, to show that the excursion time in a large branch is close to the random variable $Z_\infty ^n$ (defined in (7.23)) which removes some of the dependency on *n*.

The main result of the section is Proposition 7.14 which shows that the scaled time spent in a large branch converges in distribution along the given subsequences. As a prelude to this we prove Lemmas 7.11–7.13 which show that we can reintroduce small traps into the branch and that the height of a trap is sufficiently close to a geometric random variable. We then conclude the section by showing that the scaled excursion times can be dominated by some random variable with a certain moment property which will be important in Sect. 8.

Recall that $\mathcal {T}^\circ$ is an *f*-GW-tree and $\mathcal {H}(\mathcal {T}^\circ )$ is its height then, following notation of [4], we denote $(\phi _{n+1},\psi _{n+1})_{n \ge 0}$ to be a sequence of i.i.d. pairs with joint law

7.1 $$\begin{aligned}&\mathbf {P}\left( \phi _{n+1}=j,\psi _{n+1}=k\right) \nonumber \\&\quad := \frac{\mathbf {P}(\xi =k)\mathbf {P}\left( \mathcal {H}\left( \mathcal {T}^\circ \right) \le n-1\right) ^{j-1}\mathbf {P}\left( \mathcal {H}(\mathcal {T}^\circ )=n\right) \mathbf {P}\left( \mathcal {H}(\mathcal {T}^\circ )\le n\right) ^{k-j}}{\mathbf {P}\left( \mathcal {H}(\mathcal {T}^\circ )=n+1\right) } \end{aligned}$$

for $k = 1,2,\ldots$ and $j=1,\ldots ,k$. Under this law $\psi _{n+1}$ has the law of the degree of the root of a GW-tree conditioned to be of height $n+1$ and $\phi _{n+1}$ has the law over the first bud to give rise onto a tree of height exactly *n*. We then construct a sequence of trees recursively as follows: Set $\mathcal {T}^\prec _0=\{\delta \}$ then

1. Let the first generation of $\mathcal {T}^\prec _{n+1}$ be of size $\psi _{n+1}$.
2. Attach $\mathcal {T}^\prec _n$ to the $\phi _{n+1}{\text {th}}$ first generation vertex of $\mathcal {T}^\prec _{n+1}$.
3. Attach *f*-GW-trees conditioned to have height at most $n-1$ to the first $\phi _{n+1}-1$ vertices of the first generation of $\mathcal {T}^\prec _{n+1}$.
4. Attach *f*-GW-trees conditioned to have height at most *n* to the remaining $\psi _{n+1}-\phi _{n+1}$ first generation vertices of $\mathcal {T}^\prec _{n+1}$.

**Fig. 4:**
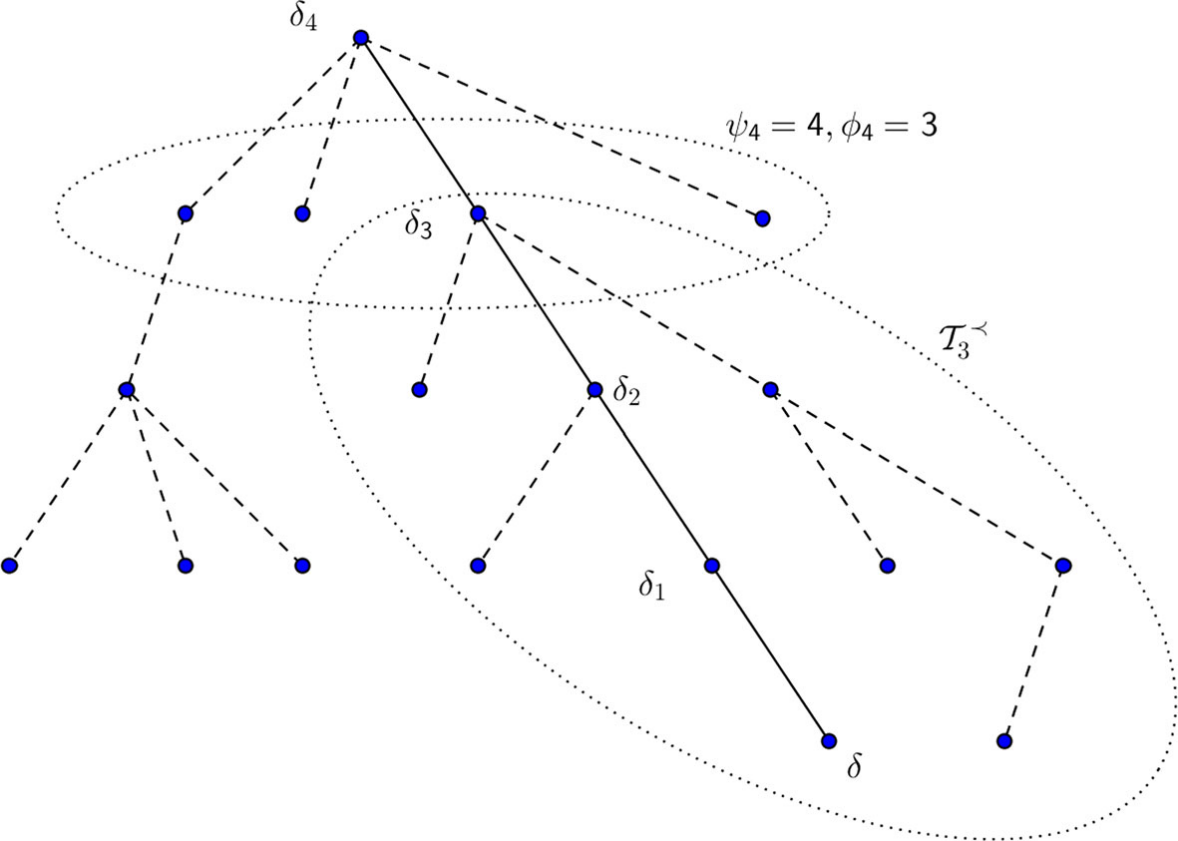
A GW-tree conditioned to be of height 4 with the *solid line* representing the spine and *dashed lines* representing the subtraps which reach at most level 3 to the *left* of the spine and at most level 4 to the *right* of the spine

Under this construction $\mathcal {T}^\prec _{n+1}$ has the distribution of an *f*-GW-tree conditioned to have height exactly $n+1$. Write $\delta _0=\delta$ to be the deepest point of the tree and for $n=1,2,\ldots$ write $\delta _n$ to be the ancestor of $\delta$ of distance *n*. The sequence $\delta _0,\delta _1,\ldots$ form a ‘spine’ from the deepest point to the root of the tree. We denote $\mathcal {T}^\prec$ to be the tree asymptotically attained. By a subtrap of $\mathcal {T}^\prec$ we mean some vertex *x* on the spine together with a descendant *y* off the spine and all of the descendants of *y*. This is itself a tree with root *x* and we write $\mathcal {S}_x$ to be the collection of subtraps rooted at *x*. Figure 4 shows a construction of $\mathcal {T}^\prec _4$ where the solid line represents the spine and the dashed lines represent subtraps.

We denote $\mathcal {S}^{n,j,1}$ to be the $j{\text {th}}$ subtrap conditioned to have height at most $n-1$ attached to $\delta _n$ and $\mathcal {S}^{n,j,2}$ to be the $j{\text {th}}$ subtrap conditioned to have height at most *n* attached to $\delta _n$. Recall that *d*(*x*, *y*) denotes the graph distance between vertices *x*, *y* then for $k=1,2$ let

$$\begin{aligned} \Pi ^{n,j,k}:=2\sum _{x \in \mathcal {S}^{n,j,k}\setminus \left\{ \delta _n\right\} }\beta ^{d(x,\delta _n)} \end{aligned}$$

denote the weight of $\mathcal {S}^{n,j,k}$ under the invariant measure associated to the conductance model with conductances $\beta ^{i+1}$ between levels $i,i+1$ and the roots of $\mathcal {S}^{n,j,k}$ (spinal vertices) denoting level 0. We then write

$$\begin{aligned} \Lambda _n:=\sum _{j=1}^{\phi _n-1}\Pi ^{n,j,1}+\sum _{j=1}^{\psi _n-\phi _n}\Pi ^{n,j,2} \end{aligned}$$

to denote the total weight of the subtraps of $\delta _n$ then,

7.2 $$\begin{aligned} E ^{\mathcal {T}^\prec }[\mathcal {R}_\infty ]=2\sum _{n=0}^\infty \beta ^{-n}(1+\Lambda _n) \end{aligned}$$

is the expected time $\mathcal {R}_\infty$ taken for a walk on $\mathcal {T}^\prec$ started from $\delta$ to return to $\delta$.

### Lemma 7.1

Suppose that $\xi$ belongs to the domain of attraction of a stable law of index $\alpha \in (1,2]$ and $\beta \mu >1$ then

$$\begin{aligned} \mathbb {E}[\mathcal {R}_\infty ]<\infty . \end{aligned}$$

### Proof

Since $\beta >1$ we have that $2\sum _{n=0}^\infty \beta ^{-n}=2/(1-\beta ^{-1})$ thus by (7.2) it suffices to find an appropriate bound on $\mathbf {E}[\Lambda _n]$.

$\mathbf {E}[\Pi ^{n,j,1}]\le \mathbf {E}[\Pi ^{n,j,2}]$ since conditioning the height of the trap to be small only reduces the weight; therefore, by independence of $\psi _n$ and $\Pi ^{n,j,2}$

7.3 $$\begin{aligned} \mathbf {E}\left[ \Lambda _n\right] \; = \; \mathbf {E}\left[ \sum _{j=1}^{\phi _n-1}\Pi ^{n,j,1}+\sum _{j=1} ^{\psi _n-\phi _n}\Pi ^{n,j,2}\right] \; \le \; \mathbf {E}\left[ \Pi ^{n,1,2}\right] \mathbf {E}\left[ \psi _n\right] . \end{aligned}$$

Using that conditioning the height of a GW-tree $\mathcal {T}^\circ$ to be small only decreases the expected generation sizes and that $\mu \beta >1$, by (5.1)

7.4 $$\begin{aligned} \mathbf {E}\left[ \Pi ^{n,1,2}\right] \; = \; 2\sum _{k=1}^n\beta ^k \mathbf {E}\left[ Z_k\big |\mathcal {H}\left( \mathcal {T}^\circ \right) \le n\right] \; \le \; c(\beta \mu )^n \end{aligned}$$

for some constant *c* where $Z_k$ are the generation sizes of $\mathcal {T}^\circ$. Summing over *j* in (7.1) shows that $\mathbf {P}(\psi _{n+1}=k) = \mathbf {P}(Z_1=k|\mathcal {H}(\mathcal {T}^\circ )=n+1)$. Recalling that $s_n=\mathbf {P}(\mathcal {H}(\mathcal {T}^\circ )< n)$,

$$\begin{aligned} \mathbf {E}\left[ \psi _{n+1}\right] \; = \; \mathbf {E}\left[ Z_1\big |\mathcal {H}\left( \mathcal {T}^\circ \right) =n+1\right] \; = \; \sum _{k=1}^\infty kp_k\left( \frac{s_{n+1}^k-s_n^k}{s_{n+2}-s_{n+1}}\right) . \end{aligned}$$

By (3.1) $1-s_{n+1} \sim c\mu ^n$ for some positive constant *c*. Let $\epsilon >0$ be such that $1-\epsilon -\mu (1+\epsilon )>0$, then for *n* large we have that $(1-\epsilon )c\mu ^n\le 1-s_{n+1}\le (1+\epsilon )c\mu ^n$. Therefore,

$$\begin{aligned} s_{n+2}-s_{n+1}=(1-s_{n+1})-(1-s_{n+2}) \ge \left( 1-\epsilon -\mu (1+\epsilon )\right) c\mu ^n \ge C(1-s_n) \end{aligned}$$

for some positive constant *C*. In particular, when $\sigma ^2<\infty$, there exists some constant *c* such that

$$\begin{aligned} \sum _{k=1}^\infty kp_k\left( \frac{s_{n+1}^k-s_n^k}{s_{n+2}-s_{n+1}}\right) \le c\sum _{k=1}^\infty kp_k\left( \frac{1-s_n^k}{1-s_n}\right) \le c\sigma ^2 \end{aligned}$$

where the final inequality comes from that $(1-s^k)(1-s)^{-1}$ is increasing in *s* and converges to *k* for any $k\ge 1$. It therefore follows that $\mathbf {E}[\Lambda _n]\le C(\beta \mu )^n$ so indeed

$$\begin{aligned} \mathbb {E}\left[ \mathcal {R}_\infty \right] \le C\sum _{n=0}^\infty \beta ^{-n}(\beta \mu )^n <\infty . \end{aligned}$$

When $\xi$ has infinite variance but belongs to the domain of attraction of a stable law

$$\begin{aligned} \sum _{k=1}^\infty kp_k\left( s_{n+1}^k-s_n^k\right)&= \mu \left( \left( 1-\frac{s_nf'(s_n)}{\mu }\right) -\left( 1-\frac{s_{n+1}f'(s_{n+1})}{\mu }\right) \right) \end{aligned}$$

hence by (3.9) as $n \rightarrow \infty$ we have that $\mathbf {E}[\psi _{n+1}] \sim c\mu ^{n(\alpha -2)}L_2(\mu ^n)$. Combining this with (7.3) and (7.4) we have

7.5 $$\begin{aligned} \mathbf {E}\left[ \Lambda _n\right] \; \le \; C(\beta \mu )^n\mu ^{n(\alpha -2)}L_2(\mu ^n) \; = \; C(\beta \mu ^{\alpha -1})^nL_2(\mu ^n) \end{aligned}$$

therefore using (7.2) for *C* chosen sufficiently large we have that

$$\begin{aligned} \mathbb {E}\left[ \mathcal {R}_\infty \right]&\le C\left( 1+\sum _{n=1}^\infty \mu ^{n(\alpha -1)}L_2(\mu ^n)\right) <\infty . \end{aligned}$$

$$\square $$

We therefore have that the expected time taken for a walk started from the deepest point in a trap (of height *H*) to return to the deepest point is bounded above by $\mathbb {E}[\mathcal {R}_\infty ]<\infty$ independently of its height. Recall that $\tau ^+_x$ is the first return time to *x*. The following lemma gives the probabilities of reaching the deepest point in a trap, escaping the trap from the deepest point and the transition probabilities for the walk in the trap conditional on reaching the deepest point before escaping. The proof is straightforward by comparison with the biased walk on $\mathbb {Z}$ with nearest neighbour edges so we omit it.

### Lemma 7.2

For any tree *T* of height $H+1$ (with $H \ge 1$), root $\rho$ and deepest vertex $\delta$ we have that

$$\begin{aligned} P ^T_{\delta _H}\left( \tau ^+_\delta <\tau ^+_\rho \right) =\frac{1-\beta ^{-1}}{1-\beta ^{-(H+1)}} \end{aligned}$$

is the probability of reaching the deepest point without escaping and

$$\begin{aligned} P ^{T}_{\delta }\left( \tau ^+_{\rho }<\tau ^+_{\delta }\right) =\frac{1-\beta ^{-1}}{\beta ^H-\beta ^{-1}} \end{aligned}$$

is the probability of escaping from the deepest point before returning. Moreover,

$$\begin{aligned} P ^T_{\delta _k}\left( \tau ^+_{\delta _{k-1}}<\tau ^+_{\delta _{k+1}}\big |\tau ^+_{\delta }<\tau ^+_\rho \right) =\frac{1-\beta ^{-(H+2-k)}}{1-\beta ^{-(H+1-k)}}\cdot \frac{\beta }{\beta +1} \end{aligned}$$

is the probability that the walk restricted to the spine conditioned on reaching $\delta$ before returning to $\rho$ moves towards $\delta$.

Since the first two probabilities are independent of the structure of the tree except for the height we write

7.6 $$\begin{aligned} p_1(H):=\frac{1-\beta ^{-1}}{1-\beta ^{-(H+1)}} \end{aligned}$$

to be the probability that the walk reaches the deepest vertex in the tree before returning to the root starting from the bud and

7.7 $$\begin{aligned} p_2(H):=\frac{1-\beta ^{-1}}{\beta ^{H}-\beta ^{-1}} \end{aligned}$$

to be the probability of escaping from the tree.

For the remainder of the section we will consider only the case that the offspring distribution belongs to the domain of attraction of some stable law of index $\alpha \in (1,2)$. The first aim is to prove Proposition 7.3 which shows that the time on excursions in deep traps essentially consists of some geometric number of excursions from the deepest point to itself. We will then conclude with Corollary 7.4 which is an adaptation for FVIE and of which we omit the proof.

Recall that $\rho _i^+$ is the root of the $i{\text {th}}$ large branch and $\tilde{\chi }_n^i$ is the time spent in this branch by the $i{\text {th}}$ walk $X^{(i)}_n$. This branch has some number $N^i$ buds which are roots of large traps where, by Proposition 3.4, $N^i$ converges to a heavy tailed distribution. Let $\rho ^+_{i,j}$ be the bud of the $j{\text {th}}$ large trap $\mathcal {T}_{i,j}^+$ in this branch then $W^{i,j}:=|\{m\ge 0:X_{m-1}^{(i)}=\rho _i^+, X_m^{(i)}=\rho ^+_{i,j}\}|$ is the number of times that the $j{\text {th}}$ large trap in the $i{\text {th}}$ large branch is visited by the $i{\text {th}}$ copy of the walk. Let $\omega ^{(i,j,0)}:=0$ then for $k\le W^{i,j}$ write $\omega ^{(i,j,k)}:=\min \{m > \omega ^{(i,j,k-1)}:X_{m-1}^{(i)}=\rho _i^+,X_m^{(i)}=\rho ^+_{i,j}\}$ to be the start time of the $k{\text {th}}$ excursion into $\mathcal {T}_{i,j}^+$ and $T^{(i,j,k)}:=|\{m \in [\omega ^{(i,j,k)}, \omega ^{(i,j,k+1)}): X_m^{(i)} \in \mathcal {T}_{i,j}^+\}|$ its duration. We can then write the time spent in large traps of the $i{\text {th}}$ large branch as

$$\begin{aligned} \tilde{\chi }^i_n=\sum _{j=1}^{N^i}\sum _{k=1}^{W^{i,j}}T^{(i,j,k)}. \end{aligned}$$

For $0\le k\le \mathcal {H}(\mathcal {T}_{i,j}^+)$ write $\delta ^{(i,j)}_k$ to be the spinal vertex of distance *k* from the deepest point in $\mathcal {T}_{i,j}^+$. Let $T^{*(i,j,k)}:=0$ if there does not exist $m\in [\omega ^{(i,j,k)},\omega ^{(i,j,k+1)}]$ such that $X_m=\delta _0^{(i,j)}=:\delta ^{(i,j)}$ and

7.8 $$\begin{aligned} T^{*(i,j,k)}&:= \sup \left\{ m \in \left[ \omega ^{(i,j,k)},\omega ^{(i,j,k+1)}\right] : X_m^{(i)}=\delta ^{(i,j)}\right\} \nonumber \\&\qquad -\inf \left\{ m \in \left[ \omega ^{(i,j,k)},\omega ^{(i,j,k+1)}\right] : X_m^{(i)}=\delta ^{(i,j)}\right\} \end{aligned}$$

otherwise to be the duration of the $k{\text {th}}$ excursion into $\mathcal {T}_{i,j}^+$ without the first passage to the deepest point and the final passage from the deepest point to the exit. We can then define

7.9 $$\begin{aligned} \tilde{\chi }^{i*}_n:=\sum _{j=1}^{N^i}\sum _{k=1}^{W^{i,j}}T^{*(i,j,k)} \end{aligned}$$

to be the time spent in the $i{\text {th}}$ large trap without the first passage to and last passage from $\delta ^{(i,j)}$ on each excursion. We want to show that the difference between this and $\tilde{\chi }^i_n$ is negligible. In particular, recalling that $\mathcal {D}_n^{(n)}$ is the collection of large branches by level *n*, we will show that for all $t>0$ as $n \rightarrow \infty$

$$\begin{aligned} \mathbb {P}\left( \left| \sum _{i=1}^{\left| \mathcal {D}_n^{(n)}\right| } \left( \tilde{\chi }^i_n-\tilde{\chi }^{i*}_n\right) \right| \ge ta_n^\frac{1}{\gamma }\right) \rightarrow 0. \end{aligned}$$

For $$\epsilon >0$$ denote

7.10 $$\begin{aligned} A_6(n):= \bigcap _{i=0}^n\left\{ \mathcal {H}\left( \mathcal {T}^{*-}_{\rho _i}\right) \le h_{n,\epsilon }^+\right\} \end{aligned}$$

to be the event that there are no $h_{n,\epsilon }^+$-branches by level *n*. Using a union bound and (3.12) we have that $\mathbf {P}(A_6(n)^c) \le n\mathbf {P}(\mathcal {H}(\mathcal {T}^{*-}_{\rho _0})>h_{n,\epsilon }^+) \rightarrow 0$ as $n \rightarrow \infty$.

Write

7.11 $$\begin{aligned} A_7(n):=\bigcap _{i=0}^{\left| \mathcal {D}_n^{(n)}\right| }\left\{ N^i\le n^{\frac{2\varepsilon }{\alpha -1}}\right\} \end{aligned}$$

to be the event that all large branches up to level *n* of the backbone have fewer than $n^{\frac{2\varepsilon }{\alpha -1}}$ large traps. Conditional on the number of buds, the number of large traps in the branch follows a binomial distribution therefore

$$\begin{aligned} \mathbf {P}\left( N^i\ge n^{\frac{2\varepsilon }{\alpha -1}}\right)\le & {} \frac{\mathbf {P}\left( \xi ^*\ge n^{\frac{1+\varepsilon /2}{\alpha -1}}\right) }{\mathbf {P}\left( \mathcal {H}(\mathcal {T}^{*-})> h_{n,\varepsilon }\right) }\\&+\frac{\mathbf {P}\left( Bin\left( n^{\frac{1+\varepsilon /2}{\alpha -1}}, \mathbf {P}\left( \mathcal {H}\left( \mathcal {T}^\circ \right) \ge h_{n,\varepsilon }\right) \right) \ge n^{\frac{2\varepsilon }{\alpha -1}}\right) }{\mathbf {P}\left( \mathcal {H}\left( \mathcal {T}^{*-}\right) > h_{n,\varepsilon }\right) }. \end{aligned}$$

By (4.1) $\mathbf {P}(\mathcal {H}(\mathcal {T}^{*-})\ge h_{n,\varepsilon })\ge Cn^{-(1-\varepsilon )}$ for *n* large and some constant *C* hence by (3.11) the first term decays faster than $n^{-\varepsilon }$. Using a Chernoff bound the second term has a stretched exponential decay. Therefore, by Lemma 4.1 and a union bound, as $n \rightarrow \infty$

$$\begin{aligned} \mathbf {P}(A_7(n)^c) \le o(1)+Cn^\varepsilon \mathbf {P}\left( N^i\ge n^{\frac{2\varepsilon }{\alpha -1}}\right) \rightarrow 0. \end{aligned}$$

Recall that $d_x:=|c(x)|$ is the number of children of *x* in the tree and define

$$\begin{aligned} A_8(n):=\bigcap _{i=1}^{\left| \mathcal {D}_n^{(n)}\right| } \bigcap _{j=1}^{N^i}\left\{ \sum _{k=0}^{\mathcal {H}(\mathcal {T}_{i,j}^+)}d_{\delta ^{(i,j)}_k} \le n^{3\varepsilon /(\alpha -1)^2}\right\} \end{aligned}$$

to be the event that there are fewer than $n^{3\varepsilon /(\alpha -1)^2}$ subtraps on the spine in any large trap. For $Z_n$ the generation sizes associated to GW-tree $\mathcal {T}^\circ$ we have that $\mathbf {P}(Z_1\ge n|\mathcal {H}(\mathcal {T}^\circ )\ge m)$ is non-decreasing in *m*; therefore, the number of offspring from a vertex on the spine of a trap can be stochastically dominated by the size biased distribution. Using this and Lemma 4.1 with the bounds on $A_6$ and $A_7$ we then have that for some slowly varying function $\overline{L}$

$$\begin{aligned} \mathbf {P}\left( A_8(n)^c\right)&\le o(1)+ Cn^\varepsilon n^{\frac{2\varepsilon }{\alpha -1}} \mathbf {P}\left( \sum _{k=0}^{h_{n,\epsilon }^+}\xi ^*_k\ge n^{3\varepsilon /(\alpha -1)^2}\right) \\&\le o(1)+ Cn^\varepsilon n^{\frac{2\varepsilon }{\alpha -1}}h_{n,\epsilon }^+\mathbf {P}\left( \xi ^* \ge n^{3\varepsilon /(\alpha -1)^2}/h_{n,\epsilon }^+\right) \\&\le o(1)+ n^\varepsilon n^{-\frac{\varepsilon }{\alpha -1}}\overline{L}(n) \end{aligned}$$

where $(\xi ^*_k)_{k\ge 1}$ are independent variables with the size biased law; thus $\mathbf {P}(A_8(n)^c)\rightarrow 0$ as $n \rightarrow \infty$.

### Proposition 7.3

In IVIE, for any $t>0$ as $n \rightarrow \infty$

$$\begin{aligned} \mathbb {P}\left( \left| \sum _{i=1}^{\left| \mathcal {D}_n^{(n)}\right| } \left( \tilde{\chi }^i_n-\tilde{\chi }^{i*}_n\right) \right| \ge ta_n^\frac{1}{\gamma }\right) \rightarrow 0. \end{aligned}$$

### Proof

Let $A'(n):=\bigcap _{i=1}^8A_i(n)$ then using the bounds on $A_i$ for $i=1,\ldots ,8$ it follows that $\mathbb {P}(A'(n)^c)\rightarrow 0$ as $n \rightarrow \infty$. In particular, on $A_1(n)$ (from (4.2)) we have that $|\mathcal {D}_n^{(n)}|\le Cn^\varepsilon$ and on $A_7(n)$ (from (7.11)) we have that $N^i\le n^{\frac{2\varepsilon }{\alpha -1}}$ for all *i* therefore by Markov’s inequality

7.12 $$\begin{aligned}&\mathbb {P}\left( \left| \sum _{i=1}^{|\mathcal {D}_n^{(n)}|} \left( \tilde{\chi }^i_n-\tilde{\chi }^{i*}_n\right) \right| \ge ta_n^\frac{1}{\gamma }\right) \nonumber \\&\quad \le \mathbb {P}(A'(n)^c) +\frac{1}{ta_n^\frac{1}{\gamma }}\mathbb {E}\left[ \mathbf {1}_{A'(n)}\sum _{i=1}^{|\mathcal {D}_n^{(n)}|}\sum _{j=1}^{N^i} \sum _{k=1}^{W^{i,j}}\left( T_n^{(i,j,k)}-T_n^{*(i,j,k)}\right) \right] \nonumber \\&\quad \le o(1) + \frac{Cn^{\varepsilon \left( \frac{\alpha +1}{\alpha -1}\right) }}{ta_n^\frac{1}{\gamma }}\mathbb {E}\left[ \mathbf {1}_{A'(n)} \sum _{k=1}^{W^{(1,1)}}\left( T_n^{(1,1,k)}-T_n^{*(1,1,k)}\right) \right] \end{aligned}$$

where we recall that $T_n^{*(i,j,k)}\le T_n^{(i,j,k)}$ for all *i*, *j*, *k*.

Since the number of excursions $W^{i,j}$ are independent of the excursion times and have marginal distributions of geometric random variables with parameter $(\beta -1)/(2\beta -1)$

$$\begin{aligned} \mathbb {E}\left[ \mathbf {1}_{A'(n)}\sum _{k=1}^{W^{(1,1)}} \left( T_n^{(1,1,k)}-T_n^{*(1,1,k)}\right) \right]&= \mathbb {E}\left[ W^{(1,1)}\right] \mathbb {E}\left[ \mathbf {1}_{A'(n)} \left( T_n^{(1,1,1)}-T_n^{*(1,1,1)}\right) \right] . \end{aligned}$$

For a given excursion either the walk reaches the deepest point $\delta ^{(1,1)}$ before returning to the root $\rho _{1,1}^+$ or it doesn’t. In the first case the difference $T_n^{(1,1,1)}-T_n^{*(1,1,1)}$ is the time taken to reach $\delta ^{(1,1)}$ conditional on the walker reaching $\delta ^{(1,1)}$ before $\rho _{1,1}^+$ added to the time taken to reach $\rho _{1,1}^+$ from $\delta ^{(1,1)}$ conditional on reaching $\rho _{1,1}^+$ before returning to $\delta ^{(1,1)}$. In the second case the difference is the time taken to return to the root given that the walker returns to the root without reaching $\delta ^{(1,1)}$. In particular, recalling that $^{\mathcal {T}_{1,1}^+}$ is the trap rooted at $\rho _{1,1}^+$ we have that

7.13 $$\begin{aligned} \mathbb {E}[\mathbf {1}_{A'(n)}(T_n^{(1,1,1)}-T_n^{*(1,1,1)})] \le&\,\, \mathbf {E}\left[ \mathbf {1}_{A'(n)} E _{\rho _{1,1}^+}^{\mathcal {T}_{1,1}^+} \left[ \mathbf {1}_{A'(n)}\tau ^+_{\delta ^{(1,1)}}\big |\tau ^+_{\delta ^{(1,1)}}<\tau ^+_{\rho _{1,1}^+}\right] \right] \nonumber \\&+ \mathbf {E}\left[ \mathbf {1}_{A'(n)} E _{\delta ^{(1,1)}}^{\mathcal {T}_{1,1}^+} \left[ \mathbf {1}_{A'(n)}\tau ^+_{\rho _{1,1}^+}\big |\tau ^+_{\rho _{1,1}^+}<\tau ^+_{\delta ^{(1,1)}}\right] \right] \nonumber \\&+\mathbf {E}\left[ \mathbf {1}_{A'(n)} E _{\rho _{1,1}^+}^{\mathcal {T}_{1,1}^+} \left[ \mathbf {1}_{A'(n)}\tau ^+_{\rho _{1,1}^+}\big |\tau ^+_{\rho _{1,1}^+}<\tau ^ +_{\delta ^{(1,1)}}\right] \right] .\nonumber \\ \end{aligned}$$

We want to show that each of the terms in (7.13) can be bounded appropriately. This follows similarly to Lemmas 8.2 and 8.3 of [4] so we only sketch the details. Conditional on the event that the walk returns to the root of the trap before reaching the deepest point we have that:

1. the transition probabilities of the walk in subtraps are unchanged,
2. from any vertex on the spine, the walk is more likely to move towards the root than to any vertex in the subtrap,
3. from any vertex on the spine, excluding the root and deepest point, the probability of moving towards the root is at least $\beta$ times that of moving towards the deepest point.

Property 3 above shows that the probability of escaping the trap from any vertex on the spine is at least the probability $p_\infty$ of a regeneration for the $\beta$-biased random walk on $\mathbb {Z}$. From this we have that the number of visits to any spinal vertex can be stochastically dominated by a geometric random variable with parameter $p_\infty$. Similarly, using property 2 above, we see that the number of visits to any subtrap can be stochastically dominated by a geometric random variable with parameter $p_\infty /2$.

Using a union bound with $A_1,A_7,A_8$ and (3.1) we have that with high probability there are no subtraps of height greater than $h_{n,\varepsilon }$. In particular, by (5.2), the expected time in any subtrap can be bounded above by $C(\beta \mu )^{h_{n,\varepsilon }}$ for some constant *C* using property 1. From this it follows that

$$\begin{aligned}&\mathbf {E}\left[ \mathbf {1}_{A'(n)} E _{\rho _{1,1}^+}^{\mathcal {T}_{1,1}^+} \left[ \mathbf {1}_{A'(n)}\tau ^+_{\rho _{1,1}^+}\big |\tau ^+_{\rho _{1,1}^+}<\tau ^+_{\delta ^{(1,1)}}\right] \right] \\&\quad \le \mathbf {E}\left[ \mathbf {1}_{A'(n)} E _{\delta ^{(1,1)}}^{\mathcal {T}_{1,1}^+} \left[ \mathbf {1}_{A'(n)}\tau ^+_{\rho _{1,1}^+}\big |\tau ^+_{\rho _{1,1}^+} <\tau ^+_{\delta ^{(1,1)}}\right] \right] \\&\quad \le o(1) + h_{n,\varepsilon }^+ E \left[ Geo(p_\infty )\right] +Cn^{\frac{3\varepsilon }{(\alpha -1)^2}}(\beta \mu )^{h_{n,\varepsilon }}\\&\quad \le o(1) + C\overline{L}(n)n^{\frac{(1-\varepsilon )}{\alpha -1} \frac{\log (\beta \mu )}{\log (\mu ^{-1})}+\frac{3\varepsilon }{(\alpha -1)^2}} \end{aligned}$$

for some constant *C* and slowly varying function $\overline{L}$.

A symmetric argument shows that the same bound can be achieved for the first term in (7.13). It then follows that the second term in (7.12) can be bounded above by $C_tL_1(n)n^{-\frac{1}{\alpha -1}+\tilde{\varepsilon }}$ where $\tilde{\varepsilon }$ can be made arbitrarily small by choosing $\varepsilon$ sufficiently small. $\square$

A straightforward adaptation of Proposition 8.1 of [4] (similar to the previous calculation) shows Corollary 7.4 which is the corresponding result for FVIE.

### Corollary 7.4

In FVIE, for any $t>0$ as $n \rightarrow \infty$

$$\begin{aligned} \mathbb {P}\left( \left| \sum _{i=1}^{\left| \mathcal {D}_n^{(n)}\right| } \left( \tilde{\chi }^i_n-\tilde{\chi }^{i*}_n\right) \right| \ge tn^\frac{1}{\gamma }\right) \rightarrow 0. \end{aligned}$$

By Proposition 7.3 and Corollary 7.4, in FVIE and IVIE, almost all time up to the walk reaching level *n* is spent on excursions from the deepest point in deep traps. The aim of the remainder of the section is to prove Proposition 7.14 which shows that the time spent on the excursions from the deepest point in a single large branch (suitably scaled) converges in distribution along the given subsequences. To ease notation, for the remainder of the section we work on a dummy branch $\mathcal {T}^*$ so that the time $\tilde{\chi }_n^{i*}$ has the distribution of a sum of excursion times from the deepest points of $\mathcal {T}^*$.

Recall from Definition 4 that $\mathcal {T}^{*-}$ is a dummy branch with root $\rho$, buds $\rho _1,\ldots ,\rho _{\xi ^*-1}$ each of which is the root of an *f*-GW-tree $\mathcal {T}^{\circ }_j$ with height $H_j:=\mathcal {H}(\mathcal {T}^{\circ }_j)$. We now define a pruned version of this branch which only contains traps of height at least $h_{n,\varepsilon }$.

### Definition 8

(*Pruned dummy branch*) Let

7.14 $$\begin{aligned} N:=\sum _{j=1}^{\xi ^*-1}\mathbf {1}_{\left\{ H_j\ge h_{n,\varepsilon }\right\} } \end{aligned}$$

denote the number of traps in $\mathcal {T}^{*-}$ of at least critical height. Denote $(\mathcal {T}^+_j)_{j=1}^{N}$ to be those large traps, $(\rho ^+_j)_{j=1}^N$ their roots and $H_j^+:=\mathcal {H}(\mathcal {T}^+_j)$ the height of the $j{\text {th}}$ large trap in the branch. Similarly, let $(\mathcal {T}^- _j)_{j=1}^{\xi ^*-1-N}$ denote the small traps, $(\rho ^-_j)_{j=1}^{\xi ^*-1-N}$ their roots and $H_j^-:=\mathcal {H}(\mathcal {T}^-_j)$ the height of the $j{\text {th}}$ small trap in the branch.

Let $\mathcal {T}^*$ be $\mathcal {T}^{*-}$ pruned to consist precisely of the root $\rho$, buds $(\rho ^+_j)_{j=1}^N$ and traps $(\mathcal {T}^+_j)_{j=1}^{N}$.

We write $\overline{H}:=\mathcal {H}(\mathcal {T}^*)-1$ to be the height of the largest trap and for $K \in \mathbb {Z}$ let $\overline{H}_n^{\scriptscriptstyle {K}}:= h_{n,0}+K$ then denote

$$\begin{aligned} \mathbb {P}^{\scriptscriptstyle {K}}(\cdot ) :=\mathbb {P}\left( \cdot \big |\overline{H}=\overline{H}_n^{\scriptscriptstyle {K}}\right) \quad \text {and} \quad \mathbf {P}^{\scriptscriptstyle {K}}(\cdot ) :=\mathbf {P}\left( \cdot \big |\overline{H}=\overline{H}_n^{\scriptscriptstyle {K}}\right) . \end{aligned}$$

Write $W^j$ to be the total number of excursions into $\mathcal {T}^+_j$ and $B^j$ the number of excursions which reach the deepest point $\delta ^j$.

For each $k\le B^j$ we define $G^{j,k}$ to be the number of return times to $\delta ^j$ on the $k{\text {th}}$ excursion which reaches $\delta ^j$.

For $l=1,\ldots ,G^{j,k}$ let $\mathcal {R}^{j,k,l}$ denote the duration of the $l{\text {th}}$ excursion from $\delta ^j$ to itself on the $k{\text {th}}$ excursion into $\mathcal {T}^+_j$ which reaches $\delta ^j$.

The height of the branch and the total number of traps in the branch have a strong relationship. Lemma 7.5 shows the exact form of this relationship in the limit as $n \rightarrow \infty$. Recall from (3.1) that $c_\mu$ is the positive constant such that $\mathbf {P}(\mathcal {H}(\mathcal {T})\ge n) \sim c_\mu \mu ^n$ as $n \rightarrow \infty$ then write

7.15 $$\begin{aligned} b_n^{\scriptscriptstyle {K}}:= \frac{\mu ^{-\overline{H}_n^{\scriptscriptstyle {K}}}}{c_\mu }. \end{aligned}$$

### Lemma 7.5

In IVIE, under $\mathbf {P}^{\scriptscriptstyle {K}}$ we have that the sequence of random variables $(\xi ^*-1)/b_n^{\scriptscriptstyle {K}}$ converge in distribution to some random variable $\overline{\xi }$ satisfying

$$\begin{aligned} \mathbf {P}\left( \overline{\xi }\ge t\right) =\frac{\alpha -1}{\Gamma (2-\alpha )\left( 1-\mu ^{\alpha -1}\right) } \int _t^\infty y^{-\alpha }\left( e^{-\mu y}-e^{-y}\right) \mathrm {d}y. \end{aligned}$$

### Proof

We prove this by showing the convergence of

7.16 $$\begin{aligned} \mathbf {P}\left( \xi ^*-1\ge tb_n^{\scriptscriptstyle {K}}\big |\overline{H}= \overline{H}_n^{\scriptscriptstyle {K}}\right) =\mathbf {P}\left( \overline{H}=\overline{H}_n^{\scriptscriptstyle {K}}\big |\xi ^*-1\ge tb_n^{\scriptscriptstyle {K}}\right) \frac{\mathbf {P}\left( \xi ^*-1\ge tb_n^{\scriptscriptstyle {K}}\right) }{\mathbf {P}\left( \overline{H}=\overline{H}_n^{\scriptscriptstyle {K}}\right) } \end{aligned}$$

for all $t>0$. To begin we consider $\mathbf {P}\left( \overline{H}=\overline{H}_n^{\scriptscriptstyle {K}}|\xi ^*-1\ge tb_n^{\scriptscriptstyle {K}}\right)$.

The heights of individual traps are independent under this conditioning hence

$$\begin{aligned} \mathbf {P}\left( \overline{H}\le \overline{H}_n^{\scriptscriptstyle {K}}\big |\xi ^*-1\ge tb_n^{\scriptscriptstyle {K}}\right) = \mathbf {E}\left[ \mathbf {P}\left( \mathcal {H}(\mathcal {T}^\circ )\le \overline{H}_n^{\scriptscriptstyle {K}}\right) ^{\xi ^*-1}\big |\xi ^*-1\ge tb_n^{\scriptscriptstyle {K}}\right] . \end{aligned}$$

We know the asymptotic form of $\mathbf {P}(\mathcal {H}(\mathcal {T}^\circ )\le \overline{H}_n^{\scriptscriptstyle {K}})$ from (3.1) thus we need to consider the distribution of $\xi ^*-1$ conditioned on $\xi ^*-1\ge tb_n^{\scriptscriptstyle {K}}$. By the tail formula for $\xi ^*-1$ following Definition 3 we have that for $r \ge 1$ as $n \rightarrow \infty$

$$\begin{aligned} \mathbf {P}\left( \frac{\xi ^*-1}{tb_n^{\scriptscriptstyle {K}}} \ge r\Big | \xi ^*-1 \ge tb_n^{\scriptscriptstyle {K}}\right) \; = \; \frac{\mathbf {P}\left( \xi ^*-1 \ge rtb_n^{\scriptscriptstyle {K}}\right) }{\mathbf {P}\left( \xi ^*-1 \ge tb_n^{\scriptscriptstyle {K}}\right) } \; \sim \; r^{-(\alpha -1)}. \end{aligned}$$

We therefore have that, conditional on $\xi ^*-1\ge tb_n^{\scriptscriptstyle {K}}$, the sequence $(\xi ^*-1)/tb_n^{\scriptscriptstyle {K}}$ converges in distribution to a variable *Y* with tail $\mathbf {P}(Y\ge r)=r^{-(\alpha -1)} \wedge 1$. Using the form of $b_n^{\scriptscriptstyle {K}}$ we then have that

$$\begin{aligned} \mathbf {P}\left( \mathcal {H}(\mathcal {T}^\circ )\le \overline{H}_n^{\scriptscriptstyle {K}}\right) ^{tb_n^{\scriptscriptstyle {K}}} =e^{-t\mu (1+o(1))}. \end{aligned}$$

It therefore follows that

$$\begin{aligned} \lim _{n \rightarrow \infty }\mathbf {P}\left( \overline{H}\le \overline{H}_n^{\scriptscriptstyle {K}}|\xi ^*-1\ge tb_n^{\scriptscriptstyle {K}}\right) = \mathbf {E}\left[ e^{-t\mu Y}\right] . \end{aligned}$$

Repeating with $\overline{H}_n^{\scriptscriptstyle {K}}$ replaced by $\overline{H}_n^{\scriptscriptstyle {K}}-1$ we have that $\mathbf {P}(\overline{H}=\overline{H}_n^{\scriptscriptstyle {K}}|\xi ^*-1\ge tb_n^{\scriptscriptstyle {K}}) \rightarrow \mathbf {E}[e^{-t\mu Y}] - \mathbf {E}[e^{-tY}]$ as $n \rightarrow \infty$. For $\theta >0$

$$\begin{aligned} \mathbf {E}\left[ e^{-\theta tY}\right] = (\alpha -1)t^{\alpha -1}\int _t^\infty e^{-\theta y}y^{-\alpha }\mathrm {d}y \end{aligned}$$

therefore

7.17 $$\begin{aligned} \lim _{n \rightarrow \infty }\mathbf {P}\left( \overline{H}=\overline{H}_n^{\scriptscriptstyle {K}}\big |\xi ^*-1\ge tb_n^{\scriptscriptstyle {K}}\right) = (\alpha -1)t^{\alpha -1}\int _t^\infty y^{-\alpha }(e^{-\mu y}-e^{-y})\mathrm {d}y.\quad \end{aligned}$$

By (3.12) we have that as $n \rightarrow \infty$

$$\begin{aligned} \mathbf {P}\left( \overline{H}=\overline{H}_n^{\scriptscriptstyle {K}}\right)&= \mathbf {P}\left( \mathcal {H}\left( \mathcal {T}^{*-}\right)>\overline{H}_n^{\scriptscriptstyle {K}}\right) -\mathbf {P}\left( \mathcal {H}\left( \mathcal {T}^{*-}\right) >\overline{H}_n^{\scriptscriptstyle {K}}+1\right) \\&\sim \Gamma \left( 2-\alpha \right) c_\mu ^{\alpha -1}\left( 1-\mu ^{\alpha -1}\right) \mathbf {P}\left( \xi ^*-1\ge \mu ^{-\overline{H}_n^{\scriptscriptstyle {K}}}\right) \\&= \Gamma \left( 2-\alpha \right) c_\mu ^{\alpha -1}\left( 1-\mu ^{\alpha -1}\right) \mathbf {P}\left( \xi ^*-1\ge c_\mu b_n^{\scriptscriptstyle {K}}\right) \end{aligned}$$

therefore

$$\begin{aligned} \frac{\mathbf {P}\left( \xi ^*-1\ge tb_n^{\scriptscriptstyle {K}}\right) }{\mathbf {P}\left( \overline{H}=\overline{H}_n^{\scriptscriptstyle {K}}\right) }&\sim \; \frac{\mathbf {P}\left( \xi ^*-1\ge tb_n^{\scriptscriptstyle {K}}\right) }{\Gamma (2-\alpha )\left( 1-\mu ^{\alpha -1}\right) c_\mu ^{\alpha -1} \mathbf {P}\left( \xi ^*-1\ge c_\mu b_n^{\scriptscriptstyle {K}}\right) } \;\\&\sim \; \frac{t^{-(\alpha -1)}}{\Gamma (2-\alpha )\left( 1-\mu ^{\alpha -1}\right) }. \end{aligned}$$

Combining this with (7.17) in (7.16) we have that

$$\begin{aligned} \lim _{n \rightarrow \infty }\mathbf {P}\left( \xi ^*-1\ge tb_n^{\scriptscriptstyle {K}}|\overline{H}=\overline{H}_n^{\scriptscriptstyle {K}}\right) =\frac{\alpha -1}{\Gamma (2-\alpha )\left( 1-\mu ^{\alpha -1}\right) }\int _t^\infty y^{-\alpha }\left( e^{-\mu y}-e^{-y}\right) \mathrm {d}y. \end{aligned}$$

$$\square $$

Notice that under $\mathbf {P}$ the pruned dummy branch $\mathcal {T}^*$ is the single vertex $\rho$ with high probability however under $\mathbf {P}^{\scriptscriptstyle {K}}$ there is at least one trap. By Lemma 5.1, conditional on *N*, $(W^j)_{j=1}^{N}$ have a joint negative multinomial distribution. Moreover, $W_j,B_j$ are coupled so that $B^j$ is binomially distributed with $W^j$ trials and success probability $p_1(H_j^+)$. The number $G^{j,k}$ of return times to $\delta ^j$ is geometrically distributed with failure probability $p_2(H_j^+)$. It follows that each $\tilde{\chi }^{i*}_n$ is equal in distribution to

$$\begin{aligned} \chi _n^*:=\sum _{j=1}^{N}\sum _{k=1}^{B^j}\sum _{l=1}^{G^{j,k}}\mathcal {R}^{j,k,l}. \end{aligned}$$

Define the scaled excursion time in large traps of a large branch as

7.18 $$\begin{aligned} \zeta ^{(n)}=\chi _n^*\beta ^{-\overline{H}} =\beta ^{-\overline{H}}\sum _{j=1}^{N}\sum _{k=1}^{B^j} \sum _{l=1}^{G^{j,k}}\mathcal {R}^{j,k,l} \end{aligned}$$

then we will show that $\zeta ^{(n)}$ converges in distribution under $\mathbb {P}^{\scriptscriptstyle {K}}$ along subsequences $n_l(t)$. Lemma 7.6 gives an upper bound on the number of large traps in a branch conditioned on its height.

### Lemma 7.6

For any $\epsilon >0$ and $K \in \mathbb {Z}$

$$\begin{aligned} \lim _{n \rightarrow \infty }\mathbf {P}^{\scriptscriptstyle {K}}\left( N\ge n^{\frac{\varepsilon +\epsilon }{\alpha -1}}\right) =0. \end{aligned}$$

### Proof

Conditioned on the height of the branch and number of buds we have that at least one trap attains the maximum height, all others have the distribution of heights of GW-tree conditioned on their maximum height therefore

7.19 $$\begin{aligned} \mathbf {P}^{\scriptscriptstyle {K}}\left( N\ge n^{\frac{\varepsilon +\epsilon }{\alpha -1}}\right)&\le \mathbf {P}^{\scriptscriptstyle {K}}\left( \xi ^*-1\ge \log (n)b_n^{\scriptscriptstyle {K}}\right) \nonumber \\&\quad + \mathbf {P}\left( N\ge n^{\frac{\varepsilon +\epsilon }{\alpha -1}} -1\big |\xi ^*-1=\log (n)b_n^{\scriptscriptstyle {K}}\right) . \end{aligned}$$

By Lemma 7.5 $\mathbf {P}^{\scriptscriptstyle {K}}\left( \xi ^*-1\ge \log (n)b_n^{\scriptscriptstyle {K}}\right)$ converges to 0 as $n \rightarrow \infty$. Conditioned on having $\xi ^*-1=\log (n)b_n^{\scriptscriptstyle {K}}$ buds we have that *N* is binomially distributed with $\log (n)b_n^{\scriptscriptstyle {K}}$ trails and success probability $\mathbf {P}(\mathcal {H}(\mathcal {T}^\circ )\ge h_{n,\varepsilon })\le C\mu ^{h_{n,\varepsilon }}$ by (3.1). Since for some slowly varying function $\overline{L}$ we have that

$$\begin{aligned} \mathbf {E}\left[ Bin\left( \log (n)b_n^{\scriptscriptstyle {K}}, \; C\mu ^{h_{n,\varepsilon }}\right) \right] \le C\mu ^{\scriptscriptstyle {K}}\log (n)\frac{a_n}{a_{n^{1-\varepsilon }}} \le \overline{L}(n)\mu ^{\scriptscriptstyle {K}}n^{\frac{\varepsilon }{\alpha -1}}, \end{aligned}$$

a Chernoff bound shows that the final term in (7.19) converges to 0. $\square$

For $$\tilde{\varepsilon }>0$$ write

$$\begin{aligned} A_9(n)=\bigcap _{j=1}^{N}\left\{ 1 \le \frac{\beta ^{H_j^+}}{1-\beta ^{-1}} E \left[ G^{j,1}\right] ^{-1} \le 1+\tilde{\varepsilon }\right\} . \end{aligned}$$

Recall from (7.7) that $p_2(H)$ is the probability that a walk started from the deepest point of a tree of height *H* reaches the root before returning to the deepest point. Since $G^{j,k}$ are independent geometric random variables there exist independent exponential random variables $e_{j,k}$ such that

$$\begin{aligned} G^{j,k}=\left\lfloor \frac{e_{j,k}}{-\log \left( 1-p_2(H_j^+)\right) }\right\rfloor \sim Geo\left( p_2(H_j^+)\right) . \end{aligned}$$

By (7.7) we then have that

7.20 $$\begin{aligned} E [G^{j,1}]=\left( 1-\frac{1-\beta ^{-1}}{\beta ^{H_j^+} -\beta ^{-1}}\right) \left( 1-\beta ^{-\left( H_j^++1\right) }\right) \frac{\beta ^{H_j^+}}{1-\beta ^{-1}} \end{aligned}$$

therefore, since $H_j^+\ge h_{n,\varepsilon }$, for any $\tilde{\varepsilon }>0$ there exists *n* large such that $\mathbf {P}^{\scriptscriptstyle {K}}(A_9(n))=1$ for any $K \in \mathbb {Z}$.

Recall from (7.7) and Definition 8 that $G^{j,k}$ is geometrically distributed with failure probability $p_2(H_j^+)\ge p_2(h_{n,\varepsilon })$. Write

$$\begin{aligned} A_{10}^{(j,k)}(n):=\left\{ \left( 1-\tilde{\varepsilon }\right) G^{j,k}\le E \left[ G^{j,k}\right] e_{j,k} \le \left( 1+\tilde{\varepsilon }\right) G^{j,k}\right\} . \end{aligned}$$

Then, using convergence of scaled geometric variables to exponential variables (see the proof of part (3) of Proposition 9.1 in [4]), we have that there exists a constant $\tilde{C}$ such that for any $\tilde{\varepsilon }>0$ there exists *n* large such that

$$\begin{aligned} P \left( A_{10}^{(j,k)}(n)^c\right) \le \tilde{C}p_2( h_{n,\varepsilon }) \le \tilde{C}a_{n^{1-\varepsilon }}^{-1/\gamma }. \end{aligned}$$

By Definition 8 we have that $B_j\le W_j$. Moreover $N\le n^{\frac{\varepsilon +\tilde{\epsilon }}{\alpha -1}}$ with high probability for any $\tilde{\epsilon }>0$ by Lemma 7.6 and $W^{j} \le C\log (n)$ for all *j* by the bound on the event $A_5(n)^c$ (from (5.3)). Therefore, writing

$$\begin{aligned} A_{10}(n):=\bigcap _{j=1}^{N}\bigcap _{k=1}^{B^{j}} A_{10}^{(j,k)}(n) \end{aligned}$$

a union bound gives us that $P (A_{10}(n)^c) \rightarrow 0$ as $n \rightarrow \infty$.

By comparison with the biased random walk on $\mathbb {Z}$ we have that $p_1(H_j^+)\ge p_\infty =1-\beta ^{-1}$ therefore we can define a random variable $B_\infty ^{j}\sim Bin(B^{j},p_\infty /p_1(H_j^+))$. It then follows that $B^{j}\ge B_\infty ^j \sim Bin(W^{j},p_\infty )$ and

7.21 $$\begin{aligned} p_1(H_j^+)-p_\infty = \frac{1-\beta ^{-1}}{1-\beta ^{-(H_j^++1)}}-(1-\beta ^{-1}) \le \beta ^{-H_j^+}. \end{aligned}$$

Write

$$\begin{aligned} A_{11}(n):=\bigcap _{j=1}^{N}\left\{ B^{j}= B_\infty ^{j}\right\} . \end{aligned}$$

Since the marginal distribution of $W^{1}$ doesn’t depend on *n*, using (7.21), the bound on *N* from Lemma 7.6 and the coupling between $B^{1}$ and $B_\infty ^{1}$ we have that

7.22 $$\begin{aligned} \mathbb {P}^{\scriptscriptstyle {K}}(A_{11}(n)^c)&\le o(1)+n^{\frac{\varepsilon +\tilde{\epsilon }}{\alpha -1}}\sum _{k=0}^\infty \mathbb {P}(W^{1}=k)\mathbb {P}(B^{1} \ne B_\infty ^{1}|W^{1}=k) \nonumber \\&\le o(1)+n^{\frac{\varepsilon +\tilde{\epsilon }}{\alpha -1}}\sum _{k=0}^\infty \mathbb {P}(W^{1}=k)k\left( p_1(H_1^+)-p_\infty \right) \nonumber \\&\le o(1) + n^{\frac{\varepsilon +\tilde{\epsilon }}{\alpha -1}}\beta ^{- h_{n,\varepsilon }}\mathbb {E}\left[ W^{1}\right] \end{aligned}$$

which decays to 0 as $n \rightarrow \infty$.

By choosing $\varepsilon >0$ sufficiently small we can choose $\kappa$ in the range $\varepsilon (1/\gamma +1/(\alpha -1))<\kappa < \min \{2(\alpha -1),\; 1/\gamma \}$ then write

$$\begin{aligned} A_{12}(n):=\bigcap _{j=1}^{N}\left\{ E \left[ (\mathcal {R}_n^{j,1,1})^2\right] < n^{\frac{\gamma ^{-1}-\kappa }{\alpha -1}}\right\} \end{aligned}$$

to be the event that there are no large traps with expected squared excursion time too large.

### Lemma 7.7

In IVIE, for any $K \in \mathbb {Z}$, as $n \rightarrow \infty$ we have that $\mathbb {P}^{\scriptscriptstyle {K}}(A_{12}(n)^c)\rightarrow 0$.

### Proof

Recall from (7.10) that, for $\epsilon >0$, $A_6(n)$ is the event that all large branches are shorter than $h_{n,\epsilon }^+$ and since $N\le n^{\frac{\varepsilon +\tilde{\epsilon }}{\alpha -1}}$ with high probability we have that

$$\begin{aligned} \mathbb {P}(A_{12}(n)^c) \le o(1)+n^{\frac{\varepsilon +\tilde{\epsilon }}{\alpha -1}} \mathbb {P}\left( \mathbf {1}_{\left\{ A_6(n)\right\} } E \left[ (\mathcal {R}_n^{1,1,1})^2\right] ^{1/2} >n^{\frac{\gamma ^{-1}-\kappa }{2(\alpha -1)}}\right) . \end{aligned}$$

A straightforward argument using conductances (see the proof of Lemma 9.1 in [4]) gives

$$\begin{aligned} E \left[ \left( \mathcal {R}_n^{1,1,1}\right) ^2\right] ^{1/2}\le C \sum _{y \in \mathcal {T}^+_1}\beta ^{d(y,\delta ^1)/2}\pi (y) \end{aligned}$$

where $\pi$ is the invariant measure scaled so that $\pi (\delta ^1)=1$ and *d* denotes the graph distance. We then have that

$$\begin{aligned} \mathbf {E}\left[ \mathbf {1}_{\left\{ A_6(n)\right\} } E \left[ \left( \mathcal {R}_n^{(1,1,1)}\right) ^2\right] ^{1/2}\right]&\le C \mathbf {E}\left[ \mathbf {1}_{\left\{ A_6(n)\right\} } \sum _{y \in \mathcal {T}^+_1}\beta ^{d\left( y,\delta ^1\right) /2}\pi (y)\right] \\&\le C \mathbf {E}\left[ \mathbf {1}_{\left\{ A_6(n)\right\} } \sum _{i\ge 1}\beta ^{i/2}\beta ^{-i}(1+\Lambda _i)\right] \\&\le C \sum _{i=0}^{ h_{n,\epsilon }^+}\left( \beta ^{1/2}\mu ^{\alpha -1-\epsilon }\right) ^i \end{aligned}$$

where the final inequality follows by (7.5). If $\beta ^{1/2}\mu ^{\alpha -1-\epsilon }\le 1$ then by Markov’s inequality we have that $\mathbb {P}^{\scriptscriptstyle {K}}(A_{12}(n)^c)\rightarrow 0$ as $n \rightarrow \infty$ since $\kappa <\gamma ^{-1}$. Otherwise by Markov’s inequality

$$\begin{aligned} \mathbb {P}^{\scriptscriptstyle {K}}(A_{12}(n)^c)\le & {} o(1)+Cn^{\frac{\varepsilon +\tilde{\epsilon }}{\alpha -1}} \left( \beta ^{1/2}\mu ^{\alpha -1-\epsilon }\right) ^{h_{n,\epsilon }^+} n^{\frac{\kappa -\gamma ^{-1}}{2(\alpha -1)}}\\\le & {} \overline{L}(n)n^{\frac{\kappa }{2(\alpha -1)} -1+\frac{\epsilon }{\alpha -1}\left( \frac{1}{2\gamma } +2-\alpha +\epsilon \right) +\frac{\varepsilon +\tilde{\epsilon }}{\alpha -1}} \end{aligned}$$

for some slowly varying function $\overline{L}$. In particular, since $\kappa <2(\alpha -1)$ we can choose $\epsilon ,\varepsilon ,\tilde{\epsilon }$ sufficiently small such that this converges to 0 as $n \rightarrow \infty$. $\square$

Write

$$\begin{aligned} A_{13}(n)= \bigcap _{j=1}^{N}\bigcap _{k=1}^{B^{j}} \bigg \{ (1-\tilde{\varepsilon })G^{j,k} E \left[ \mathcal {R}_n^{j,1,1}\right] \le \sum _{l=1}^{G^{j,k}}\mathcal {R}^{j,k,l} \le (1+\tilde{\varepsilon })G^{j,k} E \left[ \mathcal {R}_n^{j,1,1}\right] \bigg \} \end{aligned}$$

to be the event that on each excursion that reaches the deepest point of a large trap, the total excursion time before leaving the trap is approximately the product of the number of excursions and the expected excursion time.

### Lemma 7.8

In IVIE, for any $K \in \mathbb {Z}$, as $n \rightarrow \infty$ we have that $\mathbb {P}^{\scriptscriptstyle {K}}(A_{13}(n)^c)\rightarrow 0$.

### Proof

With high probability we have that no trap is visited more than $C\log (n)$ by (5.3) and also $N\le n^{\frac{\varepsilon +\tilde{\epsilon }}{\alpha -1}}$ by Lemma 7.6. Any excursion is of length at least 2 hence $E [\mathcal {R}_n^{1,1,1}]\ge 2$. Therefore, by Lemma 7.7 and Chebyshev’s inequality

$$\begin{aligned}&\mathbb {P}^{\scriptscriptstyle {K}}(A_{13}(n)^c) \le o(1)\\&\qquad +C\log (n)n^{\frac{\varepsilon +\tilde{\epsilon }}{\alpha -1}} \mathbb {P}\left( \left| \sum _{l=1}^{G^{1,1}}\frac{\mathcal {R}_n^{1,1,l}}{ E \left[ \mathcal {R}_n^{1,1,1}\right] G^{1,1}}-1\right|> \tilde{\varepsilon }, G^{1,1}>0, E \left[ \left( \mathcal {R}_n^{1,1,1}\right) ^2\right] <n^{\frac{\gamma ^{-1}-\kappa }{\alpha -1}}\right) \\&\quad \le o(1)+\frac{C\log (n)n^{\frac{\gamma ^{-1} +\varepsilon +\tilde{\epsilon }-\kappa }{\alpha -1}}}{\tilde{\varepsilon }^2} E \left[ \frac{\mathbf {1}_{\left\{ G^{1,1}>0\right\} }}{G^{1,1}}\right] . \end{aligned}$$

It then follows that since $G^{1,1} \sim Geo(p_2(H_1^+))$ (where from (7.7) $p_2(H)$ is the probability that a walk reaches the deepest point in the trap of height *H*) and $p_2(H_1^+)\le c\beta ^{-h_{n,\varepsilon }}=ca_{n^{1-\varepsilon }}^{-\frac{1}{\gamma }}$

$$\begin{aligned} E \left[ \frac{\mathbf {1}_{\left\{ G^{(1,1,1)}>0\right\} }}{G^{(1,1,1)}}\right] \; \le \; E \left[ -\frac{p_2\left( H_1^+\right) }{1-p_2\left( H_1^+\right) } \log \left( p_2\left( H_1^+\right) \right) \right] \; \le \; \overline{L}(n)n^{-\frac{1-\varepsilon }{\gamma (\alpha -1)}} \end{aligned}$$

for some slowly varying function $\overline{L}$. In particular, $\mathbb {P}^{\scriptscriptstyle {K}}(A_{13}(n)^c) \le o(1)+L_{\tilde{\varepsilon }}(n)n^{\frac{\varepsilon \left( \frac{1}{\gamma }+\frac{1}{\alpha -1}\right) +\tilde{\epsilon }-\kappa }{\alpha -1}}$ which converges to zero by the choice of $\kappa > \varepsilon (1/\gamma +1/(\alpha -1))$. $\square$

Lemma 7.9 illustrates that the expected time spent on an excursion from the deepest point of a trap of height at least $h_{n,\varepsilon }$ doesn’t differ too greatly from the expected excursion time in an infinite version of the trap. Let $\mathcal {R}^j_\infty$ be an excursion time from $\delta ^j$ to itself in an extension of $\mathcal {T}^+_j$ to an infinite trap constructed according to the algorithm at the beginning of the section where $\mathcal {T}^\prec _{H_j^+}$ is replaced by $\mathcal {T}^+_j$. Write

$$\begin{aligned} A_{14}(n):=\bigcap _{j=1}^{N}\left\{ E \left[ \mathcal {R}_\infty ^j\right] - E \left[ \mathcal {R}^{j,k,l}\right] <\tilde{\varepsilon }\right\} . \end{aligned}$$

### Lemma 7.9

In IVIE, for any $K\in \mathbb {Z}$ as $n \rightarrow \infty$ we have that $\mathbf {P}^{\scriptscriptstyle {K}}(A_{14}(n)^c)\rightarrow 0$.

### Proof

A straightforward computation similar to that in Proposition 9.1 of [4] yields that for some constant *c* and *n* sufficiently large

$$\begin{aligned} 0\le E \left[ \mathcal {R}_\infty ^j\right] - E \left[ \mathcal {R}^{j,k,l}\right] \le c\beta ^{- h_{n,\varepsilon }/2}\sum _{k=0}^{ h_{n,\varepsilon }/2} \beta ^{-k}(1+\Lambda _k)+2\sum _{k= h_{n,\varepsilon }/2+1}^\infty \beta ^{-k}(1+\Lambda _k) \end{aligned}$$

for all $j=1,\ldots ,N$ where $\Lambda _k$ are the weights of the extension of $\mathcal {T}^+_j$. Recall that $N\le n^{\frac{\varepsilon +\tilde{\epsilon }}{\alpha -1}}$ with high probability by Lemma 7.6, therefore by (7.5) and Markov’s inequality

$$\begin{aligned} \mathbf {P}(A_{14}(n)^c)&\le \frac{Cn^{\frac{\varepsilon +\tilde{\epsilon }}{\alpha -1}}}{\tilde{\varepsilon }}\mathbf {E}\left[ E \left[ \mathcal {R}_\infty ^j\right] - E \left[ \mathcal {R}_n^{j,1,1}\right] \right] \\&\le C_{\tilde{\varepsilon }}n^{\frac{\varepsilon +\tilde{\epsilon }}{\alpha -1}}\left( \beta ^{-\frac{h_{n,\varepsilon }}{2}}\sum _{k=0}^\infty \left( \beta ^{-k}+\mu ^{k\left( \alpha -1-\tilde{\varepsilon }\right) }\right) +\sum _{k=h_{n,\varepsilon }/2+1}^\infty \mu ^{k\left( \alpha -1-\tilde{\varepsilon }\right) }\right) \\&\le C_{\tilde{\varepsilon }}n^{\frac{\varepsilon +\tilde{\epsilon }}{\alpha -1}}\left( \beta ^{-\frac{h_{n,\varepsilon }}{2}} +\mu ^{h_{n,\varepsilon }\frac{\left( \alpha -1-\tilde{\varepsilon }\right) }{2}}\right) . \end{aligned}$$

Since we can choose $\tilde{\varepsilon },\varepsilon$ and $\tilde{\epsilon }$ arbitrarily small we indeed have the desired result. $\square$

Define

7.23 $$\begin{aligned} Z_\infty ^n:=\frac{1}{1-\beta ^{-1}}\sum _{j=1}^{N} \beta ^{H_j^+-\overline{H}} E \left[ \mathcal {R}_\infty ^j\right] \sum _{k=1}^{B_\infty ^j}e_{j,k} \end{aligned}$$

whose distribution depends on *n* only through *N* and $(H_j^+-\overline{H})_{j= 1}^{N}$. Recalling the definition of $\zeta ^{(n)}$ in (7.18), since $e_{j,k}$ are the exponential random variables defining $G^{j,k}$, $B_\infty ^j \sim Bin(B^j,p_\infty /p_1(H_1^+))$ and the random variable *N* is the same in both equations, we have that $\zeta ^{(n)}$ and $Z_\infty ^n$ are defined on the same probability space.

### Proposition 7.10

In IVIE, for any $K \in \mathbb {Z}$ and $\tilde{\epsilon }>0$

$$\begin{aligned} \lim _{n \rightarrow \infty }\mathbb {P}^{\scriptscriptstyle {K}}\left( \left| \zeta ^{(n)}-Z_\infty ^n\right| >\tilde{\epsilon }\right) =0. \end{aligned}$$

### Proof

Using the bounds on $A_{11}, A_{13}$ and $A_{14}$ from (7.22) and Lemmas 7.8 and 7.9 respectively there exists some function $g:\mathbb {R}\rightarrow \mathbb {R}$ such that $\lim _{\tilde{\varepsilon } \rightarrow 0^+}g(\tilde{\varepsilon })=0$ and for sufficiently large *n* (independently of *K*)

$$\begin{aligned} \mathbb {P}^{\scriptscriptstyle {K}}\left( \left| \zeta ^{(n)}-Z_\infty ^n\right|>\tilde{\epsilon }\right) \le o(1) + 2\mathbb {P}^{\scriptscriptstyle {K}}\left( g(\tilde{\varepsilon })Z_\infty ^n >\tilde{\epsilon }\right) . \end{aligned}$$

It therefore suffices to show that $(Z_\infty ^n)_{n\ge 0}$ are tight under $\mathbb {P}^K$. Write

7.24 $$\begin{aligned} S_j:=\frac{1}{1-\beta ^{-1}} E \left[ \mathcal {R}_\infty ^j\right] \sum _{k=1}^{B_\infty ^{j}}e_{j,k}. \end{aligned}$$

The variables $E [\mathcal {R}^j_\infty ]$, $B_\infty ^j$ and $e_{j,k}$ are independent, don’t depend on *K* and have finite mean (by Lemma 7.1, the geometric distribution of $W^j$ and exponential distribution of $e^{j,k}$) therefore

7.25 $$\begin{aligned} \mathbb {E}^{\scriptscriptstyle {K}}\left[ S_j\right] \le C<\infty \end{aligned}$$

uniformly over *K*. We can then write

$$\begin{aligned} Z_\infty ^n=\sum _{j=1}^{N}\beta ^{H_j^+ -\overline{H}_n^{\scriptscriptstyle {K}}}S_j. \end{aligned}$$

The distribution of $S_j$ is independent of the height of the trap. The number of large traps *N* is dominated by the total number of traps $\xi ^*-1$ in the branch thus reintroducing small traps

7.26 $$\begin{aligned} \mathbb {P}^{\scriptscriptstyle {K}}\left( Z_\infty ^n\ge t\right) \le \mathbb {P}^{\scriptscriptstyle {K}} \left( \sum _{j=1}^{b_n^{\scriptscriptstyle {K}} \log (t)}\beta ^{H_j-\overline{H}_n^{\scriptscriptstyle {K}}}S_j \ge t\right) +\mathbb {P}^{\scriptscriptstyle {K}}\left( \xi ^*-1\ge b_n^{\scriptscriptstyle {K}}\log (t)\right) \end{aligned}$$

where we recall that, under $\mathbb {P}^{\scriptscriptstyle {K}}$, $(H_j)_{j=1}^{\xi ^*-1}$ are distributed as the heights of independent *f*-GW-trees conditioned so that the largest is of height $\overline{H}_n^{\scriptscriptstyle {K}}$ and $(S_j)_{j=1}^{\xi ^*-1}$ are i.i.d. with the law of $S_1$. By Lemma 7.5 we have that $\lim _{t\rightarrow \infty }\limsup _{n \rightarrow \infty }\mathbb {P}^{\scriptscriptstyle {K}}(\xi ^*-1\ge b_n^{\scriptscriptstyle {K}}\log (t))=0$ therefore it remains to bound the first term in (7.26).

Write $\Phi =\inf \{r\ge 1:H_r=\overline{H}_n^{\scriptscriptstyle {K}}\}$ to be the index of the first trap with height the same as the maximum in the branch. Conditional on trap *j* being the first in the branch which attains the maximum height we have that the heights of the remaining traps are independent and either at most the height of the largest (for higher indices than *j*) or strictly shorter (for lower indices than *j*). In particular, this means that

$$\begin{aligned}&\mathbb {P}^{\scriptscriptstyle {K}}\left( \sum _{j=1}^{b_n^{\scriptscriptstyle {K}} \log (t)}\beta ^{H_j-\overline{H}_n^{\scriptscriptstyle {K}}}S_j \ge t\right) \le \mathbb {P}^{\scriptscriptstyle {K}}\left( \sum _{j=1}^{b_n^{\scriptscriptstyle {K}} \log (t)}\beta ^{H_j-\overline{H}_n^{\scriptscriptstyle {K}}}S_j \ge t\Big |\Phi =1\right) \\&\quad \le \mathbb {P}\left( S_1\ge \log (t)\right) + \mathbb {P}\left( \sum _{j=2}^{b_n^{\scriptscriptstyle {K}}\log (t)}\beta ^{H_j -\overline{H}_n^{\scriptscriptstyle {K}}}S_j \ge t-\log (t)\Big | H_j\le \overline{H}_n^{\scriptscriptstyle {K}} \; \forall j\ge 2 \right) . \end{aligned}$$

The distribution of $S_1$ is independent of *n* therefore $\lim _{t\rightarrow \infty }\mathbb {P}(S_1\ge \log (t))= 0$. Conditional on $\Phi =1$, $(H_j)_{j\ge 2}$ are independent therefore by Markov’s inequality we have that

$$\begin{aligned}&\mathbb {P}\left( \sum _{j=2}^{b_n^{\scriptscriptstyle {K}} \log (t)}\beta ^{H_j-\overline{H}_n^{\scriptscriptstyle {K}}}S_j \ge t-\log (t)\Big |\bigcap _{j\ge 2} H_j\le \overline{H}_n^{\scriptscriptstyle {K}} \right) \\&\quad \le \frac{b_n^{\scriptscriptstyle {K}}\log (t)\mathbb {E}\left[ S_1\right] \mathbb {E}\left[ \beta ^{H_1}|H_1\le \overline{H}_n^{\scriptscriptstyle {K}}\right] }{\beta ^{\overline{H}_n^{\scriptscriptstyle {K}}}(t-\log (t))}. \end{aligned}$$

For large enough *n* we have that $\mathbb {P}(H_1\le \overline{H}_n^{\scriptscriptstyle {K}})\ge 1/2$ therefore we have that

$$\begin{aligned} \mathbb {P}\left( H_1=l\big |H_1\le \overline{H}_n^{\scriptscriptstyle {K}}\right) \le \mathbb {P}\left( H_1\ge l\big |H_1\le \overline{H}_n^{\scriptscriptstyle {K}}\right) \le \frac{1}{2}\mathbb {P}\left( H_1\ge l\right) \le C\mu ^l \end{aligned}$$

for some constant *C* therefore the result follows from

7.27 $$\begin{aligned} \mathbb {E}\left[ \beta ^{H_1}\big |H_1\le \overline{H}_n^{\scriptscriptstyle {K}}\right] \; = \; \sum _{l=0}^{\overline{H}_n^{\scriptscriptstyle {K}}}\beta ^l \mathbb {P}\left( H_1=l\big |H_1\le \overline{H}_n^{\scriptscriptstyle {K}}\right) \; \le \; C (\beta \mu )^{\overline{H}_n^{\scriptscriptstyle {K}}}. \end{aligned}$$

$$\square $$

We now prove three technical lemmas which will be important in the proof of Proposition 7.14 which is the main result of the section. The first shows that we can reintroduce the small traps into $Z_\infty ^n$. The reason for doing this is that we no longer need to condition on the heights of the traps being at least the critical level which will simplify later calculations. In particular, we can replace *N* with $\xi ^*-1$ (i.e. the total number of traps in the branch) which we understand under $\mathbf {P}^{\scriptscriptstyle {K}}$ by Lemma 7.5.

### Lemma 7.11

For all $\tilde{\varepsilon }>0$ we have that for any $K \in \mathbb {Z}$ as $n \rightarrow \infty$,

$$\begin{aligned} \mathbb {P}^{\scriptscriptstyle {K}}\left( \sum _{j=1}^{\xi ^*-1-N} \beta ^{H_j^--\overline{H}_n^{\scriptscriptstyle {K}}}S_j>\tilde{\varepsilon } \right) \rightarrow 0. \end{aligned}$$

### Proof

First, notice that each term in the sum is nonnegative therefore introducing extra terms only increases the probability. By Lemma 7.5, for any $\tilde{\epsilon }>0$, we have that $\mathbb {P}^{\scriptscriptstyle {K}}(\xi ^*-1\ge a_{n^{1+\tilde{\epsilon }}})\rightarrow 0$ as $n \rightarrow \infty$. We therefore have that

$$\begin{aligned} \mathbb {P}^{\scriptscriptstyle {K}}\left( \sum _{j=1}^{\xi ^*-1-N} \beta ^{H_j^--\overline{H}_n^{\scriptscriptstyle {K}}}S_j>\tilde{\varepsilon } \right)&\le \mathbb {P}\left( \sum _{j=1}^{a_{n^{1+\tilde{\epsilon }}}} \beta ^{H_j-\overline{H}_n^{\scriptscriptstyle {K}}}S_j >\tilde{\varepsilon }\Big | H_j<h_{n,\varepsilon } \; \forall j \ge 1 \right) \\&\quad +o(1). \end{aligned}$$

By Definitions 7 and 8 we have that $\beta ^{\overline{H}_n^{\scriptscriptstyle {K}}}\le \beta ^{\scriptscriptstyle {K}}a_n^{1/\gamma }$ therefore by Markov’s inequality and (7.27) we have that

$$\begin{aligned} \mathbb {P}\left( \sum _{j=1}^{a_{n^{1+\tilde{\epsilon }}}}\beta ^{H_j -\overline{H}_n^{\scriptscriptstyle {K}}}S_j>\tilde{\varepsilon } \Big | H_j<h_{n,\varepsilon } \; \forall j \ge 1 \right)&\le \; \frac{a_{n^{1+\tilde{\epsilon }}}\mathbb {E}\left[ S_1\right] \mathbb {E}\left[ \beta ^{H_1}\big | H_1<h_{n,\varepsilon }\right] }{\tilde{\varepsilon } \beta ^{\overline{H}_n^{\scriptscriptstyle {K}}}}\\ \quad&\le \frac{C_{\scriptscriptstyle {K},\tilde{\varepsilon }} a_{n^{1+\tilde{\epsilon }}}(\beta \mu )^{ h_{n,\varepsilon }}}{a_n^{1/\gamma }}. \end{aligned}$$

Recall from Definition 7 that $h_{n,\varepsilon }\le \log (a_{n^{1-\varepsilon }})/\log (\mu ^{-1})$ therefore

$$\begin{aligned} (\beta \mu )^{h_{n,\varepsilon }}\le a_{n^{1-\varepsilon }}^{\frac{1}{\gamma }-1}. \end{aligned}$$

Using the form of $a_n$ following Definition 3 we then have that there exists a slowly varying function $\overline{L}$ such that

$$\begin{aligned} \frac{a_{n^{1+\tilde{\epsilon }}}(\beta \mu )^{ h_{n,\varepsilon }}}{a_n^{1/\gamma }} \le \overline{L}(n)n^{\frac{1}{\alpha -1} \left( \tilde{\epsilon }+\varepsilon -\frac{\varepsilon }{\gamma }\right) } \end{aligned}$$

which converges to 0 by choosing $\tilde{\epsilon }<\varepsilon (1/\gamma -1)$. $\square$

The second Lemma leading to Proposition 7.14 shows that the height of an *f*-GW-tree is sufficiently close to a geometric random variable. To ease notation let $S=S_1$ (see (7.24)), $H=H_1\sim \mathcal {H}(\mathcal {T}^\circ )$ be distributed as the height of a GW-tree and $G \sim Geo(\mu )$ independently of each other.

### Lemma 7.12

In IVIE,

7.28 $$\begin{aligned} b\left| \int _0^\infty e^{-x}\mathbb {P}\left( S\beta ^H\ge \frac{xb^{1/\gamma }}{\theta }\right) \mathrm {d}x -c_\mu \int _0^\infty e^{-x}\mathbb {P}\left( S\beta ^G\ge \frac{xb^{1/\gamma }}{\theta }\right) \mathrm {d}x\right| \end{aligned}$$

converges to zero as $b\rightarrow \infty$.

### Proof

From (1.1) and (7.25) we have that $\gamma <1$ and $\mathbb {E}[S]<\infty$ therefore $\mathbb {E}[S^\gamma ]<\infty$. By independence of *S* and *G*

$$\begin{aligned} \mathbb {P}\left( S\beta ^G\ge \frac{xb^{1/\gamma }}{\theta }\right)&=\mathbb {E}\left[ \mathbb {P}\left( G\ge \frac{\log \left( xb^{1/\gamma }(S\theta )^{-1}\right) }{\log (\beta )}\Big |S\right) \right] \\&\le \left( \frac{xb^{1/\gamma }}{\theta }\right) ^{-\gamma } \mathbb {E}\left[ S^\gamma \right] =\frac{C_\theta }{bx^\gamma }. \end{aligned}$$

Similarly, since there exist a constant *c* such that $\mathbb {P}(H\ge t)\le c\mathbb {P}(G\ge t)$ uniformly over *t* we have that

$$\begin{aligned} \mathbb {P}\left( S\beta ^H\ge \frac{xb^{1/\gamma }}{\theta }\right) \le \frac{C_\theta }{bx^\gamma }. \end{aligned}$$

Let $\tilde{\varepsilon }>0$ then choose $\epsilon >0$ such that

$$\begin{aligned} \int _0^\epsilon e^{-x}x^{-\gamma }\mathrm {d}x < \frac{\tilde{\varepsilon }}{C_\theta } \end{aligned}$$

then, since the integrals are positive and $c_\mu \le 1$, we have that

7.29 $$\begin{aligned} \left| \int _0^\epsilon e^{-x}c_\mu \mathbb {P}\left( S\beta ^G\ge \frac{xb^{1/\gamma }}{\theta }\right) \mathrm {d}x-\int _0^\epsilon e^{-x}\mathbb {P}\left( S\beta ^H\ge \frac{xb^{1/\gamma }}{\theta }\right) \mathrm {d}x\right| \le \tilde{\varepsilon }b^{-1}. \end{aligned}$$

By (3.1) we have that

7.30 $$\begin{aligned} m(b):=\sup _{z>\frac{\epsilon b}{\theta }}\left| \frac{\mathbb {P}\left( \beta ^H\ge z\right) }{\mathbb {P}\left( \beta ^G\ge z\right) }-c_\mu \right| \end{aligned}$$

converges to 0 as $b\rightarrow \infty$. Now define $M(b):=m(b)^{1-\frac{1}{\gamma }} \wedge b^{\frac{1}{\gamma }-1}$ then $M(b)\rightarrow \infty$ as $b\rightarrow \infty$ but $M(b)<< b^{1/\gamma }$.

For $x>\epsilon$, by independence of *S* and *H* we have that

$$\begin{aligned} \mathbb {P}\left( S\beta ^H\ge \frac{xb^{1/\gamma }}{\theta }\Big |S\ge M(b)\right)&\le \; C\mathbb {E}\left[ \left( \frac{xb^{1/\gamma }}{\theta S}\right) ^{\frac{\log (\mu )}{\log (\beta )}}\Big |S\ge M(b)\right] \\&\le \; C_{\epsilon ,\theta }b^{-1}\mathbb {E}\left[ S^\gamma \big |S\ge M(b)\right] . \end{aligned}$$

In particular,

$$\begin{aligned} b\mathbb {P}\left( S\beta ^H\ge \frac{xb^{1/\gamma }}{\theta }, \; S\ge M(b)\right) \le C_{\epsilon ,\theta }\mathbb {E}\left[ S^\gamma \mathbf {1}_{\left\{ S\ge M(b)\right\} }\right] \end{aligned}$$

which converges to 0 as $b\rightarrow \infty$ by dominated convergence. Similarly, the same holds replacing *H* with *G* therefore combining this with (7.29) we have that the quantity (7.28) is bounded above by

$$\begin{aligned} \tilde{\varepsilon }+o(1)+Cb\sup _{x>\epsilon } \left| \mathbb {P}\left( S\beta ^H\ge \frac{xb^{1/\gamma }}{\theta }, \; S<M(b)\right) -c_\mu \mathbb {P}\left( S\beta ^G\ge \frac{xb^{1/\gamma }}{\theta }, \; S<M(b)\right) \right| . \end{aligned}$$

Since *S* is independent of *G* and *H* we have that the supremum in the above expression can be bounded above by

$$\begin{aligned} \sup _{z>\frac{\epsilon b^{1/\gamma }}{\theta M(b)}}\left| \mathbb {P}\left( \beta ^H\ge z\right) -c_\mu \mathbb {P}\left( \beta ^G\ge z\right) \right| \le m(b)\mathbb {P}\left( G\ge \frac{\log \left( \epsilon b^{1/\gamma } (\theta M(b))^{-1}\right) }{\log (\beta )}\right) \end{aligned}$$

by (7.30) since $b^{1/\gamma }/M(b)\ge b$. Since $G\sim Geo(\mu )$ we have that

$$\begin{aligned} m(b)\mathbb {P}\left( G\ge \frac{\log (\epsilon b^{1/\gamma } (\theta M(b))^{-1})}{\log (\beta )}\right) = C_{\epsilon ,\theta }m(b)\left( \frac{b^{1/\gamma }}{M(b)}\right) ^{\frac{\log (\mu )}{\log (\beta )}} \le \frac{C_{\epsilon ,\theta } m(b)^\gamma }{b} \end{aligned}$$

which completes the proof. $\square$

In the final Lemma preceding Proposition 7.14 we show that the Laplace transform

$$\begin{aligned} \varphi _{\scriptscriptstyle {K}}(\lambda )&:=\mathbb {E}^{\scriptscriptstyle {K}}\left[ e^{-\lambda \sum _{j=1}^{\xi ^*-1} \beta ^{H_j-\overline{H}_n^{\scriptscriptstyle {K}}}S_j}\right] \end{aligned}$$

can be written in terms of the distributions of *S*, *H* and $\xi ^*$.

### Lemma 7.13

In IVIE,

$$\begin{aligned} \varphi _{\scriptscriptstyle {K}}(\lambda ) =\mathbb {E}^{\scriptscriptstyle {K}}\left[ \frac{\mathbb {E}\left[ e^{-\lambda S\beta ^{H-\overline{H}_n^{\scriptscriptstyle {K}}}}\mathbf {1}_{\left\{ H\le \overline{H}_n^{\scriptscriptstyle {K}}\right\} }\right] ^{\xi ^*-1} -\mathbb {E}\left[ e^{-\lambda S\beta ^{H-\overline{H}_n^{\scriptscriptstyle {K}}}}\mathbf {1}_{\{H\le \overline{H}_n^{\scriptscriptstyle {K}}-1\}}\right] ^{\xi ^*-1}}{\mathbf {P}\left( H\le \overline{H}_n^{\scriptscriptstyle {K}}\right) ^{\xi ^*-1}-\mathbf {P}\left( H\le \overline{H}_n^{\scriptscriptstyle {K}}-1\right) ^{\xi ^*-1}}\right] \end{aligned}$$

### Proof

Recall that $\Phi :=\inf \{r\ge 1:H_r=\overline{H}\}$ is the index of the first random variable in the sequence $(H_j)_{j=1}^{\xi ^*-1}$ which attains the maximum value $\overline{H}:=\max _{j\le \xi ^*-1}H_j$. For $h \in \mathbb {Z}^+, \lambda >0$ and $i=1,2$ write

7.31 $$\begin{aligned} \psi _i(h,\lambda ) \;&:= \; \mathbb {E}\left[ e^{-\lambda S\beta ^{H-h}}\big |H\le h+1-i\right] , \nonumber \\ \phi _i(h,\lambda ) \;&:= \; \mathbb {E}\left[ e^{-\lambda S\beta ^{H-h}}\mathbf {1}_{\left\{ H\le h+1-i\right\} }\right] \; = \; \psi _i(h,\lambda )\mathbb {P}(H\le h+1-i). \end{aligned}$$

Conditional on $\Phi$, the random variables $(H_j)_{j\ge 1}$ are independent with

$$\begin{aligned} \mathbb {P}^{\scriptscriptstyle {K}}\left( H_j=z|\Phi \right) \; = \; {\left\{ \begin{array}{ll} \mathbf {1}_{\left\{ z=\overline{H}_n^{\scriptscriptstyle {K}}\right\} }, &{} \text { if } j=\Phi , \\ \mathbb {P}\left( H=z\big |H\le \overline{H}_n^{\scriptscriptstyle {K}}-1\right) , &{} \text { if } j<\Phi , \\ \mathbb {P}\left( H=z\big |H\le \overline{H}_n^{\scriptscriptstyle {K}}\right) , &{} \text { if } j>\Phi . \end{array}\right. } \end{aligned}$$

By conditioning on $\xi ^*$, we then have that

7.32 $$\begin{aligned} \varphi _{\scriptscriptstyle {K}}(\lambda )&=\mathbb {E}^{\scriptscriptstyle {K}}\left[ \sum _{k=1}^{\xi ^*-1} \mathbf {P}^{\scriptscriptstyle {K}}(\Phi =k|\xi ^*) \mathbb {E}^{\scriptscriptstyle {K}}\left[ e^{-\lambda \sum _{j=1}^{\xi ^*-1} \beta ^{H_j-\overline{H}_n^{\scriptscriptstyle {K}}}S_j}\Big | \Phi =k,\xi ^*\right] \right] \nonumber \\&= \mathbb {E}^{\scriptscriptstyle {K}}\left[ \mathbb {E}\left[ e^{-\lambda S}\right] \sum _{k=1}^{\xi ^*-1}\mathbf {P}^{\scriptscriptstyle {K}}(\Phi =k|\xi ^*) \psi _2\left( \overline{H}_n^{\scriptscriptstyle {K}},\lambda \right) ^{k-1} \psi _1\left( \overline{H}_n^{\scriptscriptstyle {K}},\lambda \right) ^{\xi ^*-1-k}\right] \end{aligned}$$

and by Bayes’ rule we also have that

7.33 $$\begin{aligned} \mathbf {P}^{\scriptscriptstyle {K}}(\Phi =k|\xi ^*)&= \frac{\mathbf {P}\left( H=\overline{H}_n^{\scriptscriptstyle {K}}\right) }{\mathbf {P}\left( \overline{H}=\overline{H}_n^{\scriptscriptstyle {K}}\big |\xi ^*\right) } \mathbf {P}\left( H\le \overline{H}_n^{\scriptscriptstyle {K}}-1\right) ^{k-1} \mathbf {P}\left( H\le \overline{H}_n^{\scriptscriptstyle {K}}\right) ^{\xi ^*-1-k}. \end{aligned}$$

Combining (7.31), (7.32) and (7.33) we can then write $\varphi _{\scriptscriptstyle {K}}(\lambda )$ as

7.34 $$\begin{aligned} \mathbb {E}^{\scriptscriptstyle {K}}\left[ \frac{\mathbb {E}\left[ e^{-\lambda S}\right] \mathbf {P}\left( H=\overline{H}_n^{\scriptscriptstyle {K}}\right) }{\mathbf {P}\left( \overline{H}=\overline{H}_n^{\scriptscriptstyle {K}}\big |\xi ^*\right) } \sum _{k=1}^{\xi ^*-1} \phi _2\left( \overline{H}_n^{\scriptscriptstyle {K}},\lambda \right) ^{k-1} \phi _1\left( \overline{H}_n^{\scriptscriptstyle {K}},\lambda \right) ^{\xi ^*-1-k}\right] . \end{aligned}$$

For $$0<p<q<1$$ and $$l \in \mathbb {Z}^+$$,

$$\begin{aligned} \sum _{k=1}^lp^{k-1}q^{l-k} \; = \; q^{l-1}\sum _{k=0}^{l-1}\left( \frac{p}{q}\right) ^k \; = \; q^{l-1}\left( \frac{1-\left( \frac{p}{q}\right) ^l}{1-\frac{p}{q}}\right) \; = \; \frac{q^l-p^l}{q-p}. \end{aligned}$$

Since $0<\phi _2(\overline{H}_n^{\scriptscriptstyle {K}},\lambda )^{k-1}<\phi _1(\overline{H}_n^{\scriptscriptstyle {K}},\lambda )^{k-1}<1$, by (7.34) it follows that $\varphi _{\scriptscriptstyle {K}}(\lambda )$ is equal to

$$\begin{aligned} \mathbb {E}^{\scriptscriptstyle {K}}\left[ \frac{\mathbb {E}\left[ e^{-\lambda S}\right] \mathbf {P}\left( H=\overline{H}_n^{\scriptscriptstyle {K}}\right) }{\mathbf {P}\left( \overline{H}=\overline{H}_n^{\scriptscriptstyle {K}} \big |\xi ^*\right) } \left( \frac{\phi _1\left( \overline{H}_n^{\scriptscriptstyle {K}},\lambda \right) ^{\xi ^*-1} -\phi _2\left( \overline{H}_n^{\scriptscriptstyle {K}},\lambda \right) ^{\xi ^*-1}}{\phi _1\left( \overline{H}_n^{\scriptscriptstyle {K}},\lambda \right) -\phi _2\left( \overline{H}_n^{\scriptscriptstyle {K}},\lambda \right) }\right) \right] \end{aligned}$$

however, from (7.31),

$$\begin{aligned} \phi _1\left( \overline{H}_n^{\scriptscriptstyle {K}},\lambda \right) -\phi _2\left( \overline{H}_n^{\scriptscriptstyle {K}},\lambda \right)&= \mathbb {E}\left[ e^{-\lambda S}\right] \mathbf {P}\left( H=\overline{H}_n^{\scriptscriptstyle {K}}\right) . \end{aligned}$$

therefore this is equal to

$$\begin{aligned} \mathbb {E}^{\scriptscriptstyle {K}}\left[ \frac{\phi _1 \left( \overline{H}_n^{\scriptscriptstyle {K}},\lambda \right) ^{\xi ^*-1} -\phi _2\left( \overline{H}_n^{\scriptscriptstyle {K}},\lambda \right) ^{\xi ^*-1}}{\mathbf {P}\left( \overline{H}=\overline{H}_n^{\scriptscriptstyle {K}}\big |\xi ^*\right) }\right] \end{aligned}$$

The result then follows from

$$\begin{aligned} \mathbf {P}\left( \overline{H}=\overline{H}_n^{\scriptscriptstyle {K}}\big |\xi ^*\right)&= \mathbf {P}\left( \overline{H}\le \overline{H}_n^{\scriptscriptstyle {K}}\big |\xi ^*\right) -\mathbf {P}\left( \overline{H}\le \overline{H}_n^{\scriptscriptstyle {K}}-1\big |\xi ^*\right) \\&= \mathbf {P}\left( H\le \overline{H}_n^{\scriptscriptstyle {K}}\right) ^{\xi ^*-1}-\mathbf {P}\left( H\le \overline{H}_n^{\scriptscriptstyle {K}}-1\right) ^{\xi ^*-1} \end{aligned}$$

which is a consequence of $\overline{H}$ being the maximum of $\xi ^*-1$ i.i.d. random variables. $\square$

The next proposition shows that, under $\mathbb {P}^{\scriptscriptstyle {K}}$, we have that the scaled time spent in a large branch $\zeta ^{(n)}$ (from (7.18)) converges in distribution along subsequences $n_l$ where $a_{n_l(t)}\sim t\mu ^{-l}$.

### Proposition 7.14

In IVIE, under $\mathbb {P}^{\scriptscriptstyle {K}}$ we have that $Z_\infty ^{n_l}$ converges in distribution (as $l \rightarrow \infty$) to some random variable $Z_\infty$.

### Proof

By Lemmas 7.11 and 7.13, it now suffices to show convergence of

7.35 $$\begin{aligned} \varphi _{\scriptscriptstyle {K}}(\lambda ) =\mathbb {E}^{\scriptscriptstyle {K}}\left[ \frac{\mathbb {E}\left[ e^{-\lambda S\beta ^{H-\overline{H}_n^{\scriptscriptstyle {K}}}}\mathbf {1}_{\left\{ H\le \overline{H}_n^{\scriptscriptstyle {K}}\right\} }\right] ^{\xi ^*-1} -\mathbb {E}\left[ e^{-\lambda S\beta ^{H-\overline{H}_n^{\scriptscriptstyle {K}}}}\mathbf {1}_{\left\{ H\le \overline{H}_n^{\scriptscriptstyle {K}}-1\right\} }\right] ^{\xi ^*-1}}{\mathbf {P}\left( H\le \overline{H}_n^{\scriptscriptstyle {K}}\right) ^{\xi ^*-1}-\mathbf {P}\left( H\le \overline{H}_n^{\scriptscriptstyle {K}}-1\right) ^{\xi ^*-1}}\right] . \end{aligned}$$

By (3.2) we have that $\mathbf {P}(H\le \overline{H}_n^{\scriptscriptstyle {K}})=1-c_\mu \mu ^{1+\overline{H}_n^{\scriptscriptstyle {K}}}(1+o(1))$ therefore, using the relationship (7.15) between $b_n^{\scriptscriptstyle {K}}$ and $\overline{H}_n^{\scriptscriptstyle {K}}$ we have that

$$\begin{aligned} \mathbf {P}\left( H\le \overline{H}_n^{\scriptscriptstyle {K}}\right) ^{\xi ^*-1} \; = \; \left( 1-\frac{\mu (1+o(1))}{b_n^{\scriptscriptstyle {K}}}\right) ^{\xi ^*-1} \;=\;\exp \left( -\frac{\xi ^*-1}{b_n^{\scriptscriptstyle {K}}} \mu \left( 1+o(1)\right) \right) \end{aligned}$$

and similarly,

$$\begin{aligned} \mathbf {P}\left( H\le \overline{H}_n^{\scriptscriptstyle {K}}-1\right) ^{\xi ^*-1} \; = \; \exp \left( -\frac{\xi ^*-1}{b_n^{\scriptscriptstyle {K}}}\left( 1+o(1)\right) \right) . \end{aligned}$$

By Lemma 7.5 we know that $(\xi ^*-1)/b_n^{\scriptscriptstyle {K}}$ converges in distribution to a random variable with exponential moments therefore we want to show a similar expression for the numerator in (7.35). Notice that

7.36 $$\begin{aligned}&\mathbb {E}\left[ e^{-\lambda S\beta ^{H-\overline{H}_n^{\scriptscriptstyle {K}}}}\mathbf {1}_{\left\{ H \le \overline{H}_n^{\scriptscriptstyle {K}}\right\} }\right] ^{\xi ^*-1}\nonumber \\&\quad = \mathbb {E}\left[ e^{-\lambda S\beta ^{H-\overline{H}_n^{\scriptscriptstyle {K}}}}\right] ^{\xi ^*-1} \left( 1-\frac{ \mathbb {E}\left[ e^{-\lambda S\beta ^{H-\overline{H}_n^{\scriptscriptstyle {K}}}}\mathbf {1}_{\left\{ H > \overline{H}_n^{\scriptscriptstyle {K}}\right\} }\right] }{ \mathbb {E}\left[ e^{-\lambda S\beta ^{H-\overline{H}_n^{\scriptscriptstyle {K}}}}\right] }\right) ^{\xi ^*-1} \end{aligned}$$

where $\mathbb {E}\left[ e^{-\lambda S\beta ^{H-\overline{H}_n^{\scriptscriptstyle {K}}}}\right]$ converges to 1 deterministically. In particular, this means that

7.37 $$\begin{aligned}&\left( 1-\frac{ \mathbb {E}\left[ e^{-\lambda S\beta ^{H-\overline{H}_n^{\scriptscriptstyle {K}}}}\mathbf {1}_{\left\{ H> \overline{H}_n^{\scriptscriptstyle {K}}\right\} }\right] }{ \mathbb {E}\left[ e^{-\lambda S\beta ^{H-\overline{H}_n^{\scriptscriptstyle {K}}}}\right] }\right) ^{\xi ^*-1}\nonumber \\&\quad = \exp \left( -(\xi ^*-1)\mathbb {E}\left[ e^{-\lambda S\beta ^{H-\overline{H}_n^{\scriptscriptstyle {K}}}}\mathbf {1}_{\left\{ H > \overline{H}_n^{\scriptscriptstyle {K}}\right\} }\right] (1+o(1))\right) . \end{aligned}$$

By summing over the possible values of *H* and using independence of *S* and *H* we have that

$$\begin{aligned} \mathbb {E}\left[ e^{-\lambda S\beta ^{H-\overline{H}_n^{\scriptscriptstyle {K}}}}\mathbf {1}_{\left\{ H > \overline{H}_n^{\scriptscriptstyle {K}}\right\} }\right]&= (1+o(1))\sum _{j=\overline{H}_n^{\scriptscriptstyle {K}}+1}^\infty (1-\mu )\mu ^j \mathbb {E}\left[ e^{-\lambda S\beta ^{j-\overline{H}_n^{\scriptscriptstyle {K}}}}\right] \\&= (1+o(1))\mu ^{\overline{H}_n^{\scriptscriptstyle {K}}+1}\sum _{j=0}^\infty (1-\mu )\mu ^j \mathbb {E}\left[ e^{-\lambda S\beta ^{j+1}}\right] . \end{aligned}$$

Recalling $G\sim Geo(\mu )$ independently of *S* then writing $\varphi ^{SG}(\lambda )$ to be the Laplace transform of $S\beta ^G$ and using the relationship (7.15) between $b_n^{\scriptscriptstyle {K}}$ and $\overline{H}_n^{\scriptscriptstyle {K}}$ we therefore have that (7.37) can be written as

7.38 $$\begin{aligned} \exp \left( -\frac{\xi ^*-1}{b_n^{\scriptscriptstyle {K}}} \mu \varphi ^{SH}(\lambda \beta )(1+o(1))\right) . \end{aligned}$$

It remains to deal with $\mathbb {E}[e^{-\lambda S\beta ^{H-\overline{H}_n^{\scriptscriptstyle {K}}}}]^{b_n^{\scriptscriptstyle {K}}}$. To ease notation, let us write $b:=b_n^{\scriptscriptstyle {K}}=c_\mu ^{-1}\mu ^{-\overline{H}_n^{\scriptscriptstyle {K}}}$ and $\theta =\lambda c_\mu ^{-1/\gamma }$ then

7.39 $$\begin{aligned} \mathbb {E}\left[ e^{-\lambda S\beta ^{H-\overline{H}_n^{\scriptscriptstyle {K}}}} \right] ^{b_n^{\scriptscriptstyle {K}}}&=\mathbb {E}\left[ e^{-\theta S\beta ^Hb^{-1/\gamma }}\right] ^b \nonumber \\&= \left( \int _0^1 \mathbb {P}\left( e^{-\theta S\beta ^Hb^{-1/\gamma }}\ge y\right) \mathrm {d}y\right) ^b \nonumber \\&= \left( 1-\int _0^1 \mathbb {P}\left( S\beta ^H\ge -\frac{\log (y)b^{1/\gamma }}{\theta }\right) \mathrm {d}y\right) ^b \nonumber \\&= \left( 1-\int _0^\infty e^{-x} \mathbb {P}\left( S\beta ^H\ge \frac{xb^{1/\gamma }}{\theta }\right) \mathrm {d}x\right) ^b \nonumber \\&= \left( 1-\int _0^\infty e^{-x} c_\mu \mathbb {P}\left( S\beta ^G\ge \frac{xb^{1/\gamma }}{\theta }\right) \mathrm {d}x\right) ^b +o(1) \end{aligned}$$

where the final equality holds by Lemma 7.12. Since *S* and *G* are independent we have that

$$\begin{aligned} \mathbb {P}\left( S\beta ^G\ge z\right) \; = \; \mathbb {E}\left[ \mathbb {P}\left( G\ge \frac{\log (z/S)}{\log (\beta )}\Big |S\right) \right] \; = \; \mathbb {E}\left[ \mu ^{\left\lceil \frac{\log (z/S)}{\log (\beta )}\right\rceil }\right] . \end{aligned}$$

Writing

$$\begin{aligned} J(z):=\left\lceil \frac{\log (z)}{\log (\beta )}\right\rceil -\frac{\log (z)}{\log (\beta )} \quad \text {and}\quad I(z):=\mathbb {E}\left[ S^{\gamma }\mu ^{-\left\lfloor \frac{\log (S)}{\log (\beta )} +J(z)\right\rfloor + \frac{\log (S)}{\log (\beta )} +J(z) }\right] \end{aligned}$$

we then have that

$$\begin{aligned} \mathbb {P}(S\beta ^G\ge z) \; = \; \mu ^\frac{\log (z)}{\log (\beta )} \mathbb {E}\left[ S^{\gamma }\mu ^{-\left\lfloor \frac{\log (S)}{\log (\beta )} +J(z)\right\rfloor + \frac{\log (S)}{\log (\beta )} +J(z) }\right] \;= \;z^{-\gamma }I(z) \end{aligned}$$

where, from (7.25), we also have that $I(z)\le \mathbb {E}[S^\gamma ]<\infty$ since $\gamma <1$ by (1.1). Moreover, $J(z)=J(zm^{\log (\beta )})$ and $I(z)=I(zm^{\log (\beta )})$ for all $z \in \mathbb {R}, \; m \in \mathbb {Z}$.

Substituting this back into (7.39) we have that

$$\begin{aligned} \mathbb {E}\left[ e^{-\theta S\beta ^Gb^{-1/\gamma }}\right] ^b \; = \;\left( 1-\theta ^\gamma b^{-1}\int _0^\infty e^{-x} x^{-\gamma }I\left( \frac{xb^{1/\gamma }}{\theta } \right) \mathrm {d}x\right) ^b+o(1). \end{aligned}$$

For $t>0$, along sequences $n_l(t)$ such that $a_{n_l(t)}\sim t\mu ^{-l}$ we have that $(b_n^{\scriptscriptstyle {K}})^{1/\gamma } \sim Cl^{\log (\beta )}$ therefore, since *I* is bounded, we have that along subsequences $n_l(t)$

$$\begin{aligned} \int _0^\infty e^{-x} x^{-\gamma }I\left( \frac{xb^{1/\gamma }}{\theta }\right) \mathrm {d}x \end{aligned}$$

converges to some positive function of $\theta$. In particular, we have that

$$\begin{aligned} \mathbb {E}\left[ e^{-\lambda S\beta ^{H-\overline{H}_n^{\scriptscriptstyle {K}}}} \right] ^{b_n^{\scriptscriptstyle {K}}} \end{aligned}$$

converges to some constant in the interval (0, 1). Combining this with (7.36) and (7.38) we have that

$$\begin{aligned} \mathbb {E}\left[ e^{-\lambda S\beta ^{H-\overline{H}_n^{\scriptscriptstyle {K}}}}\mathbf {1}_{\left\{ H \le \overline{H}_n^{\scriptscriptstyle {K}}\right\} }\right] ^{\xi ^*-1} =\exp \left( -\frac{\xi ^*-1}{b_n^{\scriptscriptstyle {K}}}\mu C_{\lambda ,\beta }(1+o(1))\right) \end{aligned}$$

for some constant $C_{\mu ,\beta }$ depending on the distribution of *S*. Furthermore, the same arguments gives us that

$$\begin{aligned} \mathbb {E}\left[ e^{-\lambda S\beta ^{H-\overline{H}_n^{\scriptscriptstyle {K}}}}\mathbf {1}_{\left\{ H \le \overline{H}_n^{\scriptscriptstyle {K}}-1\right\} } \right] ^{\xi ^*-1}=\exp \left( -\frac{\xi ^*-1}{b_n^{\scriptscriptstyle {K}}} C_{\lambda ,\beta }(1+o(1))\right) . \end{aligned}$$

By boundedness, continuity and Lemma 7.5 we therefore have that $\varphi _{\scriptscriptstyle {K}}(\lambda )$ converges along the given subsequences which proves the result. $\square$

In order to prove the convergence result for sums of i.i.d. variables we shall require that $\zeta ^{(n)}$ can be dominated (independently of $K\ge h_{n,\varepsilon }- h_{n,0}$) by some random variable $Z_{sup}$ such that $\mathbb {E}[Z_{sup}^{(\alpha -1)\gamma +\epsilon }]<\infty$ for $\epsilon$ sufficiently small. Lemma 7.15 shows that we indeed have the domination required for the sums of i.i.d. variables result.

### Lemma 7.15

In IVIE, there exists a random variable $Z_{sup}$ such that under $\mathbb {P}^{\scriptscriptstyle {K}}$ for any $K \in \mathbb {Z}$ we have that $Z_{sup} \succeq \zeta ^{(n)}$ for all *n* sufficiently large and $\mathbb {E}[Z_{sup}^{1-\epsilon }]<\infty$ for any $\epsilon >0$.

### Proof

The number of large traps *N* is dominated by the number of traps in the branch. Similarly to Lemma 7.5 we consider

$$\begin{aligned} \mathbf {P}\left( \xi ^*-1\ge tb_n^{\scriptscriptstyle {K}}\big |\overline{H} =\overline{H}_n^{\scriptscriptstyle {K}}\right) =\mathbf {P}\left( \overline{H}=\overline{H}_n^{\scriptscriptstyle {K}}\big |\xi ^*-1\ge tb_n^{\scriptscriptstyle {K}}\right) \frac{\mathbf {P}\left( \xi ^*-1\ge tb_n^{\scriptscriptstyle {K}}\right) }{\mathbf {P}\left( \overline{H}=\overline{H}_n^{\scriptscriptstyle {K}}\right) }. \end{aligned}$$

Using the tail of *H* from (3.1), for large *n* (independently of $t\ge 0$) and some constant *c*, we can bound $\mathbf {P}(\overline{H}=\overline{H}_n^{\scriptscriptstyle {K}}|\xi ^*-1\ge tb_n^{\scriptscriptstyle {K}})$ above by

$$\begin{aligned} \mathbf {E}\left[ \mathbf {P}\left( H\le \overline{H}_n^{\scriptscriptstyle {K}}\right) ^{\xi ^*-1} \Big |\xi ^*-1\ge tb_n^{\scriptscriptstyle {K}}\right] \; \le \; \mathbf {E}\left[ e^{-c\left( \frac{\xi ^*-1}{b_n^{\scriptscriptstyle {K}}}\right) } \Big |\xi ^*-1\ge tb_n^{\scriptscriptstyle {K}}\right] \; \le \; e^{-ct}. \end{aligned}$$

For each $t\ge 0$ we have that $\mathbf {P}(\xi ^*-1\ge tb_n^{\scriptscriptstyle {K}})\sim Ct^{-(\alpha -1)}\mathbf {P}(\overline{H}=\overline{H}_n^{\scriptscriptstyle {K}})$ as $n \rightarrow \infty$. Since $\mathbf {P}(\overline{H}=\overline{H}_n^{\scriptscriptstyle {K}})$ doesn’t depend on *t* we can choose a constant *c* such that for *n* sufficiently large we have that $\mathbf {P}(\overline{H}=\overline{H}_n^{\scriptscriptstyle {K}}) \le c\mathbf {P}(\xi ^*-1\ge b_n^{\scriptscriptstyle {K}})$ thus for $t\ge 1$

$$\begin{aligned} \frac{\mathbf {P}\left( \xi ^*-1\ge tb_n^{\scriptscriptstyle {K}}\right) }{\mathbf {P}\left( \overline{H}=\overline{H}_n^{\scriptscriptstyle {K}}\right) } \le \frac{\mathbf {P}\left( \xi ^*-1\ge tb_n^{\scriptscriptstyle {K}}\right) }{c\mathbf {P}\left( \xi ^*-1\ge b_n^{\scriptscriptstyle {K}}\right) } \le c^{-1}. \end{aligned}$$

In particular, for $t\ge 1$ we have that $\mathbf {P}(\xi ^*-1\ge tb_n^{\scriptscriptstyle {K}}|\overline{H}=\overline{H}_n^{\scriptscriptstyle {K}}) \le c_1e^{-c_2t}$ for some constants $c_1,c_2$. It follows that there exists some random variable $\xi _{sup}$ which is independent of $\overline{H}$, has an exponential tail and satisfies $\xi _{sup}b_n^{\scriptscriptstyle {K}}\ge \xi ^*-1$ on the event $\{\overline{H}=\overline{H}_n^{\scriptscriptstyle {K}}\}$ for *n* suitably large (independently of *K*).

Recall that the total number of excursions $W^j$ in a trap exceeds the number which reach the deepest point $B^j$ and we write $G^{j,k}$ to denote the number of excursions from the deepest point. The length of these excursions can be dominated by excursions $\mathcal {R}_\infty ^{j,k,l}$ from the deepest points of the infinite traps $\mathcal {T}^\prec _i$. We then have that for *n* suitably large, under $\mathbb {P}^{\scriptscriptstyle {K}}$

$$\begin{aligned} \zeta ^{(n)} \preceq \sum _{j=1}^{\xi _{sup}b_n^{\scriptscriptstyle {K}}} \sum _{k=1}^{W^{j}}\sum _{l=1}^{G^{j,k}} \frac{\mathcal {R}_\infty ^{j,k,l}}{\beta ^{\overline{H}_n^{\scriptscriptstyle {K}}}}. \end{aligned}$$

By (7.20) $E [G^{j,k}] \le \beta ^{H_j+1}/(\beta +1)$ therefore there is some constant *c* such that, writing

$$\begin{aligned} \mathscr {Y}_j^{(n)}:= c\sum _{k=1}^{W^j}\sum _{l=1}^{G^{j,k}}\frac{\mathcal {R}_\infty ^{j,k,l}}{ E [G^{j,k}]} \end{aligned}$$

(which are identically distributed under $\mathbb {P}$) we have that under $\mathbb {P}^{\scriptscriptstyle {K}}$,

$$\begin{aligned} \zeta ^{(n)} \preceq \frac{1}{\beta ^{\overline{H}_n^{\scriptscriptstyle {K}}}} \sum _{j=1}^{\xi _{sup}b_n^{\scriptscriptstyle {K}}}\beta ^{H_j} \mathscr {Y}_j^{(n)}. \end{aligned}$$

For $m\ge 1$ write $\mathscr {X}^n(m):=\frac{1}{m}\sum _{j=1}^m \beta ^{H_j}\mathscr {Y}_j^{(n)}\mathbf {1}_{\{j\ne \Phi \}}$ (where we recall that $\Phi$ is the first index *j* such that $H_j=\overline{H}_n^{\scriptscriptstyle {K}}$) then by Markov’s inequality

$$\begin{aligned} \mathbb {P}^{\scriptscriptstyle {K}}\left( \mathscr {X}^n(m)\ge t\right)&\le \; \frac{1}{m}\sum _{j=1}^m\frac{\mathbb {E}^{\scriptscriptstyle {K}} \left[ \beta ^{H_j}\mathscr {Y}_j^{(n)}\mathbf {1}_{\left\{ j\ne \Phi \right\} }\right] }{t}\\&= \; \frac{1}{m}\sum _{j=1}^m\frac{\mathbb {E}^{\scriptscriptstyle {K}} \left[ \beta ^{H_j}\mathbf {1}_{\left\{ j\ne \Phi \right\} }\right] \mathbb {E}^{\scriptscriptstyle {K}}\left[ \mathscr {Y}_j^{(n)}\right] }{t} \end{aligned}$$

since $\mathbb {E}[\mathscr {Y}_j^{(n)}|H_j, \Phi ]$ is independent of $H_j$ and $\Phi$. Since $W^1$ has a geometric distribution (independently of *n*) we have that $\mathbb {E}[W^1]<\infty$ and by Lemma 7.1 we have that $\mathbb {E}[\mathcal {R}_\infty ]<\infty$ therefore $\mathbb {E}^{\scriptscriptstyle {K}}[\mathscr {Y}_j^{(n)}]\le \mathbb {E}[W^1]\mathbb {E}[\mathcal {R}_\infty ]\le C<\infty$ for all *n*. Using geometric bounds on the tail of *H* from (3.1) and that $\mathbb {P}(H\ge j|H \le \overline{H}_n^{\scriptscriptstyle {K}})\le \mathbb {P}(H\ge j)$ we have that

$$\begin{aligned} \mathbb {E}^{\scriptscriptstyle {K}}\left[ \beta ^{H}\right] \; \le \; \sum _{j=0}^{\infty } \beta ^j\mathbb {P}\left( H\ge j\big |H \le \overline{H}_n^{\scriptscriptstyle {K}}\right) \; \le \; C(\beta \mu )^{\overline{H}_n^{\scriptscriptstyle {K}}}. \end{aligned}$$

We therefore have that $\mathbb {P}^{\scriptscriptstyle {K}}(\mathscr {X}^n(m)\ge t) \le C(\beta \mu )^{\overline{H}_n^{\scriptscriptstyle {K}}}/t$ thus there exists some sequence of random variables $\mathscr {X}_{sup}^n \succeq \mathscr {X}^n(m)$ for any *m* such that $\mathbb {P}^{\scriptscriptstyle {K}}(\mathscr {X}_{sup}^n\ge t)=1 \wedge C(\beta \mu )^{\overline{H}_n^{\scriptscriptstyle {K}}}t^{-1}$. In particular, $\mathscr {X}_{sup}^n \succeq \mathscr {X}^n(\xi _{sup}b_n^{\scriptscriptstyle {K}})$. Therefore,

$$\begin{aligned} \frac{1}{\beta ^{\overline{H}_n^{\scriptscriptstyle {K}}}} \sum _{j=1}^{\xi _{sup}b_n^{\scriptscriptstyle {K}}} \beta ^{H_j}\mathscr {Y}_j^{(n)}= \frac{\xi _{sup}b_n^{\scriptscriptstyle {K}}}{\beta ^{\overline{H}_n^{\scriptscriptstyle {K}}}}\mathscr {X}^n(\xi _{sup}b_n^{\scriptscriptstyle {K}})+\mathscr {Y}_\Phi ^{(n)} \preceq \frac{\xi _{sup}\mathscr {X}_{sup}^n}{c_\mu (\beta \mu )^{\overline{H}_n^{\scriptscriptstyle {K}}}}+\mathscr {Y}_\Phi ^{(n)} \end{aligned}$$

under $\mathbb {P}^{\scriptscriptstyle {K}}$. We then have that

$$\begin{aligned} \mathbb {P}^{\scriptscriptstyle {K}}\left( \frac{\xi _{sup} \mathscr {X}_{sup}^n}{c_\mu (\beta \mu )^{\overline{H}_n^{\scriptscriptstyle {K}}}}\ge t\right)&= \mathbb {E}^{\scriptscriptstyle {K}} \left[ \mathbb {P}^{\scriptscriptstyle {K}}\left( \mathscr {X}_{sup}^n\ge \frac{tc_\mu (\beta \mu )^{\overline{H}_n^{\scriptscriptstyle {K}}}}{\xi _{sup}}\Big | \xi _{sup}\right) \right] =1 \wedge C\frac{\mathbb {E}^{\scriptscriptstyle {K}}\left[ \xi _{sup}\right] }{t} \end{aligned}$$

where $\xi _{sup}$ has finite first moment since $\mathbf {P}(\xi _{sup}\ge t)=c_1e^{-c_2t} \wedge 1$. It follows that there exists $\mathscr {X}_{sup}\succeq \mathscr {X}_{sup}^n$ for any *n* such that $\mathbb {P}(\mathscr {X}_{sup}\ge t)=1\wedge Ct^{-1}$.

Since $\mathbb {E}^{\scriptscriptstyle {K}}[\mathscr {Y}_\Phi ^n]$ is bounded independently of *K* and *n*, by Markov’s inequality we have that there exists $\mathscr {Y}_{sup}\succeq \mathscr {Y}_\Phi ^n$ for all *n* such that $\mathbb {P}(\mathscr {Y}_{sup}\ge t)=1\wedge Ct^{-1}$. It therefore follows that $\zeta ^{(n)}$ under $\mathbb {P}^{\scriptscriptstyle {K}}$ is stochastically dominated by $\mathscr {X}_{sup}+\mathscr {Y}_{sup}$ under $\mathbb {P}$ where

$$\begin{aligned} \mathbb {P}\left( \mathscr {X}_{sup}+\mathscr {Y}_{sup}\ge t\right) \; \le \; \mathbb {P}\left( \mathscr {X}_{sup}\ge t/2\right) + \mathbb {P}\left( \mathscr {Y}_{sup}\ge t/2\right) \; \le \; Ct^{-1} \end{aligned}$$

hence $\mathscr {X}_{sup}+\mathscr {Y}_{sup}$ has finite moments up to $1-\epsilon$ for all $\epsilon >0$. $\square$

## Convergence along subsequences

In this section we prove the main theorems concerning convergence to infinitely divisible laws in FVIE and IVIE. Both cases follow the proof from [4]; in FVIE the result follows directly whereas in IVIE adjustments need to be made to deal with slowly varying functions.

Recall that we want to show convergence of $\Delta _{n}/a_n$ along sequences $n_l(t)$ however by Corollary 5.5 and Lemma 5.6 it suffices to consider

$$\begin{aligned} \tilde{\chi }_{t,n}=\sum _{i=1}^{\left\lfloor ntq_n \right\rfloor }\tilde{\chi }_n^i \end{aligned}$$

where $\tilde{\chi }_n^i$ is the time spent in large traps of the $i{\text {th}}$ large branch by walk $X_n^{(i)}$. Furthermore, by Proposition 7.3 and Corollary 7.4 we can replace $\tilde{\chi }_n^i$ with $\tilde{\chi }_n^{i*}$ which is the time spent on excursions from the deepest point of the traps of the $i{\text {th}}$ branch by $X_n^{(i)}$.

Let $\overline{H}_i$ denote the height of the largest trap in the $i{\text {th}}$ large branch then for $i,l\ge 1$ let $\zeta _i^l:=\tilde{\chi }_{n_l}^{i*}\beta ^{-\overline{H}_i}$ then $(\zeta _i^l)_{i\ge 1}$ are i.i.d. with the law of $\zeta ^{(n_l)}$. Let $n_l:=n_l(1)$ then for $K \ge -(l-h_{n_l,\varepsilon })$ let $\zeta _i^{l,K}$ be $\zeta _i^l$ conditioned on the event $\{\overline{H}_i=l+K\}$ when this makes sense and 0 otherwise. For $K \in \mathbb {Z}$ and $l\ge 0$ define $\overline{F}_{\scriptscriptstyle {K}}^l(x):=\mathbb {P}(\zeta _i^{l,K}>x)$.

### Proof of Theorem 2 (FVIE)

Recall that in FVIE $\gamma =\log (\mu ^{-1})/\log (\beta )<1$, $n_l(t)=\lfloor t\mu ^{-l}\rfloor$ and by Corollary 3.2 we have that the height of a branch decays exponentially: $\mathbf {P}(\mathcal {H}(\mathcal {T}^{*-})\ge n) \sim C_\mathcal {D}\mu ^n = C_\mathcal {D}\beta ^{-n\gamma }$ where $C_\mathcal {D}=c_\mu \mathbf {E}[\xi ^*-1]$.

By a simple adaptation of Corollary 7.10 and Lemma 7.15

1. $\exists Z_\infty ^{(i)}$ random variables such that for all $K \in \mathbb {Z}$ we have that $\zeta _i^{l,K} \mathop {\rightarrow }\limits ^{{\mathrm{d}}}Z_\infty ^{(i)}$ as $l \rightarrow \infty$;
2. $\exists Z_{sup}$ random variable such that for all $l \ge 0$ and $K \ge -(l-h_{n_l,\varepsilon })$ we have that $\zeta _i^{l,K}\preceq Z_{sup}$ and $\mathbf {E}[Z_{sup}^{\gamma +\epsilon }]<\infty$ for some $\epsilon >0$.

More specifically, since there is precisely one large trap in a large branch with high probability in FVIE the random variable in (7.23) can be written as

$$\begin{aligned} Z_\infty ^n=\frac{1}{1-\beta ^{-1}} E \left[ \mathcal {R}_\infty \right] \sum _{k=1}^{B_\infty }e_k \end{aligned}$$

for some binomial variable $B_\infty$ and independent exponential variables $e_k$. These are independent of *n*, hence an adaptation of Proposition 7.10 shows that $\zeta ^{(n)}$ converge in distribution under $\mathbb {P}^{\scriptscriptstyle {K}}$.

Set

$$\begin{aligned} S_M^l := \sum _{i=1}^M \tilde{\chi }_{n_l}^{i*}. \end{aligned}$$

For $(\lambda _l)_{l\ge 0}$ converging to $\lambda >0$ define $M_l^\lambda :=\lfloor \lambda _l^\gamma \beta ^{\gamma (l-h_{n_l,\varepsilon })}\rfloor$ and $K_l^\lambda :=\lambda \beta ^l$ then denote $\overline{F}_\infty (x):=\mathbf {P}(Z_\infty >x)$. Theorem 7 is Theorem 10.1 of [4].

#### Theorem 7

Suppose $\gamma <1$ and properties 1 and 2 hold then

$$\begin{aligned} S_{M_l^\lambda }^l/K_l^\lambda \mathop {\rightarrow }\limits ^{{\mathrm{d}}}R_{d_\lambda ,0,\mathcal {L}_\lambda } \end{aligned}$$

where $R_{d_\lambda ,0,\mathcal {L}_\lambda }$ has an infinitely divisible law with drift

$$\begin{aligned} d_\lambda =\lambda ^{1+\gamma }\left( 1-\beta ^{-\gamma }\right) \sum _{K \in \mathbb {Z}}\beta ^{(1+\gamma )K}\mathbf {E}\left[ \frac{Z_\infty }{(\lambda \beta ^K)^2 +(Z_\infty )^2}\right] , \end{aligned}$$

0 variance and Lévy spectral function $\mathcal {L}_\lambda$ satisfying $\mathcal {L}_\lambda (x)=\lambda ^\gamma \mathcal {L}_1(\lambda x)$ for all $\lambda >0, x \in \mathbb {R}$ with $\mathcal {L}_1(x)=0$ for $x<0$ and

$$\begin{aligned} \mathcal {L}_1(x)=-\left( 1-\beta ^{-\gamma }\right) \sum _{K \in \mathbb {Z}} \beta ^{K\gamma } \overline{F}_\infty (x\beta ^{K}) \end{aligned}$$

for $$x\ge 0$$.

Combining this with the remark at the beginning of the section with $\lambda =(tC_\mathcal {D})^{1/\gamma }=(tc_\mu \mathbf {E}[\xi ^*-1])^{1/\gamma }$ and that eventually $l=h_{n_l,0}$ we have that

$$\begin{aligned} \frac{\Delta _{n_l(t)}}{(C_\mathcal {D}n_l(t))^\frac{1}{\gamma }} \mathop {\rightarrow }\limits ^{{\mathrm{d}}}R_{d_{(tC_\mathcal {D})^{1/\gamma }},0,\mathcal {L}_{(tC_\mathcal {D})^{1/\gamma }}} \end{aligned}$$

which proves Theorem 2.

### Proof of Theorem 3 (IVIE)

In IVIE write $\gamma _\alpha :=(\alpha -1)\log (\mu ^{-1})/\log (\beta )=(\alpha -1)\gamma$. By (3.12) we have that

$$\begin{aligned} \mathbf {P}\left( \mathcal {H}(\mathcal {T}^{*-})> n\right) \sim c_\mu ^{\alpha -1}\Gamma (2-\alpha )\mathbf {P}\left( \xi ^* \ge \mu ^{-n}\right) \sim C_{\mu ,\alpha } \beta ^{-\gamma _\alpha n} L\left( \beta ^{\gamma _\alpha n}\right) \end{aligned}$$

for a known constant $C_{\mu ,\alpha }$. Due to the slowly varying term, we cannot apply Theorem 7 directly however Theorem 7 is proved using Theorem 6. It will therefore suffice to show convergence of the drift, variance and Lévy spectral function in this case.

Recall that we consider subsequences $n_l(t)$ such that $a_{n_l(t)} \sim t\mu ^{-l}$. From Propositions 7.10 and 7.14 we then have that for any $K \in \mathbb {Z}$ the laws of $\zeta _i^{l,K}$ converge to the laws of $Z_\infty$ as $l \rightarrow \infty$. Let $(Z_\infty ^{(i)})_{i\ge 1}$ be an independent sequence of variables with this law and denote $\overline{F}_\infty (x):=\mathbb {P}(Z_\infty >x)$. By Lemma 7.15, $\exists Z_{sup}$ such that $\zeta _i^{l,K} \preceq Z_{sup}$ for all $l \in \mathbb {N}, K \ge -(l-h_{n_l,\varepsilon })$ and $\mathbf {E}[Z_{sup}^{\gamma _\alpha +\epsilon }]<\infty$ for some $\epsilon >0$; we denote $\overline{F}_{sup}(x):=\mathbf {P}(Z_{sup}>x)$. For $(\lambda _l)_{l\ge 0}$ converging to $\lambda >0$ define $K_l^\lambda :=\lambda \beta ^{l}$ and for $C_{\alpha ,\mu }=\mu ^{-1}(2-\alpha )/(\alpha -1)$

$$\begin{aligned} M_l^\lambda :=\left\lfloor \lambda _l^{\gamma _\alpha } \beta ^{\gamma _\alpha l} \frac{\mathbf {P}\left( \xi ^*>\mu ^{-h_{n_l,\varepsilon }}\right) }{C_{\alpha ,\mu }L\left( \mu ^{-h_{n_l,0}}\right) }\right\rfloor . \end{aligned}$$

#### Proposition 8.1

In IVIE, for any $\lambda >0$, as $l \rightarrow \infty$

$$\begin{aligned} \sum _{i=1}^{M_l^\lambda }\frac{\tilde{\chi }_{n_l}^{i*}}{K_l^\lambda } \mathop {\rightarrow }\limits ^{{\mathrm{d}}}R_{d_\lambda ,0,\mathcal {L}_\lambda } \end{aligned}$$

where

$$\begin{aligned} d_\lambda&= \lambda ^{1+\gamma _\alpha }(1-\beta ^{-\gamma _\alpha })\sum _{K \in \mathbb {Z}}\beta ^{(1+\gamma _\alpha )K}\mathbb {E}\left[ \frac{Z_\infty }{(\lambda \beta ^{K})^2 +(Z_\infty )^2}\right] ,\\ \mathcal {L}_\lambda (x)&= {\left\{ \begin{array}{ll} 0 &{} x\le 0; \\ -\lambda ^{\gamma _\alpha }(1-\beta ^{-\gamma _\alpha }) \sum _{K \in \mathbb {Z}}\beta ^{K\gamma _\alpha }\overline{F}_\infty (\lambda x \beta ^{K\gamma _\alpha }) &{} x>0. \end{array}\right. } \end{aligned}$$

#### Proof

By Theorem 6 it suffices to show the following:

1. for all $$\epsilon >0$$
  $$\begin{aligned} \lim _{l\rightarrow \infty } \;\mathbb {P}\left( \frac{\tilde{\chi }_{n_l}^{1*}}{K_l^\lambda }>\epsilon \right) =0; \end{aligned}$$
2. for all *x* continuity points
  $$\begin{aligned} \mathcal {L}_\lambda (x)= {\left\{ \begin{array}{ll} 0 &{} x\le 0, \\ -\lim _{l\rightarrow \infty }\; M_l^\lambda \mathbb {P}\left( \frac{\tilde{\chi }_{n_l}^{1*}}{K_l^\lambda }>x \right) &{} x>0; \end{array}\right. } \end{aligned}$$
3. for all $\tau >0$ continuity points of $\mathcal {L}$
  $$\begin{aligned} d_\lambda= & {} \lim _{l \rightarrow \infty } \;M_l^\lambda \mathbb {E}\left[ \frac{\tilde{\chi }_{n_l}^{1*}}{K_l^\lambda } \mathbf {1}_{\left\{ \tilde{\chi }_{n_l}^{1*}\le \tau K_l^\lambda \right\} }\right] +\int _{\left| x\right| \ge \tau }\frac{x}{1+x^2}\mathrm {d}\mathcal {L}_\lambda (x)\\&-\int _{\tau \ge |x|>0}\frac{x^3}{1+x^2}\mathrm {d}\mathcal {L}_\lambda (x); \end{aligned}$$
4. $$\begin{aligned} \lim _{\tau \rightarrow 0}\limsup _{l\rightarrow \infty }\; M_l^\lambda Var\left( \frac{\tilde{\chi }_{n_l}^{1*}}{K_l^\lambda }\mathbf {1}_{\left\{ \tilde{\chi }_{n_l}^{1*}\le \tau K_l^\lambda \right\} }\right) =0. \end{aligned}$$

We prove each of these in turn but we start by introducing a relation which will be fundamental to proving the final parts. For $K \in \mathbb {Z}$ let $c_l^{\scriptscriptstyle {K}}=\mathbf {P}(\mathcal {H}(\mathcal {T}^{*-})> l+K| \mathcal {H}(\mathcal {T}^{*-})> h_{n_l,\varepsilon })$ denote the probability that a deep branch is of height at least $l+K$. Then by the asymptotic (3.12) we have that, for *K* such that $l+K\ge h_{n_l,\varepsilon }$, as $l \rightarrow \infty$

$$\begin{aligned} c_l^{\scriptscriptstyle {K}} \; = \; \frac{\mathbf {P}\left( \mathcal {H}(\mathcal {T}^{*-})>l+K\right) }{\mathbf {P}\left( \mathcal {H}(\mathcal {T}^{*-})>h_{n_l,\varepsilon }\right) } \; \sim \; \mu ^{(\alpha -1)K}\frac{\mathbf {P}\left( \mathcal {H}(\mathcal {T}^{*-})>l\right) }{\mathbf {P}\left( \mathcal {H}(\mathcal {T}^{*-})>h_{n_l,\varepsilon }\right) }. \end{aligned}$$

In particular, using (3.12) and that $\beta ^{\gamma _\alpha }=\mu ^{-(\alpha -1)}$

$$\begin{aligned}&M_l^\lambda c_l^{\scriptscriptstyle {K}} \sim \lambda ^{\gamma _\alpha } \left( \frac{\mathbf {P}\left( \xi ^*>\mu ^{h_{n_l,\varepsilon }}\right) }{\mathbf {P}\left( \mathcal {H}\left( \mathcal {T}^{*-}\right)>h_{n_l,\varepsilon }\right) }\right) \left( \frac{\mathbf {P}\left( \mathcal {H}\left( \mathcal {T}^{*-}\right)>h_{n_l,0}\right) }{\mathbf {P}\left( \xi ^*>\mu ^{h_{n_l,0}}\right) }\right) \\&\quad \frac{\beta ^{-\gamma _\alpha l}}{\mu ^{-(\alpha -1)l}}\mu ^{(\alpha -1)K} \sim \lambda ^{\gamma _\alpha }\beta ^{-\gamma _\alpha K} \end{aligned}$$

thus $M_l^\lambda (c_l^{\scriptscriptstyle {K}}-c_l^{\scriptscriptstyle {K}+1})\rightarrow \lambda ^{\gamma _\alpha }\beta ^{-\gamma _\alpha K}(1-\beta ^{-\gamma _\alpha })$ and for any $\epsilon >0$ and large enough *l*

8.1 $$\begin{aligned} M_l^\lambda c_l^{\scriptscriptstyle {K}} \le C_\epsilon \lambda ^{\gamma _\alpha } \beta ^{-\gamma _\alpha K}\beta ^{\epsilon \left| K\right| }. \end{aligned}$$

To prove (1), notice that

$$\begin{aligned} \mathbb {P}\left( \frac{\tilde{\chi }_{n_l}^{1*}}{K_l^\lambda }>\epsilon \right) \le \mathbf {P}\left( \mathcal {H}(\mathcal {T}^{*-})\ge h_{n,\varepsilon /2}\big |\mathcal {H}(\mathcal {T}^{*-})\ge h_{n,\varepsilon }\right) + \mathbb {P}\left( \beta ^{h_{n,\varepsilon /2}-l}Z_{sup}>\lambda \epsilon \right) . \end{aligned}$$

Both terms converge to 0 as $l \rightarrow \infty$ by the tail formula of a branch (3.12), the fact that $Z_{sup}$ has no atom at $\infty$ and that $\beta ^{h_{n_l}^{\varepsilon /2}-l}\rightarrow 0$ which follows from $l\sim h_{n,0}$.

For (2), recall that $F_{\scriptscriptstyle {K}}^l(x)=\mathbb {P}(\zeta _i^{l,K}>x)=\mathbb {P}^{\scriptscriptstyle {K}}\left( \tilde{\chi }_{n_l}^{1*}\beta ^{-(l+K)}>x\right)$, therefore

$$\begin{aligned} M_l^\lambda \mathbb {P}\left( \frac{\tilde{\chi }_{n_l}^{1*}}{K_l^\lambda }>x\right)&= \sum _{K \in \mathbb {Z}}\mathbf {1}_{\left\{ K\ge -(l-h_{n_l,\varepsilon })\right\} }M_l^\lambda \mathbf {P}\left( \mathcal {H}(\mathcal {T}^{*-})=l+K\right) \mathbb {P}^{\scriptscriptstyle {K}} \left( \frac{\tilde{\chi }_{n_l}^{1*}}{K_l^\lambda }>x\right) \\&= \sum _{K \in \mathbb {Z}}\mathbf {1}_{\left\{ K\ge -(l-h_{n_l,\varepsilon })\right\} }M_l^\lambda \left( c_l^{\scriptscriptstyle {K}}-c_l^{\scriptscriptstyle {K}+1}\right) \overline{F}_{\scriptscriptstyle {K}}^l(\lambda \beta ^{-K}x). \end{aligned}$$

If $x>0$ is a continuity point of $\mathcal {L}_\lambda$ then $\lambda x\beta ^{-K}$ is a continuity point of $\overline{F}_\infty$ hence for any $K\in \mathbb {Z}$ as $l \rightarrow \infty$

$$\begin{aligned} \mathbf {1}_{\left\{ K \ge -(l-h_{n_l,\varepsilon })\right\} }M_l^\lambda \left( c_l^{\scriptscriptstyle {K}}-c_l^{\scriptscriptstyle {K}+1}\right) \overline{F}_{\scriptscriptstyle {K}}^l(\lambda \beta ^{-K}x) \rightarrow \lambda ^{\gamma _\alpha }\beta ^{-\gamma _\alpha K}(1-\beta ^{-\gamma _\alpha })\overline{F}_\infty (\lambda \beta ^{-K}x). \end{aligned}$$

We need to exchange the sum and the limit; we do this using dominated convergence. Since $\gamma _\alpha <1$ we can choose $\epsilon >0$ such that $\gamma _\alpha +\epsilon <1$ and $\epsilon <\gamma _\alpha$. By (8.1), for *l* sufficiently large $M_l^\lambda c_l^{\scriptscriptstyle {K}} \le C_{\epsilon ,\lambda }\beta ^{-\gamma _\alpha K}\beta ^{\frac{\epsilon }{2}|K|}$ hence

$$\begin{aligned} \sum _{K\ge -(l-h_{n_l,\varepsilon })}M_l^\lambda \left( c_l^{\scriptscriptstyle {K}}-c_l^{\scriptscriptstyle {K}+1}\right) \overline{F}_{\scriptscriptstyle {K}}^l\left( \lambda \beta ^{-K}x\right) \le C\sum _{K\in \mathbb {Z}}\overline{F}_{sup}\left( \lambda x\beta ^{-K}\right) \beta ^{-\gamma _\alpha K}\beta ^{\frac{\epsilon }{2}|K|}. \end{aligned}$$

Since $Z_{sup}$ has moments up to $\gamma _\alpha +\epsilon$ we have that for $y=\lambda x$

$$\begin{aligned} \sum _{K< 0}\overline{F}_{sup}(\lambda x\beta ^{-K})\beta ^{-\gamma _\alpha K}\beta ^{\frac{\epsilon }{2}\left| K\right| } \; = \; \mathbb {E}\left[ \sum _{K=0}^{\left\lfloor \frac{\log (Z_{sup}/y)}{\log (\beta )}\right\rfloor } \beta ^{K\left( \gamma _\alpha + \epsilon /2\right) }\right] \; \le \; C_y\mathbb {E}\left[ Z_{sup}^{\gamma _\alpha +\frac{\epsilon }{2}}\right] \end{aligned}$$

which is finite. By choice of $\epsilon$ it follows that

$$\begin{aligned} \sum _{K\ge 0}\overline{F}_{sup}(\lambda x\beta ^{-K})\beta ^{-\gamma _\alpha K}\beta ^{\frac{\epsilon }{2}\left| K\right| } \le \sum _{K\ge 0}\beta ^{\left( \frac{\epsilon }{2}-\gamma _\alpha \right) K} <\infty . \end{aligned}$$

It therefore follows that for $x>0$

$$\begin{aligned} -\lim _{l\rightarrow \infty } M_l^\lambda \mathbb {P}\left( \frac{\tilde{\chi }_{n_l}^{1*}}{K_l^\lambda }>x \right) =-\lambda ^{\gamma _\alpha } \left( 1-\beta ^{-\gamma _\alpha }\right) \sum _{K\in \mathbb {Z}}\overline{F}_\infty \left( \lambda x \beta ^{\gamma _\alpha K}\right) \beta ^{\gamma _\alpha K}. \end{aligned}$$

Moreover, for $x<0$ we have that $\mathbb {P}\left( \tilde{\chi }_{n_l}^{1*}/K_l^\lambda <x \right) =0$ which gives (2).

For (3) we have that $\int _0^\tau x \mathrm {d}\mathcal {L}_\lambda$ is well defined therefore

$$\begin{aligned} \int _\tau ^\infty \frac{x}{1+x^2} \mathrm {d}\mathcal {L}_\lambda - \int _0^\tau \frac{x^3}{1+x^2} \mathrm {d}\mathcal {L}_\lambda \; = \; \int _0^\infty \frac{x}{1+x^2} \mathrm {d}\mathcal {L}_\lambda -\int _0^\tau x \mathrm {d}\mathcal {L}_\lambda . \end{aligned}$$

We therefore want to show that

$$\begin{aligned} \lim _{l \rightarrow \infty }\frac{M_l^\lambda }{K_l^\lambda } \mathbb {E}\left[ \tilde{\chi }_{n_l}^{1*}\mathbf {1}_{\left\{ \tilde{\chi }_{n_l}^{1*}\le \tau K_l^\lambda \right\} }\right] = \int _0^\tau x \mathrm {d}\mathcal {L}_\lambda . \end{aligned}$$

Write $G_{\scriptscriptstyle {K}}^l(u)=\mathbb {E}\left[ \zeta ^{l,K}_1\mathbf {1}_{\{\zeta ^{l,K}_1\le u\}} \right]$ and $G_\infty (u)=\mathbb {E}[Z_\infty \mathbf {1}_{\{Z_\infty \le u\}}]$. Then we have that

$$\begin{aligned} \frac{M_l^\lambda }{K_l^\lambda } \mathbb {E}\left[ \tilde{\chi }_{n_l}^{1*}\mathbf {1}_{\left\{ \tilde{\chi }_{n_l}^{1*}\le \tau K_l^\lambda \right\} }\right]&= \lambda ^{-1}\sum _{K\ge -(l-h_{n_l,\varepsilon })}M_l^\lambda \left( c_l^{\scriptscriptstyle {K}}-c_l^{\scriptscriptstyle {K}+1}\right) \beta ^{K} G_{\scriptscriptstyle {K}}^l(\tau \lambda \beta ^{-K}). \end{aligned}$$

For each $K\in \mathbb {Z}$ as $l\rightarrow \infty$

$$\begin{aligned} M_l^\lambda \left( c_l^{\scriptscriptstyle {K}} -c_l^{\scriptscriptstyle {K}+1}\right) \beta ^{K} G_{\scriptscriptstyle {K}}^l(\tau \lambda \beta ^{-K}) \rightarrow \lambda ^{\gamma _\alpha }(1-\beta ^{-\gamma _\alpha })\beta ^{(1-\gamma _\alpha )K}G_\infty (\tau \lambda \beta ^{-K}). \end{aligned}$$

We want to exchange the limit and the sum which we do by dominated convergence. For any $\kappa \in [0,1]$ and random variable *Y* we have that $\mathbf {E}[Y\mathbf {1}_{\{Y \le u\}}]\le u^\kappa \mathbf {E}[Y^{1-\kappa }\mathbf {1}_{\{Y\le u\}}]$. Using this with $u=\tau \lambda \beta ^{-K}$ where $\kappa =1-\gamma _\alpha -2\epsilon /3$ for $K< 0$ and $\kappa =1$ for $K\ge 0$, alongside (8.1) we have that

$$\begin{aligned}&\sum _{K\in \mathbb {Z}}\mathbf {1}_{\left\{ K \ge -(l-h_{n_l,\varepsilon })\right\} }M_l^\lambda \left( c_l^{\scriptscriptstyle {K}}-c_l^{\scriptscriptstyle {K}+1}\right) \beta ^K G_{\scriptscriptstyle {K}}^l(\tau \lambda \beta ^{-K}) \\&\qquad \qquad \le \sum _{K\ge 0}M_l^\lambda \left( c_l^{\scriptscriptstyle {K}} -c_l^{\scriptscriptstyle {K}+1}\right) \beta ^K\tau \lambda \beta ^{-K} \\&\qquad \quad \qquad + \sum _{K<0} M_l^\lambda \left( c_l^{\scriptscriptstyle {K}} -c_l^{\scriptscriptstyle {K}+1}\right) \beta ^K \left( \beta ^{\frac{2\epsilon }{3} K}(\tau \lambda )^{1-\gamma _\alpha -\frac{2\epsilon }{3}}\mathbf {E}\left[ Z_{sup}^{\gamma _\alpha +\frac{2\epsilon }{3}}\right] \beta ^{(\gamma _\alpha -1)K}\right) \\&\qquad \qquad \le C_\lambda \tau \sum _{K\ge 0} \beta ^{-\left( \gamma _\alpha -\epsilon /2\right) K} + C_\lambda \tau ^{1-\gamma _\alpha -\frac{2\epsilon }{3}}\mathbf {E}\left[ Z_{sup}^{\gamma _\alpha +\frac{2\epsilon }{3}}\right] \sum _{K<0}\beta ^{\frac{\epsilon }{6} K} \end{aligned}$$

which is finite since $\gamma _\alpha >\epsilon /2$ and $Z_{sup}$ has moments up to $\gamma _\alpha +\epsilon$. We therefore have that

$$\begin{aligned} \lim _{l\rightarrow \infty } \frac{M_l^\lambda }{K_l^\lambda } \mathbf {E}\left[ \tilde{\chi }_{n_l}^{1*}\mathbf {1}_{\left\{ \tilde{\chi }_{n_l}^{1*}\le \tau K_l^\lambda \right\} }\right] = \lambda ^{\gamma _\alpha -1}(1-\beta ^{-\gamma _\alpha })\sum _{K\in \mathbb {Z}}\beta ^{K(\gamma _\alpha -1)}G_\infty (\tau \lambda \beta ^K). \end{aligned}$$

By definition we have that

$$\begin{aligned} \int _0^\tau x \mathrm {d}\mathcal {L}_\lambda&= \lambda ^{\gamma _\alpha } (1-\beta ^{-\gamma _\alpha })\int _0^\tau x\sum _{K\in \mathbb {Z}}\beta ^{\gamma _\alpha K}\mathrm {d}(-\overline{F}_\infty )(\lambda x \beta ^K) \\&= \lambda ^{\gamma _\alpha -1} (1-\beta ^{-\gamma _\alpha })\sum _{K\in \mathbb {Z}}\beta ^{(\gamma _\alpha -1) K}\int _{\lambda x\beta ^K\le \lambda \tau \beta ^K} \lambda x\beta ^K\mathrm {d}(-\overline{F}_\infty )(\lambda x \beta ^K) \\&= \lambda ^{\gamma _\alpha -1}(1-\beta ^{-\gamma _\alpha })\sum _{K\in \mathbb {Z}}\beta ^{(\gamma _\alpha -1)K}G_\infty (\tau \lambda \beta ^K). \end{aligned}$$

It therefore remains to calculate $\int _0^\infty \frac{x}{1+x^2}\mathrm {d}\mathcal {L}_\lambda$.

$$\begin{aligned} \int _0^\infty \frac{x}{1+x^2}\mathrm {d}\mathcal {L}_\lambda&= \lambda ^{\gamma _\alpha }(1-\beta ^{-\gamma _\alpha })\int _0^\infty \frac{x}{1+x^2} \sum _{K \in \mathbb {Z}}\beta ^{\gamma _\alpha K}\mathrm {d}(-\overline{F}_\infty )(\lambda x\beta ^K) \\&= \lambda ^{\gamma _\alpha +1}(1-\beta ^{-\gamma _\alpha })\sum _{K \in \mathbb {Z}}\beta ^{(\gamma _\alpha +1)K} \mathbb {E}\left[ \frac{Z_\infty }{(\lambda \beta ^K)^2+(Z_\infty )^2}\right] . \end{aligned}$$

The final sum is finite since for $K<0$

$$\begin{aligned} \beta ^{(\gamma _\alpha +1)K} \mathbb {E}\left[ \frac{Z_\infty }{(\lambda \beta ^K)^2+(Z_\infty )^2}\right] = \lambda ^{-1}\beta ^{\gamma _\alpha K} \mathbb {E}\left[ \frac{\lambda \beta ^KZ_\infty }{(\lambda \beta ^K)^2+(Z_\infty )^2}\right] \le \lambda ^{-1}\beta ^{\gamma _\alpha K} \end{aligned}$$

Which is summable and for $K\ge 0$

$$\begin{aligned} \mathbb {E}\left[ \frac{Z_\infty }{(\lambda \beta ^K)^2+(Z_\infty )^2}\right]&\le \mathbb {E}\left[ \frac{Z_\infty }{(\lambda \beta ^K)^2}\mathbf {1}_{\{Z_\infty \le \lambda \beta ^K\}}+ Z_\infty ^{-1}\mathbf {1}_{\{Z_\infty \ge \lambda \beta ^K\}}\right] \\&\le C_\lambda \mathbb {E}\left[ Z_{sup}^{\gamma _\alpha +\epsilon /2}\right] \beta ^{-K\left( 1+\gamma _\alpha +\epsilon /2\right) } \end{aligned}$$

which, multiplied by $\beta ^{(\gamma _\alpha +1)K}$, is summable.

It now remains to prove (4). It suffices to show that

8.2 $$\begin{aligned} \lim _{\tau \rightarrow 0^+}\lim _{l\rightarrow \infty } \frac{M_l^\lambda }{(K_l^\lambda )^2}\mathbb {E}\left[ \left( \tilde{\chi }_{n_l}^{1*}\right) ^2 \mathbf {1}_{\left\{ \tilde{\chi }_{n_l}^{1*}\le \tau K_l^\lambda \right\} }\right] =0. \end{aligned}$$

Write $$H_{\scriptscriptstyle {K}}^l(u)=\mathbb {E}\left[ (\zeta _1^{l,K})^2\mathbf {1}_{\{\zeta _1^{l,K}\le u\}} \right] $$ then

$$\begin{aligned} \frac{M_l^\lambda }{(K_l^\lambda )^2}\mathbb {E}\left[ (\tilde{\chi }_{n_l}^{1*})^2\mathbf {1}_{\left\{ \tilde{\chi }_{n_l}^{1*}\le \tau K_l^\lambda \right\} }\right]&= \frac{M_l^\lambda }{(K_l^\lambda )^2}\sum _{K\in \mathbb {Z}}(c_l^{\scriptscriptstyle {K}} -c_l^{\scriptscriptstyle {K}+1}) \beta ^{2(l+K)}H_{\scriptscriptstyle {K}}^l(\tau \lambda \beta ^{-K})\\&\le C_\lambda \sum _{K\in \mathbb {Z}}\beta ^{(2-\gamma _\alpha )K}\beta ^{\frac{\epsilon }{2}\left| K\right| } H_{\scriptscriptstyle {K}}^{l}(\tau \lambda \beta ^{-K}). \end{aligned}$$

Using that for any random variable *Y* we have $\mathbb {E}[Y^2\mathbf {1}_{\{Y\le u\}}]\le u^\kappa \mathbb {E}[Y^{2-\kappa }\mathbf {1}_{\{Y\le u\}}]$ with $u=\tau \lambda \beta ^{-K}$ and $\kappa =2$ it follows that

$$\begin{aligned} \sum _{K \ge 0}\beta ^{(2-\gamma _\alpha )K}\beta ^{\frac{\epsilon }{2}\left| K\right| } H_{\scriptscriptstyle {K}}^l(\tau \lambda \beta ^{-K}) \; \le \; C\tau ^2\sum _{K\ge 0}\beta ^{-\left( \gamma _\alpha -\epsilon /2\right) K} \; \le \; C\tau ^2 \end{aligned}$$

where the constant *C* depends on $\lambda , \beta , \gamma _\alpha$ and $\epsilon$. Then, with $u=\tau \lambda \beta ^{-K}, \; \kappa =2-\gamma _\alpha -2\epsilon /3$ we have that

$$\begin{aligned} H_{\scriptscriptstyle {K}}^l\left( \tau \lambda \beta ^{-K}\right) \beta ^{(2-\gamma _\alpha )K}\le \beta ^{\frac{2\epsilon }{3}K}(\tau \lambda )^{2-\gamma _\alpha -\frac{2\epsilon }{3}}\mathbf {E}\left[ Z_{sup}^{\gamma _\alpha +\frac{2\epsilon }{3}}\right] \end{aligned}$$

and therefore

$$\begin{aligned} \sum _{K \le 0}\beta ^{\left( 2-\gamma _\alpha \right) K}\beta ^{\frac{\epsilon }{2}|K|} H_{\scriptscriptstyle {K}}^l\left( \tau \lambda \beta ^{-K}\right)&\le \; C\tau ^{2-\gamma _\alpha -\frac{2\epsilon }{3}}\mathbf {E}\left[ Z_{sup}^{\gamma _\alpha +\frac{2\epsilon }{3}}\right] \sum _{K\le 0}\beta ^{\frac{\epsilon }{6}K}\\&\le \; C\tau ^{2-\gamma _\alpha -\frac{2\epsilon }{3}}. \end{aligned}$$

Since $\gamma _\alpha +\frac{2\epsilon }{3}<1$ we have that (8.2) holds. $\square$

Combining Proposition 8.1 with Corollary 5.5 and Lemma 5.6 with

$$\begin{aligned} \lambda =\Gamma (2-\alpha )^{\frac{1}{\gamma _\alpha }} c_\mu ^{\frac{1}{\gamma }}\beta ^{\frac{\log (t)}{\log (\mu ^{-1})}-\left\lfloor \frac{\log (t)}{\log (\mu ^{-1})}\right\rfloor } \end{aligned}$$

proves Theorem 3.

## Tightness

We conclude the results for the walk on the subcritical tree with Theorem 4 which is a tightness result for the process and a convergence result for the scaling exponent. We only prove the result in IVIE since the proof is standard (similar to that of Theorem 1.1 of [4]) and the other cases follow by the same method; however, we state the proof more generally. Recall that $r_n$ is $a_n$ in IVFE, $n^{1/\gamma }$ in FVIE, $a_n^{1/\gamma }$ in IVIE and $\overline{r}_n:=\max \{m\ge 0:r_m\le n\}$.

### Proof of Theorem 4 in IVIE

For statement 1 we show that $\lim _{t\rightarrow \infty }\limsup _{n\rightarrow \infty }\mathbb {P}\left( \Delta _n/r_n \notin [t^{-1},t]\right) =0$. Let *l* be such that $a_{n_l(1)} \le a_n < a_{n_{l+1}(1)}$ then by monotonicity of $\Delta _n$

$$\begin{aligned} \mathbb {P}\left( \frac{\Delta _n}{a_n^{1/\gamma }} \notin \left[ t^{-1},t\right] \right)&\le \mathbb {P}\left( \frac{\Delta _{n_l(1)}}{a_{n_{l+1}(1)}^{1/\gamma }}<t^{-1}\right) + \mathbb {P}\left( \frac{\Delta _{n_{l+1}(1)}}{a_{n_l(1)}^{1/\gamma }}>t\right) . \end{aligned}$$

The distribution of $R_1$ is continuous by Theorem III.2 of [23] since $\lim _{x\rightarrow 0}\mathcal {L}(x)=-\infty$ (where $R_t$ denotes the limiting distribution); therefore, since the sequence $(a_{n_{l+1}(1)}/a_{n_l(1)})^{1/\gamma }$ can be bounded above by some constant *c*,

$$\begin{aligned} \lim _{t\rightarrow \infty }\limsup _{n\rightarrow \infty }\mathbb {P}\left( \Delta _n/r_n \notin \left[ t^{-1},t\right] \right) \le \lim _{t\rightarrow \infty } \mathbb {P}\left( R_1 \notin \left[ (tc)^{-1},tc\right] \right) =0. \end{aligned}$$

For statement 2 we want to show that $\lim _{t\rightarrow \infty }\limsup _{n\rightarrow \infty }\mathbb {P}\left( |X_n|/\overline{r}_n \notin [t^{-1},t]\right) =0$. To do this we compare $|X_n|$ with $\Delta _n$. In order to deal with the depth $X_n$ reaches into the traps we use a bound for the height of a trap; for any $\epsilon >0$ we have

$$\begin{aligned} \mathbb {P}\left( \frac{\left| X_n\right| }{\overline{r}_n}\ge t\right) \le \mathbb {P}\left( \Delta _{\left\lfloor t\overline{r}_n-\overline{r}_n^\epsilon \right\rfloor }\le n\right) +(t\overline{r}_n-\overline{r}_n^\epsilon )\mathbb {P}\left( \mathcal {H}(\mathcal {T}^{*-})\ge \overline{r}_n^\epsilon \right) . \end{aligned}$$

By (3.12) we have that $(t\overline{r}_n-\overline{r}_n^\epsilon )\mathbb {P}\left( \mathcal {H}(\mathcal {T}^{*-})\ge \overline{r}_n^\epsilon \right) \rightarrow 0$ as $n \rightarrow \infty$. Using the definition of $\overline{r}_n$ we have that

$$\begin{aligned} \mathbb {P}\left( \Delta _{\left\lfloor t\overline{r}_n-\overline{r}_n^\epsilon \right\rfloor }\le n\right) \le \mathbb {P}\left( \frac{\Delta _{\left\lfloor t\overline{r}_n-\overline{r}_n^\epsilon \right\rfloor }}{a_{t\overline{r}_n-\overline{r}_n^\epsilon }^{1/\gamma }}\le \frac{a_{\overline{r}_n+1}^{1/\gamma }}{a_{t\overline{r}_n-\overline{r}_n^\epsilon }^{1/\gamma }}\right) . \end{aligned}$$

Since $a_{\overline{r}_n+1}^{1/\gamma }/a_{t\overline{r}_n-\overline{r}_n^\epsilon }^{1/\gamma }$ converges to $t^{-1/\gamma _\alpha }$ as $n \rightarrow \infty$, by continuity of the distribution of $R_1$ and statement 1 we have that $\lim _{t\rightarrow \infty }\limsup _{n\rightarrow \infty }\mathbb {P}\left( |X_n|/\overline{r}_n >t\right) =0$.

It remains to show that $\lim _{t\rightarrow \infty }\limsup _{n\rightarrow \infty }\mathbb {P}\left( |X_n|/\overline{r}_n <t^{-1}\right) =0$. We need to bound how far the walker backtracks after reaching a new furthest point in order to compare $|X_n|$ with $\Delta _n$. Let $\upsilon _0:=0$ and for $j\ge 1$ define the $j{\text {th}}$ regeneration time as $\upsilon _j:=\min \{m>\upsilon _{j-1}: \; \{X_n\}_{n=0}^{m-1}\cap \{X_n\}_{n=m}^\infty =\phi \}$ then

$$\begin{aligned} \max _{i<j\le n} \left( \left| X_i\right| -\left| X_j\right| \right) \le \upsilon _1 \vee \max _{2\le i\le n} \left( \upsilon _i-\upsilon _{i-1}\right) + \max _{0\le i \le n}\mathcal {H}\left( \mathcal {T}^{*-}_{\rho _i}\right) . \end{aligned}$$

The regeneration times $(\upsilon _i-\upsilon _{i-1}), \upsilon _1$ and the heights of branches $\mathcal {H}(\mathcal {T}^{*-}_{\rho _i})$ have exponential moments for all *i* therefore for any $\epsilon >0$ by a union bound

$$\begin{aligned} \lim _{n \rightarrow \infty }\mathbb {P}\left( \max _{i<j\le n} \left( \left| X_i\right| -\left| X_j\right| \right) >\overline{r}_n^\epsilon \right) =0. \end{aligned}$$

We then have that

$$\begin{aligned} \mathbb {P}\left( \left| X_n\right| /\overline{r}_n<t^{-1}\right)&\le \mathbb {P}\left( \max _{i<j\le n} \left| X_i\right| -\left| X_j\right|>\overline{r}_n^\epsilon \right) + \mathbb {P}\left( \Delta _{\left\lfloor t^{-1}\overline{r}_n+\overline{r}_n^\epsilon \right\rfloor }>n\right) \\&\le o(1) + \mathbb {P}\left( \frac{\Delta _{\left\lfloor 2t^{-1}\overline{r}_n\right\rfloor }}{a_{2t^{-1}\overline{r}_n}^{1/\gamma }} >\frac{a_{\overline{r}_n}^{1/\gamma }}{a_{2t^{-1} \overline{r}_n}^{1/\gamma }}\right) . \end{aligned}$$

Then, since $a_{\overline{r}_n}^{1/\gamma }/a_{2t^{-1}\overline{r}_n}^{1/\gamma } \rightarrow (t/2)^{1/\gamma _\alpha }$ as $n \rightarrow \infty$, by continuity of the distribution of $R_1$ and statement 1 we indeed have that $\lim _{t\rightarrow \infty }\limsup _{n\rightarrow \infty }\mathbb {P}\left( |X_n|/\overline{r}_n <t^{-1}\right) =0$.

For the final statement notice that

$$\begin{aligned} \mathbb {P}\left( \lim _{n \rightarrow \infty }\frac{\log |X_n|}{\log (n)}\ne \gamma (\alpha -1)\right) =\mathbb {P}\left( \lim _{n \rightarrow \infty }\frac{\log \left| X_n\right| }{\log \left( \overline{r}_n\right) } \cdot \frac{\log \left( \overline{r}_n\right) }{\log (n)}\ne \gamma (\alpha -1)\right) \end{aligned}$$

and since $\overline{r}_n=n^{\gamma (\alpha -1)}\tilde{L}(n)$ for some slowly varying function $\tilde{L}$ we have that as $n \rightarrow \infty$ $\log (\overline{r}_n)/\log (n) \rightarrow \gamma (\alpha -1)$ thus it suffices to show that the following is equal to 0

$$\begin{aligned} \mathbb {P}\left( \lim _{n \rightarrow \infty }\frac{\log |X_n|}{\log \left( \overline{r}_n\right) }\ne 1\right) \le \mathbb {P}\left( \limsup _{n \rightarrow \infty } \frac{\log \left| X_n\right| }{\log \left( \overline{r}_n\right) }>1\right) + \lim _{t\rightarrow \infty }\mathbb {P}\left( \liminf _{n \rightarrow \infty } \frac{|X_n|}{\overline{r}_n} \le t^{-1}\right) . \end{aligned}$$

By Fatou we can bound the second term above by $\lim \nolimits _{t \rightarrow \infty } \liminf \nolimits _{n \rightarrow \infty } \; \mathbb {P}\left( |X_n|/\overline{r}_n \le t^{-1}\right)$ which is equal to 0 by tightness of $(|X_n|/\overline{r}_n)_{n\ge 0}$.

For the first term we have

$$\begin{aligned} \mathbb {P}\left( \limsup _{n \rightarrow \infty } \frac{\log \left| X_n\right| }{\log \left( \overline{r}_n\right) }>1\right)&= \lim _{\varepsilon \rightarrow 0^+}\mathbb {P}\left( \limsup _{n \rightarrow \infty }\frac{\log \left| X_n\right| }{\log \left( \overline{r}_n\right) }\ge 1+\varepsilon \right) \\&\le \lim _{\varepsilon \rightarrow 0^+}\mathbb {P}\left( \lim _{n \rightarrow \infty }\frac{\sup _{k\le n}\left| X_n\right| }{\overline{r}_n^{1+\varepsilon }}\ge 1\right) . \end{aligned}$$

Writing $D'(n):=\left\{ \max \limits _{i=0,\ldots ,n}\mathcal {H}(\mathcal {T}^{*-}_{\rho _i})\le 4\log (a_n)/\log (\mu ^{-1})\right\}$ we have that $\mathbb {P}(D'(n)^c)=o(n^{-2})$ by (3.12) thus $\mathbf {P}(D'(n)^c \; i.o.)=0$. On $D'(n)$

$$\begin{aligned} \sup _{k\le n}\left| X_k\right| \le \left| X_{\kappa _n}\right| + \kappa _{n+1}-\kappa _n+\frac{4\log (a_n)}{\log \left( \mu ^{-1}\right) } \end{aligned}$$

where $\kappa _n$ is the last regeneration time of *Y* before time *n*. Therefore, since $\kappa _{n+1}-\kappa _n$ have exponential moments we have that $\mathbb {P}(\limsup _{n \rightarrow \infty }(\kappa _{n+1}-\kappa _n)\ge \overline{r}_n) =0$; hence,

$$\begin{aligned} \mathbb {P}\left( \lim _{n \rightarrow \infty }\frac{\sup _{k\le n}\left| X_n\right| }{\overline{r}_n^{1+\varepsilon }}\ge 1\right)\le & {} \mathbb {P}\left( \liminf _{n \rightarrow \infty }\frac{\left| X_{\kappa _n}\right| }{\overline{r}_n^{1+\varepsilon }}\ge 1-o(1)\right) \\\le & {} \lim _{t\rightarrow \infty }\liminf _{n \rightarrow \infty }\mathbb {P}\left( \frac{\left| X_n\right| }{\overline{r}_n}\ge t\right) \end{aligned}$$

where the second inequality follows by Fatou’s lemma. The result follows by tightness of $(|X_n|/\overline{r}_n)_{n\ge 0}$. $\square$

Theorem 1 follows from Theorem 4, Proposition 6.7 and Corollary 5.6 with Ê$\lambda =t$ since $nq_n\sim n^\varepsilon$. More specifically, since $R_{d_t,0,\mathcal {L}_t}$ is the infinitely divisible law with characteristic exponent

$$\begin{aligned} id_1t + \int _0^\infty e^{itx}-1-\frac{itx}{1+x^2}\mathrm {d}\mathcal {L}_1(x)&= \int _0^\infty e^{itx}-1 d\mathcal {L}_1(x) \\&= t^{-(\alpha -1)}\int _0^\infty e^{ix}-1 d\mathcal {L}_1(x) \end{aligned}$$

by a simple change of variables calculation we have that the laws of the process $\left( \Delta _{nt}/a_n\right) _{t\ge 0}$ converge weakly as $n \rightarrow \infty$ under $\mathbb {P}$ with respect to the Skorohod $J_1$ topology on $D([0,\infty ),\mathbb {R})$ to the law of the stable subordinator with characteristic function $\varphi (t)=e^{-C_\alpha t^{\alpha -1}}$ where $C_{\alpha ,\beta ,\mu }=-\int _0^\infty e^{ix}-1 d\mathcal {L}_1(x)$. A straightforward calculation then shows that the Laplace transform is of the form

$$\begin{aligned} \varphi _t(s) := \mathbb {E}\left[ e^{-sRF_{d_t,0,\mathcal {L}_t}}\right] = e^{-ts^{\alpha -1} C_{\alpha ,\beta ,\mu }} \end{aligned}$$

where

9.1 $$\begin{aligned} C_{\alpha ,\beta ,\mu }=\frac{\pi (\alpha -1)}{\sin \left( \pi (\alpha -1)\right) }\cdot \left( \frac{\beta (1-\beta \mu )}{2(\beta -1)}\right) ^{\alpha -1}. \end{aligned}$$

## Supercritical tree

**Fig. 5:**
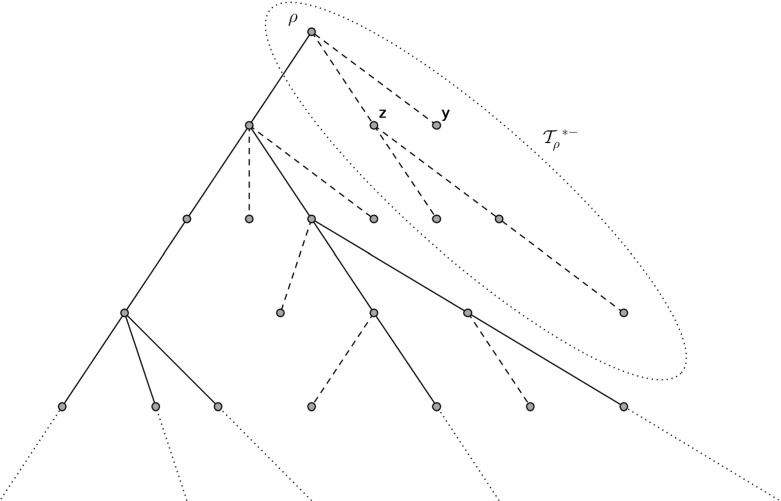
A sample section of a supercritical tree with *solid lines* representing the backbone and *dashed lines* representing the dangling ends

As discussed in the introduction, the structures of the supercritical and subcritical trees are very similar in that they consist of some backbone structure $\mathcal {Y}$ with subcritical GW-trees as leaves. The main differences are as follows:

- On the subcritical tree the backbone was a single infinite line of descent, represented by the solid line in Fig. 2 of Sect. 3. On the supercritical tree the backbone is itself a random tree, represented by the solid line in Fig. 5. In particular, it is a GW-tree without deaths whose law is determined by the generating function $g(s):=\left( f\left( (1-q)s+q\right) -q\right) /(1-q)$ where *f* is the generating function of the original offspring law and *q* is the extinction probability.
- Each backbone vertex has additional children which we call buds. On the subcritical tree, the number of buds had a size-biased law independent of the position on the backbone. On the supercritical tree, the distribution over the number of buds is more complicated since it depends on the backbone. Importantly, the expected number of buds can be bounded above by $\mu (1-q)^{-1}$ independently of higher moments of the offspring law which isn’t the case for the subcritical tree.
- In the subcritical case, the GW-trees forming the traps have the law of the original (unconditioned) offspring law. In the supercritical case, the law is defined by the p.g.f. $h(s):=f(qs)/q$ which has mean $f'(q)$.

Let $\mathcal {T}$ denote the supercritical tree conditioned to survive, $\mathcal {T}^\circ$ the unconditioned tree and $\mathcal {T}^-$ the tree conditioned to die out. Write $Z_n, Z_n^\circ , Z_n^-$ to be the size of their $n{\text {th}}$ generations respectively and $V_n,V_n^\circ$ to be the number of vertices in the $n{\text {th}}$ generation of the backbone (for $\mathcal {T}$ and $\mathcal {T}^\circ$). As in the subcritical case we denote $\mathcal {T}^{*-}$ to be a dummy branch formed by a backbone vertex, its buds and the associated traps. In Fig. 5, the dashed lines represent the finite structures comprised of the buds and leaves. It will be convenient to refer to the traps at a site so for $x \in \mathcal {Y}$ let $L_x$ denote the collection of traps adjacent to *x*, for example in Fig. 5 $L_\rho$ consists of the two trees rooted at *y*, *z*. We then write $\mathcal {T}^{*-}_{x}$ to be the branch at *x*.

Recall that Theorem 5 states that if offspring law belongs to the domain of attraction of some stable law of index $\alpha \in (1,2)$, has mean $\mu >1$ and the derivative of the generating function at the extinction probability satisfies $\beta >f'(q)^{-1}$. Then,

$$\begin{aligned} \frac{\Delta _{n_l(t)}}{n_l(t)^\frac{1}{\gamma }} \rightarrow R_t \end{aligned}$$

in distribution as $l\rightarrow \infty$ under $\mathbb {P}$, where $\gamma$ is as given in (1.1), $n_l(t)=\lfloor t f'(q)^{-l}\rfloor$ for $t>0$ and $R_t$ is a random variable with an infinitely divisible law whose parameters are given in [4]. Moreover, the laws of $(\Delta _nn^{-\frac{1}{\gamma }})_{n\ge 0}$ and $(|X_n|n^{-\gamma })_{n\ge 0}$ under $\mathbb {P}$ are tight on $(0,\infty )$ and $\mathbb {P}$-a.s.

$$\begin{aligned} \lim _{n \rightarrow \infty }\frac{\log |X_n|}{\log (n)}&=\gamma . \end{aligned}$$

In [4] it is shown that this holds when $\mu >1, \mathbf {E}[\xi ^2]<\infty$ and $\beta >f'(q)^{-1}$. In order to extend this result to prove Theorem 5 it will suffice to prove Lemmas 10.1, 10.2 and 10.3 which we defer to the end of the section.

In Lemma 10.1 we show that $\mathbf {P}(\mathcal {H}(\mathcal {T}^{*-})>n) \sim C^*f'(q)^n$ for some constant $C^*$. This is the same as when $\mathbf {E}[\xi ^2]<\infty$ for the supercritical tree unlike for the subcritical tree where the exponent changes depending on the stability. This is because the first moment of the bud distribution has a fundamental role and the change from finite to infinite variance changes this for the subcritical tree but not for the supercritical tree. Lemma 10.1 is an extension of Lemma 6.1 of [4] which is proved using a Taylor expansion of the *f* around 1 up to second moments. We cannot take this approach because $f''(1)=\infty$; instead we use the form of the generating function determined in Lemma 3.3. The expression is important because, as in FVIE, the expected time spent in a large branch of height $\mathcal {H}(\mathcal {T}^{*-})$ is approximately $c\beta ^{\mathcal {H}(\mathcal {T}^{*-})}$ for some constant *c*.

Lemma 10.2 shows that, with high probability, no large branch contains more than one large trap. This is important because the number of large traps would affect the escape probability. That is, if there are many large traps in a branch then it is likely that the root has many offspring on the backbone since some geometric number of the offspring lie on the backbone. The analogue of this in [4] is proved using the bound $f'(1)-f'(1-\epsilon )\le C\epsilon$ which follows because $f''(1)<\infty$. Similarly to Lemma 10.1, we use a more precise form of *f* in order to obtain a similar bound.

Lemma 10.3 shows that no branch visited by level *n* is too large. This is important for the tightness result since we need to bound the deviation of the walk from the furthest point reached along the backbone. The proof of this follows quite straightforwardly from Lemma 10.1.

To explain why these are needed, we recall the argument which follows a similar structure to the proof of Theorem 2. As was the case for the walk on the subcritical tree, the first part of the argument involves showing that, asymptotically, the time spent outside large branches is negligible. This follows by the same techniques as for the subcritical tree.

One of the major difficulties with the walk on the supercritical tree is determining the distribution over the number of entrances into a large branch. The height of the branch from a backbone vertex *x* will be correlated with the number of children *x* has on the backbone. This affects the escape probability and therefore the number of excursions into the branch. It can be shown that the number of excursions into the first large trap converges in distribution to some non-trivial random variable $W_\infty$. In particular, it is shown in [4] that $W_\infty$ can be stochastically dominated by a geometric random variable and that there is some constant $c_W>0$ such that $\mathbb {P}(W_\infty >0)\ge c_W$.

Similarly to Sect. 4, it can be shown that asymptotically the large branches are independent in the sense that with high probability the walk won’t reach one large branch and then return to a previously visited large branch. Using Lemmas 10.1 and 10.2 (among other results) it can then be shown that $\Delta _n$ can be approximated by the sum of i.i.d. random variables.

The remainder of the proof of the first part of Theorem 5 involves decomposing the time spent in large branches, showing that the suitably scaled excursion times converge in distribution, proving the convergence results for sums of i.i.d. variables and concluding with standard tightness results similar to Sect. 9. Since $\mathbf {P}(Z_1^-=k)=p_kq^{k-1}$, the subcritical GW law over the traps has exponential moments. This means that these final parts of the proof follow by the results proven in [4] since, by Lemma 10.1, the scaling is the same as when $\mathbf {E}[\xi ^2]<\infty$.

Tightness of $(\Delta _nn^{-1/\gamma })_{n\ge 0}$ and $(X_nn^{-\gamma })_{n\ge 0}$ and almost sure convergence of $\log (|X_n|)/\log (n)$ then follow by the proof of Theorem 1.1 of [4] (with one slight adjustment) which is similar to the proof of Theorem 4. In order to bound the maximum distance between the walker’s current position and the last regeneration point we use a bound on the maximum height of a trap seen up to $\Delta _n^Y$. In [4] it is shown that the probability a trap of height at least $4\log (n)/\log (f'(q)^{-1})$ is seen is at most order $n^{-2}$ by using finite variance of the offspring distribution to bound the variance of the number of traps in a branch. In Lemma 10.3 we prove this using Lemma 10.1.

### Lemma 10.1

Under the assumptions of Theorem 5

$$\begin{aligned} \mathbf {P}\left( \mathcal {H}(\mathcal {T}^{*-})> n\right) \sim C^* f'(q)^n \end{aligned}$$

where $C^*=q(\mu -f'(q))c_\mu /(1-q)$ and $c_\mu$ is such that $\mathbf {P}(\mathcal {H}(\mathcal {T}^-)\ge n) \sim c_\mu f'(q)^n$.

### Proof

Let $Z:=Z_1, Z^\circ :=Z_1^\circ , V:=V_1, V^\circ :=V_1^\circ$ and $s_n:=\mathbf {P}(\mathcal {H}(\mathcal {T}^-)< n)$, then

$$\begin{aligned} \mathbf {P}\left( \mathcal {H}(\mathcal {T}^{*-})>n\right) = 1-\mathbf {E}\left[ \mathbf {P}\left( \mathcal {H}(\mathcal {T}^{*-})\le n|Z,V\right) \right] = 1-\frac{\mathbf {E}\left[ s_n^{Z^\circ -V^\circ }\mathbf {1}_{\left\{ V^\circ >0\right\} }\right] }{1-q}. \end{aligned}$$

For any $$t,s>0$$

$$\begin{aligned} \mathbf {E}\left[ s^{Z^\circ -V^\circ }t^{V^\circ }\right]&= \mathbf {E}\left[ s^{Z^\circ }\mathbf {E}[(t/s)^{V^\circ }\big |Z\right] \\&=\mathbf {E}\left[ s^{Z^\circ }\sum _{k=0}^{Z^\circ }(t/s)^k\left( {\begin{array}{c}Z^\circ \\ k\end{array}}\right) q^{Z^\circ -k}(1-q)^k\right] \\&=\mathbf {E}\left[ (qs)^{Z^\circ }\left( 1+\frac{t(1-q)}{qs}\right) ^{Z^\circ }\right] \\&= f(sq+t(1-q)). \end{aligned}$$

Furthermore,

$$\begin{aligned} \mathbf {E}\left[ s^{Z^\circ }\mathbf {1}_{\left\{ V^\circ =0\right\} }\right] =\mathbf {E}\left[ s^{Z^\circ }\mathbf {P}(V^\circ =0|Z^\circ )\right] =\mathbf {E}\left[ (sq)^{Z^\circ }\right] . \end{aligned}$$

Therefore, writing $t_n:= s_nq+1-q$ we have that $1-t_n=q(1-s_n)$ and

$$\begin{aligned} \mathbf {P}\left( \mathcal {H}(\mathcal {T}^{*-})> n\right) =1-\frac{f(s_nq+1-q)}{1-q}+\frac{f(s_nq)}{1-q}=\frac{(1-f(t_n))-(q-f(s_nq))}{1-q}. \end{aligned}$$

By Taylor we have that $\exists z \in [s_nq,q]$ such that $f(s_nq)=q+qf'(q)(s_n-1)+f''(z)q^2(s_n-1)^2/2$. Since $q<1$ we have that $f''(z)$ exists for all $z\le q$ and is bounded above by $f''(q)<\infty$.

By Lemma 3.3

$$\begin{aligned} 1-f(t_n)= \mu (1-t_n)+\frac{\Gamma (3-\alpha )}{\alpha (\alpha -1)}(1-t_n)^\alpha \overline{L}\left( (1-t_n)^{-1}\right) \end{aligned}$$

for a slowly varying function $\overline{L}$. In particular,

10.1 $$\begin{aligned} \frac{\mathbf {P}\left( \mathcal {H}(\mathcal {T}^{*-})> n\right) }{(1-s_n)}&= \frac{q}{1-q}\left( \frac{1-f(t_n)}{1-t_n}-f'(q)+f''(z)q(s_n-1)/2\right) \nonumber \\&=\frac{q}{1-q}\left( \mu -f'(q) +O\left( (1-s_n)^{\alpha -1}\overline{L}((1-s_n)^{-1})\right) \right) \nonumber \\&\sim \frac{q(\mu -f'(q))}{1-q} \end{aligned}$$

which is the desired result.$\square$

Recall that $\Delta _n^Y$ is the first hitting time of level *n* of the backbone by $Y_n$ and $L_x$ is the collection of traps adjacent to *x* then for $\varepsilon >0$ let

$$\begin{aligned} h_{n,\varepsilon }:=\left\lceil \frac{(1-\varepsilon )\log (n)}{\log (f'(q)^{-1})}\right\rceil \quad \text {and} \quad B(n):=\bigcap _{i=0}^{\Delta _n^Y}\left\{ \left| \left\{ T \in L_{Y_i}: \mathcal {H}(T)\ge h_{n,\varepsilon }\right\} \right| \le 1\right\} \end{aligned}$$

denote the critical height of a trap and the event that any backbone vertex seen up to $\Delta _n$ has at most one $h_{n,\varepsilon }$-trap (which is $C_3(n)$ of [4]) respectively.

### Lemma 10.2

Under the assumptions of Theorem 5

$$\begin{aligned} \mathbb {P}(B(n)^c)=o(1). \end{aligned}$$

### Proof

Using $C_1(n)$ from [4] we have that the number of backbone vertices visited by $\Delta _n$ is at most *Cn* with high probability therefore

$$\begin{aligned} \mathbb {P}\left( B(n)^c\right) \le o(1) +Cn\left( \mathbf {P}\left( \mathcal {H}(\mathcal {T}^{*-})>h_{n,\varepsilon }\right) -\mathbf {P}(\left| \left\{ T \in L_\rho : \mathcal {H}(T)\ge h_{n,\varepsilon }\right\} \right| = 1)\right) . \end{aligned}$$

Recall $s_{h_{n,\varepsilon }}=\mathbf {P}(\mathcal {H}(\mathcal {T}^-)< h_{n,\varepsilon })$ and from (10.1) we have that

$$\begin{aligned} \mathbf {P}\left( \mathcal {H}(\mathcal {T}^{*-})> h_{n,\varepsilon }\right) =(1-s_{h_{n,\varepsilon }}) \frac{q\left( \mu -f'(q)\right) }{1-q}+O(\left( 1-s_{h_{n,\varepsilon }}\right) ^\alpha \overline{L}\left( (1-s_{h_{n,\varepsilon }})^{-1}\right) \end{aligned}$$

for some slowly varying function $\overline{L}$.

Similarly to the method used in Lemma 10.1 we have that

$$\begin{aligned}&\mathbf {P}\left( \left| \left\{ T \in L_\rho : \mathcal {H}(T)\ge h_{n,\varepsilon }\right\} \right| = 1\right) \\&\quad = \sum _{k=1}^\infty \sum _{j=1}^{k-1}\mathbf {P}\left( Z_1=k,V_1=k-j,\left| \left\{ T \in L_\rho : \mathcal {H}(T)\ge h_{n,\varepsilon }\right\} \right| = 1\right) \\&\quad = \sum _{k=1}^\infty \mathbf {P}(Z_1=k)\sum _{j=1}^{k-1}\mathbf {P}\left( V_1=k-j\big |Z_1=k\right) j \left( 1-s_{h_{n,\varepsilon }}\right) s_{h_{n,\varepsilon }}^{j-1}\\&\quad = \sum _{k=1}^\infty \frac{(1-q^k)p_k}{1-q}\sum _{j=1}^{k-1}\left( {\begin{array}{c}k\\ j\end{array}}\right) \frac{q^j(1-q)^{k-j}}{1-q^k}j\left( 1-s_{h_{n,\varepsilon }}\right) s_{h_{n,\varepsilon }}^{j-1}\\&\quad = \frac{q\left( 1-s_{h_{n,\varepsilon }}\right) }{1-q}\sum _{k=1}^\infty kp_k\sum _{j=0}^{k-2}\frac{(k-1)!\left( qs_{h_{n,\varepsilon }}\right) ^j (1-q)^{k-1-j}}{j!(k-1-j)!} \\&\quad = \frac{q\left( 1-s_{h_{n,\varepsilon }}\right) }{1-q}\sum _{k=1}^\infty kp_k\left( \left( qs_{h_{n,\varepsilon }}+1-q\right) ^{k-1} -\left( qs_{h_{n,\varepsilon }}\right) ^{k-1}\right) \\&\quad = \frac{q\left( 1-s_{h_{n,\varepsilon }}\right) }{1-q} \left( f'\left( t_{h_{n,\varepsilon }}\right) -f'\left( qs_{h_{n,\varepsilon }}\right) \right) \end{aligned}$$

where $t_{h_{n,\varepsilon }}=qs_{h_{n,\varepsilon }}+1-q$. By Taylor $f'(qs_n)=f'(q)+O(1-s_{h_{n,\varepsilon }})$ as $n \rightarrow \infty$. From Lemma 3.3 we have that $1-f(t_{h_{n,\varepsilon }})=\mu (1-t_{h_{n,\varepsilon }})+(1-t_{h_{n,\varepsilon }})^\alpha \overline{L}((1-t_{h_{n,\varepsilon }})^{-1})$ for some slowly varying function $\overline{L}$. Applying Theorem 2 of [19] we have that $f'(t_{h_{n,\varepsilon }})=\mu +O((1-t_{h_{n,\varepsilon }})^{\alpha -1} \overline{L}((1-t_{h_{n,\varepsilon }})^{-1}))$. In particular,

$$\begin{aligned}&\mathbf {P}\left( \left| \left\{ T \in L_\rho : \mathcal {H}(T)\ge h_{n,\varepsilon }\right\} \right| = 1\right) \\&\quad =\frac{q\left( 1-s_{h_{n,\varepsilon }}\right) }{1-q} \left( \mu -f'(q)+O\left( \left( 1-t_{h_{n,\varepsilon }}\right) ^{\alpha -1} \overline{L}\left( \left( 1-t_{h_{n,\varepsilon }}\right) ^{-1}\right) \right) \right) \end{aligned}$$

since $$\alpha <2$$, thus

$$\begin{aligned} \mathbb {P}(B(n)^c) \le o(1) +O\left( n\left( 1-t_{h_{n,\varepsilon }}\right) ^{\alpha } \overline{L}\left( \left( 1-t_{h_{n,\varepsilon }}\right) ^{-1}\right) \right) . \end{aligned}$$

There exists some constant *c* such that $1-t_{h_{n,\varepsilon }}\sim qc_\mu f'(q)^{h_{n,\varepsilon }}\le c n^{-(1-\varepsilon )}$ therefore since $\alpha >1$ we can choose $\varepsilon >0$ small enough (depending on $\alpha$) such that $\mathbb {P}(B(n)^c)=o(1)$. $\square$

Let $D(n):=\left\{ \max _{j\le \Delta _n^Y}\mathcal {H}(\mathcal {T}^{*-}_{Y_j})\le 4\log (n)/\log (f'(q)^{-1})\right\}$ be the event that all branches seen before reaching level *n* are of height at most $4\log (n)/\log (f'(q)^{-1})$.

### Lemma 10.3

Under the assumptions of Theorem 5

$$\begin{aligned} \mathbb {P}\left( D(n)^c \right) =O(n^{-2}). \end{aligned}$$

### Proof

By comparison with a biased random walk on $\mathbb {Z}$, standard large deviations estimates yield that for *C* sufficiently large $\mathbb {P}(\Delta _n^Y>Cn)=O(n^{-2})$. Using Lemma 10.1 we have that for independent branches $\mathcal {T}^{*-}_j$

$$\begin{aligned}&\mathbf {P}\left( \bigcup _{j=1}^{C_1n}\mathcal {H}\left( \mathcal {T}^{*-}_j\right)>\frac{4\log (n)}{\log \left( f'(q)^{-1}\right) }\right) \\&\quad \le C_1n \mathbf {P}\left( \mathcal {H}\left( \mathcal {T}^{*-}\right) >\frac{4\log (n)}{\log \left( f'(q)^{-1}\right) }\right) \le Cnf'(q)^{\frac{4\log (n)}{\log (f'(q)^{-1})}} = Cn^{-3}. \end{aligned}$$

$$\square $$

## Glossary

$$f(s):=\sum _{k=0}^\infty p_ks^k$$
p.g.f. of Galton–Watson process
$$\xi ,\xi ^*$$
Offspring and size-biased variables
$$\beta $$
Bias of the walk
$$\mu ,\sigma ^2,\alpha $$
Mean, variance and stability index of the offspring distribution
$$\gamma $$
Scaling exponent
$$T,\rho ,Z_n^T,\mathcal {H}(T)$$
A fixed tree, its root, $n{\text {th}}$ generation size and height
$$T_x,c(x),\overleftarrow{x},d_x$$
The descendent tree, children, parent and out-degree of a vertex *x*
$$\mathcal {T},\mathcal {Y}$$
GW-tree conditioned to survive and its backbone
$$\mathcal {T}^{*-}$$
A dummy branch
$$\mathcal {T}^\circ $$
A dummy *f*-GW-tree
$$\mathcal {T}^\leftarrow $$
*f*-GW-tree with an artificial ancestor
$$\mathcal {T}^\prec _n$$
*f*-GW-tree conditioned to be height *n*
$$\mathcal {T}^\prec $$
An infinite trap
$$\mathcal {T}^*,\overline{H}$$
Pruned dummy branch and its height
$$\mathcal {T}^+_j,\mathcal {T}^-_j$$, $$\rho ^+_j,\rho ^-_j$$
Large/small traps in the pruned dummy branch and their roots
$$\mathcal {T}_{i,j}^+$$
$j{\text {th}}$ large trap in the $i{\text {th}}$ large branch
$$\rho _i,(\rho _{i,j})_j$$
Backbone vertex in generation *i* and its buds
$$\delta _0,\delta _1,\ldots $$
Spine of $$\mathcal {T}^\prec $$
$$\delta ,\delta ^j$$
Deepest vertices in $\mathcal {T}^{*},\mathcal {T}^+_j$
*N*(*m*): Number of traps of height at least *m* in $\mathcal {T}^{*-}$
*N*, $$N^i$$
Number of large traps in a dummy branch and $i{\text {th}}$ large branch
$$s_m$$
Distribution of the height of an *f*-GW-tree
$$c_\mu $$
Constant satisfying tail asymptotic for the height of an *f*-GW-tree
$$\mathcal {R}^{j,k,l}$$
Duration of the $l{\text {th}}$ excursion from $\delta ^j$ to itself on the $k{\text {th}}$ excursion
$$X_n,Y_n$$
Biased random walk on $\mathcal {T}$ and the underlying walk on the backbone
$$\eta _k$$
Time change satisfying $Y_k=X_{\eta _k}$
$$\Delta _n,\Delta _n^Y$$
First hitting time of the $n{\text {th}}$ level of the backbone by $X_n$ and $Y_n$
$$\upsilon _k$$
Regeneration times for the walk
$$X_n^{(i)}, Y_n^{(i)} ; \; i\ge 1$$
Independent walks on $\mathcal {T}$ and corresponding backbone walks
$$\mathcal {R}_\infty $$
Time taken for an excursion from the deepest point in a trap to itself
$$p_1(H)$$
Probability that the walk reaches $\delta$ before $\rho$
$$p_2(H)$$
Probability of escaping the tree started at $\delta$
$$ P ^T$$, $$\mathbb {P}$$, $$\mathbf {P}$$
Quenched, annealed and environment laws
$$\mathbb {P}^{\scriptscriptstyle {K}}, \; \mathbf {P}^{\scriptscriptstyle {K}}$$
Annealed and environment laws conditioned on the number of buds or the height
*L*: Slowly varying function satisfying (2.2)
$$a_n$$
Scaling sequence for $\xi ^*$ so that $\mathbf {P}(\xi ^*\ge xa_n) \sim n^{-1}x^{-(\alpha -1)}$
$$\tilde{L}$$
Slowly varying function satisfying $a_n= n^{\frac{1}{\alpha -1}}\tilde{L}(n)$
$$n_l(t)$$
Subsequence for convergence
$$r_n$$, $$\overline{r}_n$$
Appropriate scaling of $\Delta _n$ in each case and its inverse
$$l_{n,\varepsilon },l_{n,\varepsilon }^+ $$
Critical number of buds in IVFE
$$h_{n,\varepsilon },h_{n,\varepsilon }^+$$
Critical height of a branch in FVIE and IVIE
$$\mathcal {D}^{(n)}$$
Roots of large branches
$$\mathcal {D}^{(n)}_m$$
Roots of large branches up to level *m*
*K*(*n*): Vertices in large traps
$$(\rho _i^+)_{i\ge 1}$$
Ordered roots of large branches
$$q_n$$
Probability that a backbone vertex is large
$$\overline{L}_{\scriptscriptstyle {K}}:=l_{n,0}+K$$
Number of traps in a large branch
$$\overline{H}_{\scriptscriptstyle {K}}:=h_{n,0}+K$$
Height of a large branch
$$b_n^{\scriptscriptstyle {K}}:=\mu ^{-\overline{H}_{\scriptscriptstyle {K}}}/c_\mu $$
Scaling for $\xi ^*$ conditioned on the height of the branch
$$\chi _{t,n}$$
Time spent up to $\Delta _{\lfloor nt \rfloor }$ in large traps
$$\chi ^i_n$$
Total time spent in large traps of the $i{\text {th}}$ large branch
$$\tilde{\chi }_n^i$$
Time spent in the $i{\text {th}}$ large branch by $X_n^{(i)}$
$$\tilde{\chi }_n$$
Dummy version of $\tilde{\chi }_n^i$ on $\mathcal {T}^{*-}$
$$\tilde{\chi }_{t,n}$$
Sum of $\tilde{\chi }_n^i$ up to $i=\lfloor nt q_n\rfloor$
$$\tilde{\chi }^\infty _n$$
Approximation of $$\tilde{\chi }_n$$
$$\tilde{\chi }^{i*}_n$$
Excursions from $\delta$ to $\delta$ in $\tilde{\chi }_n^i$
$$\chi ^*_n$$
Dummy decomposition of $\tilde{\chi }^{i*}_n$ on $\mathcal {T}^{*}$
$$\zeta ^{(n)}$$
$\tilde{\chi }_n$ scaled by the number of buds or $\beta ^{\overline{H}}$
$$Z_\infty ^n$$
Scaled excursion times
$$Z_\infty $$
Limit of $$Z_\infty ^{n_l}$$
$$T^{j,k}$$
The duration of the $k{\text {th}}$ excursion in the $j{\text {th}}$ trap of $\mathcal {T}^{*-}$
$$T^{(i,j,k)}$$
Duration of the $k{\text {th}}$ excursion in $\mathcal {T}^+_{i,j}$
$$T^{*(i,j,k)}$$
Duration of the excursions from $\delta$ to $\delta$ in the $k{\text {th}}$ excursion in $\mathcal {T}^+_{i,j}$
*d*(*x*, *y*): Graph distance between points *x*, *y*
$$|x|:=d(\rho ,x)$$
Graph distance between *x* and the root of the tree
$$\tau _x^+$$
First return time to vertex *x*
